# Effects of *TmTak1* silencing on AMP production as an Imd pathway component in *Tenebrio molitor*

**DOI:** 10.1038/s41598-023-45978-4

**Published:** 2023-11-02

**Authors:** Su Hyeon Hwang, Ho Am Jang, Maryam Ali Mohammadie Kojour, Keunho Yun, Yong Seok Lee, Yeon Soo Han, Yong Hun Jo

**Affiliations:** 1 Division of Plant Biotechnology, Institute of Environmentally-Friendly Agriculture (IEFA), College of Agriculture and Life Sciences, Chonnam National University, Gwangju, 61186 Republic of Korea; 2 Department of Biology, College of Natural Sciences, Soonchunhyang University, Asan, Chungnam, Republic of Korea; 3 Korea Native Animal Resources Utilization Convergence Research Institute (KNAR), Soonchunhyang University, Asan, Chungnam, Republic of Korea

**Keywords:** Immunology, Innate immunity

## Abstract

Mealworms beetles, *Tenebrio molitor*, are the limelight next-generation food for humans due to their high nutrient contents. Since *Tenebrio molitor* is used as feed for pets and livestock in addition to their ability to decompose polystyrene and plastic waste, it is recognized as an insect with an industrial core value. Therefore, it is important to study the immune mechanism related to the development and infection of mealworms for mass breeding purposes. The immune deficiency (Imd) signaling is one of the main pathways with pivotal roles in the production of antimicrobial peptides (AMPs). Transforming growth factor-β activated kinase (TAK1) is one of the Imd pathway components, forms a complex with TAK1 binding protein 2 (TAB2) to ultimately help activate the transcription factor Relish and eventually induce host to produce AMPs. Relatively, little has been revealed about TAK1 in insect models, especially in the *T. molitor*. Therefore, this study was conducted to elucidate the function of *TmTak1* in *T. molitor*. Our results showed that the highest and lowest mRNA expression of *TmTak1* were found in egg and young larvae respectively. The tissue-specific expression patterns were reported in the gut of *T. molitor* larvae and the fat bodies of adults. Systemic microbial challenge illustrated *TmTak1* high expression following the fungal infection in all dissected tissues except for the whole body. However, silencing *TmTak1* experiments showed that the survivability of *T. molitor* larvae affected significantly following *Escherichia coli* infection. Accordingly, AMP induction after *TmTak1* knock down was mainly reported in the integument and the fat bodies.

## Introduction

The yellow mealworm, *Tenebrio molitor* (*T. molitor*) is a well-known insect not only because of its high nutritional values but also as a crop pest^1,2^. *T. molitor* exposure to different pathogenic factors in their natural inhabitants has made them a great immune study model^3–5^. Considering *T. molitor* industrial importance, their relatively larger size, and their convenient rearing condition, it is important to discuss disease resistance defense mechanisms related to the development and health of mealworms^6,7^. Like other insects, yellow mealworms have unique physical and physiological mechanisms to prevent foreign invaders or reduce infection^3^. Among all the shields, *T. molitor* uses its innate immune arms to produces antimicrobial peptides (AMPs) through a specific mechanism^8–12^. Insects’ innate immunity perceive Lipopolysaccharide (LPS) and Peptidoglycan (PGN) from various microbial sources via their pattern recognition receptors (PRRs)^13,14^. Functional immune response is divided into cellular and humoral immunity^15^. Cellular immunity includes blood cell-mediated immune responses including phagocytosis, nodulation, and encapsulation^16^, whereas humoral immunity involves Toll, immune deficiency (Imd), and the Janus kinase/signal transducers and activators of transcription (JAK/STAT) pathways triggering immune related gene expression including but not limited to AMPs production^5,17–21^. While in *Drosophila* both Toll and Imd pathways are important, Imd pathway regulates most of the AMPs expression^18^. In *Drosophila*, peptidoglycan recognition proteins (PGRPs) which recognize bacterial cell wall compartments can be divided into PGRP-SA, PGRP-LC, and PGRP-LE^22^. Lys-type PGN, a cell wall component of Gram-positive bacteria, is recognized by PGRP-SA^23^, activates the Toll pathway, and induce AMPs production. On the other hand, DAP-type PGN, a cell wall component of Gram-negative bacteria, is recognized by PGRP-LC and LE^24^ and forms a complex, which activates the Imd pathway and induces an immune response through a complex pathway leading to AMP production.

The Imd pathway is activated by forming a complex containing Fas-associated death domain protein (FADD) and death-related ced-3/Nedd2-like protein (DREDD). DREDD is a caspase that cleaves the Imd protein and creates a new binding site for Inhibitor of apoptosis-2 (IAP-2). Then, the E2-ubiquitin complex binds to the IAP-2 binding site and forms a Transforming growth factor-β (TGF-β) activated kinase (TAK1)/TAK1 binding protein2 (TAB2) complex. The TAK1/TAB2 complex then induces phosphorylation of the inhibitor of Nuclear factor-κB kinase (IKK) complex to activate Relish. Subsequently, activated Relish mediates transcription of the AMP genes^5^. Studies on *Drosophila* TAK1 mutations revealed that TAK1 plays role in Gram-negative bacteria infection by activating IKK complex^25^.

The Imd pathway is often compared to mammalian tumor necrosis factor (TNF) signaling^26^. Essentially, activation of the Imd pathway and TNF-α target genes require activation of the transcription factors Relish and Nuclear factor kappa-light-chain-enhancer of activated B cells (NF-κB) respectively^26^. In mammalian NF-κB signaling, activation of transcription factors occurs through a pathway in which the Inhibitor of NF-κB (IκB) kinase complex phosphorylates IκB and the phosphorylated IκB is degraded to activate NF-κB^27^. It has been demonstrated that TAK1 is required for activation of NF-κB following TNF or Interleukin-1 (IL-1) stimulation in mammals^28–30^.

TAK1 is a kinase that can be activated by TGF-β and was first discovered in 1995^31^. It has also been found to be an important mediator of inflammatory responses activated by LPS, TNF-α, and IL-1^30,32,33^, and it plays an important role in immune responses in mammals and insects.

Until recently, most studies on the Imd pathway were confined to *Drosophila*^34^ and few similar studies have been conducted in other insect models. Here, we investigated immunological roles of *TmTak1*, an understudied compartment in Imd pathway involves in humoral immunity, in *T. molitor*.

## Result

### In silico analysis of *Tm*TAK1

As a result of homology mapping of the *TmTak1* cDNA sequence based on the *T. molitor* DNA-seq database, 8 exons intervening with 7 introns were identified (Supplementary Fig. 1). Among the 8 exons of *TmTak1*, exon 2 was the longest with 330 sequences, followed by exon 4 with 249 nucleotide sequences and exon 8 with 220 nucleotide sequences. Among the 7 introns, intron 6 was longer than the other introns, so the size of the introns was not uniform. In addition, the coding sequence consisted of nucleotides of 1527 bp and coded 508 amino acid residues. The full-length coding sequence of *TmTak1* was submitted to GenBank, accession number OR373077 has been granted. As a result of protein domain analysis of *Tm*TAK1, it was confirmed that the protein tyrosine kinase domain from amino acid residues no. 24 to 256 and four tyrosine kinase catalytic domains were located at no. 93 to 106, no. 134 to 152, no. 179 to 189, and no. 247 to 269 residues (Supplementary Fig. 2). TAK1 orthologues and Multiple Sequence Alignment (MSA) of insect was analyzed using ClustalX v2.0. Through the MSA results, it was confirmed that protein tyrosine kinase and tyrosine kinase catalytic domains in the *Tm*TAK1 amino acid sequence is well conserved in other insects (Supplementary Fig. 3). The phylogenetics of *Tm*TAK1 were analyzed using TAK1 homologues from other insect species (Supplementary Fig. 4). *Tm*TAK1 clustered with *Tc*MKKK7 which has 85% sequence identity with *Tm*TAK1 and was isolated under the same cluster as other coleopteran species.

### Developmental and tissue-specific expression patterns of *TmTak1*

**Figure 1:**
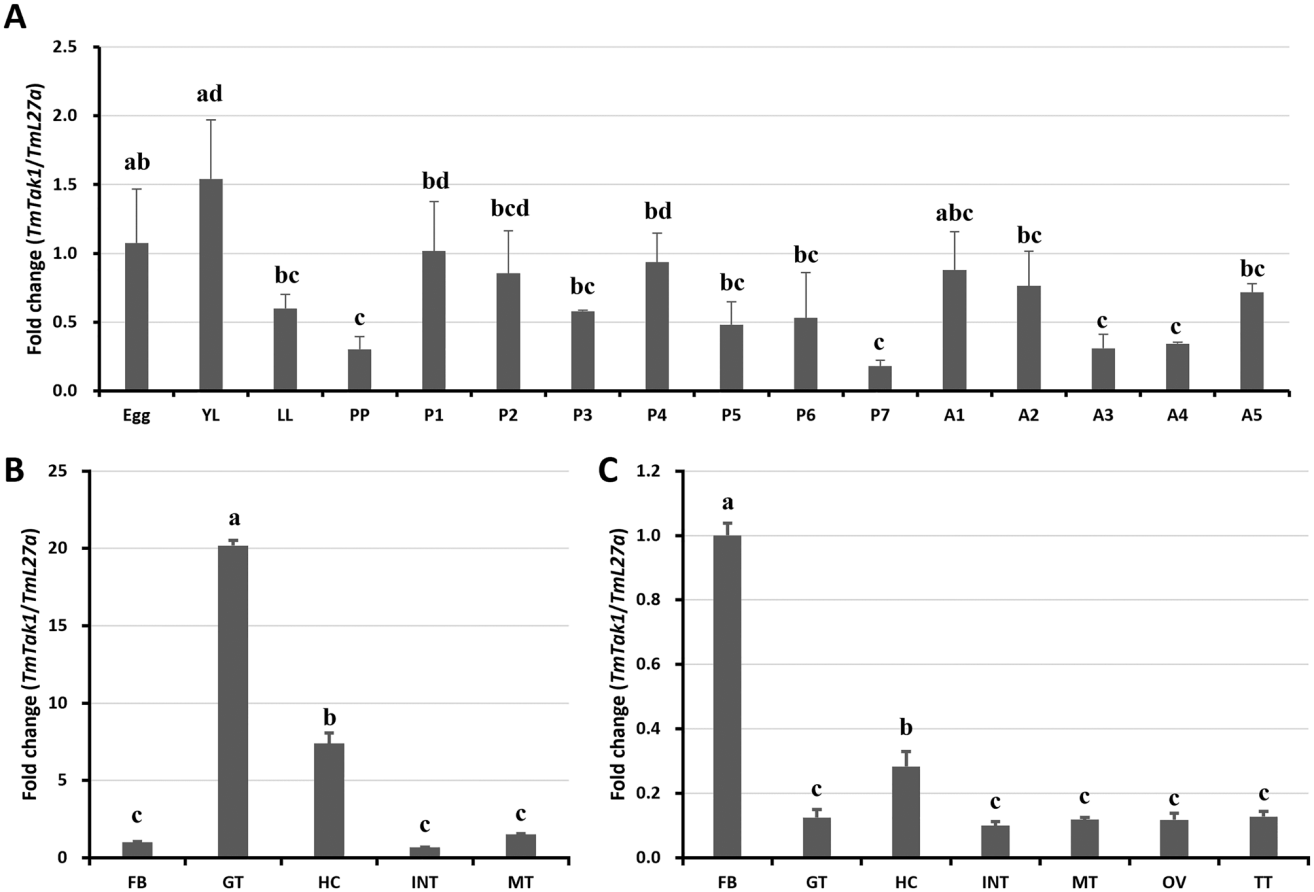
Relative expression levels of *TmTak1* mRNA in developmental stages and tissues of *T. molitor.* (**A**) Expression levels of *TmTak1* in *T. molitor* at the egg, the young larvae (YL), late larvae (LL), pre-pupae (PP), 1–7-day-old pupae (P1–7), and 1–5-day-old adult (A1–5) stages. Distribution of *TmTak1* transcripts in larva and adult tissues. Fat bodies (FB), gut (GT), hemocytes (HC), integument (INT), and Malpighian tubules (MT) of (**B**) late instar larvae and (**C**) adults, in addition to ovaries (OV) and testes (TT) of adults, were dissected and collected from 20 late larvae and 5-day-old adults for analysis. *T. molitor* 60S ribosomal protein L27a (TmL27a)-encoding gene was used as an internal control.

During the development of *T. molitor*, the expression pattern of *TmTak1* was investigated by qPCR. As a result, the expression level of *TmTak1* was highest in young larvae (YL) and lowest in 7-day old pupae (P7) (Fig. 1A). As a result of examining the tissue-specific expression pattern of *TmTak1* in *T. molitor*, high expression level of *TmTak1* was confirmed in the gut (GT) of the larval stage and low expression level was confirmed in the integument (INT) (Fig. 1B). Next, in the adult stage, the expression level of *TmTak1* was the highest in the fat bodies (FBs), and low expression levels were confirmed in the other tissues (Fig. 1C).

### *TmTak1* mRNA expression level after microbial challenge

**Figure 2:**
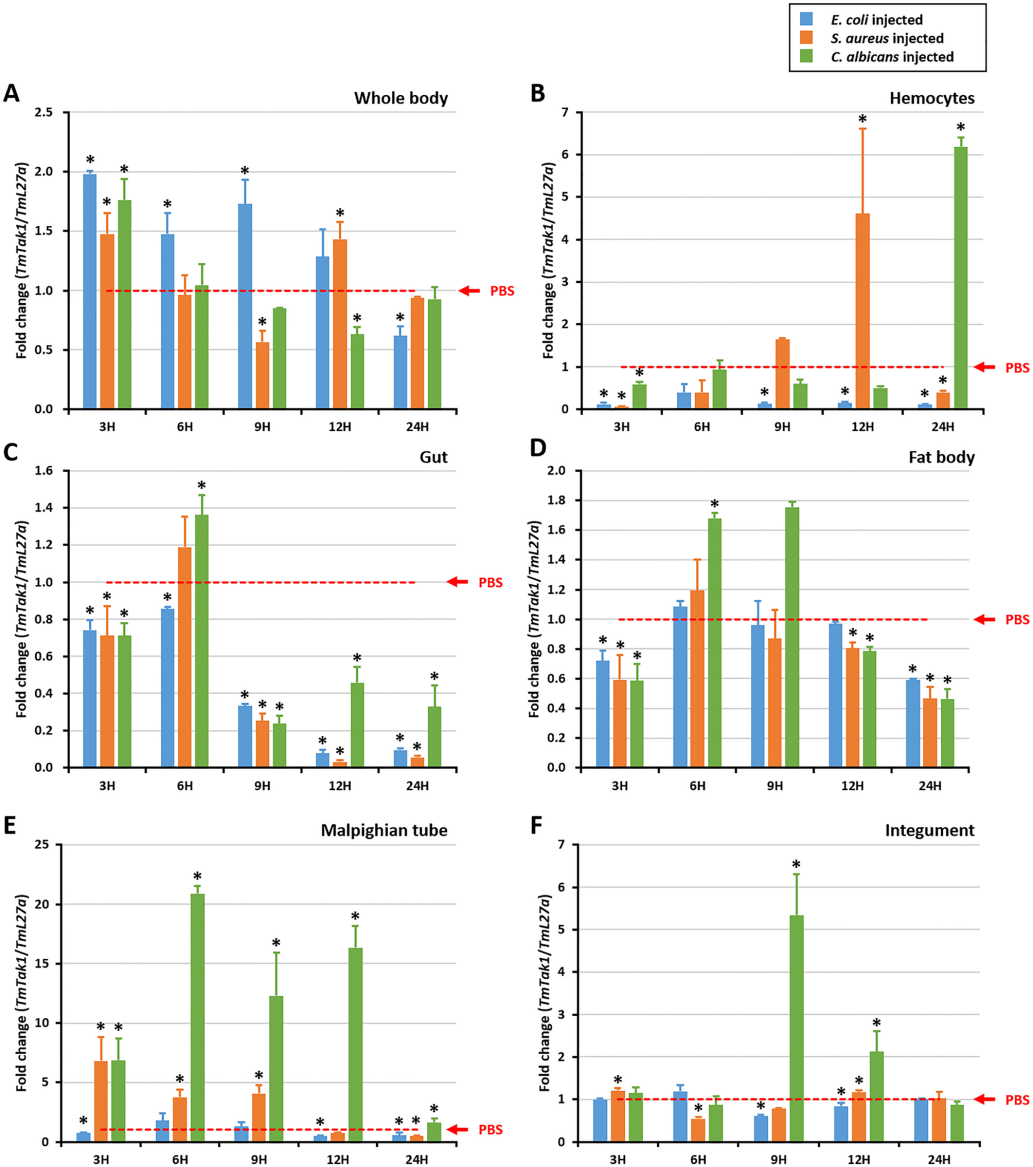
*TmTak1* mRNA expression profiles after microbial challenge. Expression of *TmTak1* mRNA in the whole body (**A**), hemocytes (**B**), gut (**C**), fat bodies (**D**), Malpighian tubules (**E**), integument (**F**) of larvae infected with *Escherichia coli*, *Staphylococcus aureus*, and *Candida albicans*. The expression was analyzed by qPCR using *L27a* (*T. molitor*) as the internal control. For each time point, the expression level in the phosphate-buffered saline (PBS)-injected control (mock control) was set to 1; this is represented by a dotted line. vertical bars represent mean ± standard error from three biological replicates.

*T. molitor* larvae were infected with PBS, *E. coli*, *S. aureus*, and *C. albicans*, and after 3 h, 6 h, 9 h, 12 h, and 24 h, WB and five tissues (GT, FBs, MTs, INT, and HCs) were dissected (Fig. 2). *TmL27a* was used as a house keeping gene, and the relative expression level of *TmTak1* was investigated using qPCR using PBS as a control. As a result of examining the expression level, high expression levels specifically for *C. albicans* were confirmed in the gut, fat bodies, Malpighian tubules, integument, and hemocytes compared to *E. coli* in whole body. In particular, the expression level of *TmTak1* was relatively higher in MTs than other tissues against *C. albicans* infection (Fig. 2E).

### *TmTak1* knockdown increased the mortality of *E. coli*-injected *T. molitor* larvae

**Figure 3:**
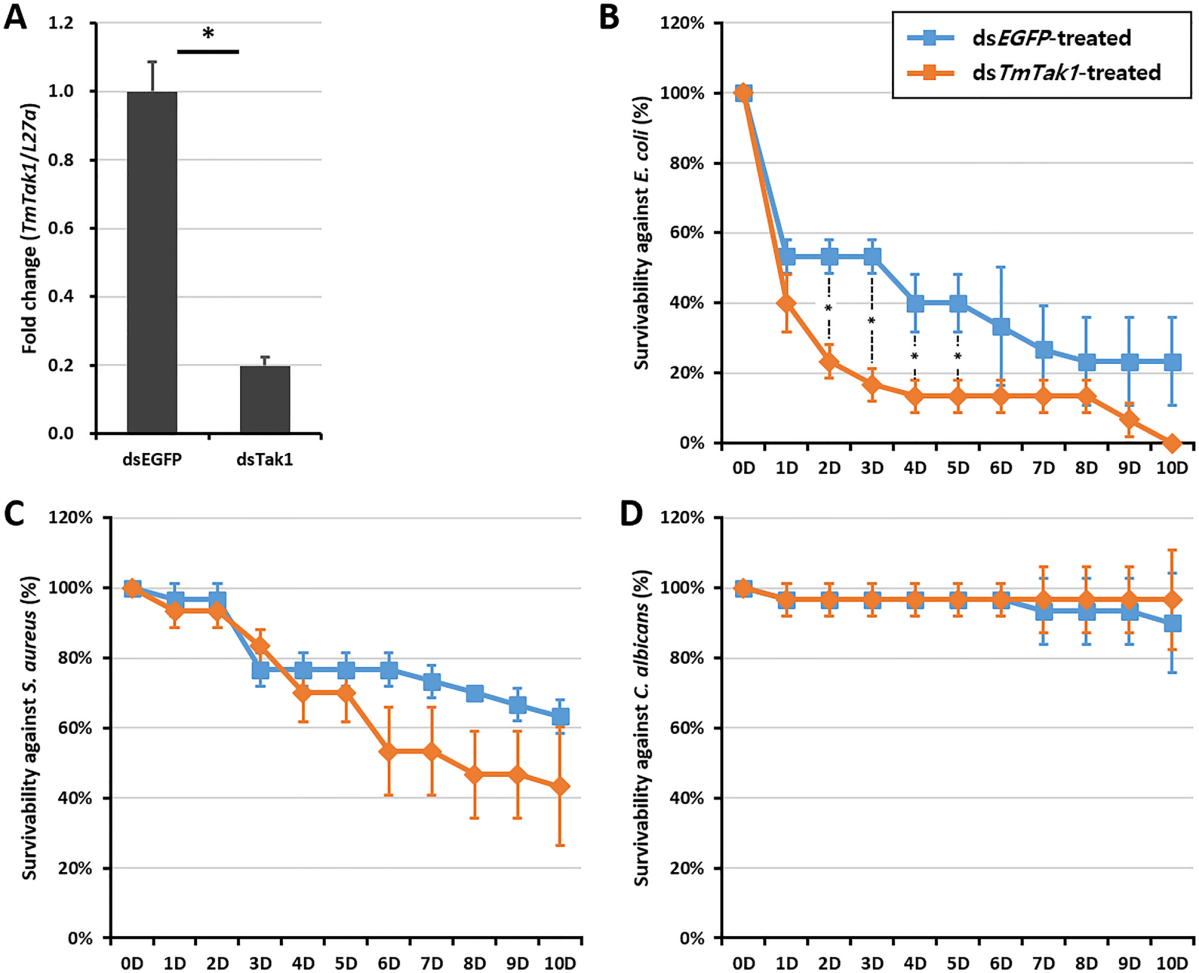
Effect of *TmTak1* RNAi on *T. molitor* Larval Survival. (**A**) *TmTAK1* knockdown efficiency measured using RT-qPCR at day 5 post-injection. The survivability of larva was measured after *TmTak1* knockdown and infection with *Escherichia coli* (**B**), *Staphylococcus aureus* (**C**), or *Candida albicans* (**D**) (n = 30). Data are presented as average of three biologically independent replicate experiments. Asterisks indicate significant differences between *TmTak1*-knocked down and ds*EGFP*-treated groups (*p* < 0.05). Survival rates were analyzed based on the Kaplan–Meier plots (log-rank chi-square test; *p < 0.05).

The mortality was investigated after RNAi using ds*TmTak1* in *T. molitor* (Fig. 3A). The concentration of bacteria used in the experiment was 1 × 10^7^ CFU/μl for *E. coli* and *S. aureus*, and 5 × 10^5^ CFU/μl l for *C. albicans*, and n = 10 were repeated three times. It was confirmed up to the 10th day after infection with the microorganisms (Fig. 3B–D). There was no significant difference between *S. aureus* and *C. albicans*, but after *E. coli* infection, the survival rate of the group injected with ds*TmTak1*, the experimental group, was reduced to about 10% on the first day and 35% on the second day, compared to the control group injected with ds*EGFP* (Fig. 3B).

### Effects of *TmTak1* RNAi on AMP expression

After knockdown of *TmTak1* using ds*TmTak1*, the amount of AMP expression was examined 24 h after systemic infection in each. GT, INT, MTs, and HCs, were the major tissues in which AMP expression was reduced after gene silencing of *TmTak1*.

**Figure 4:**
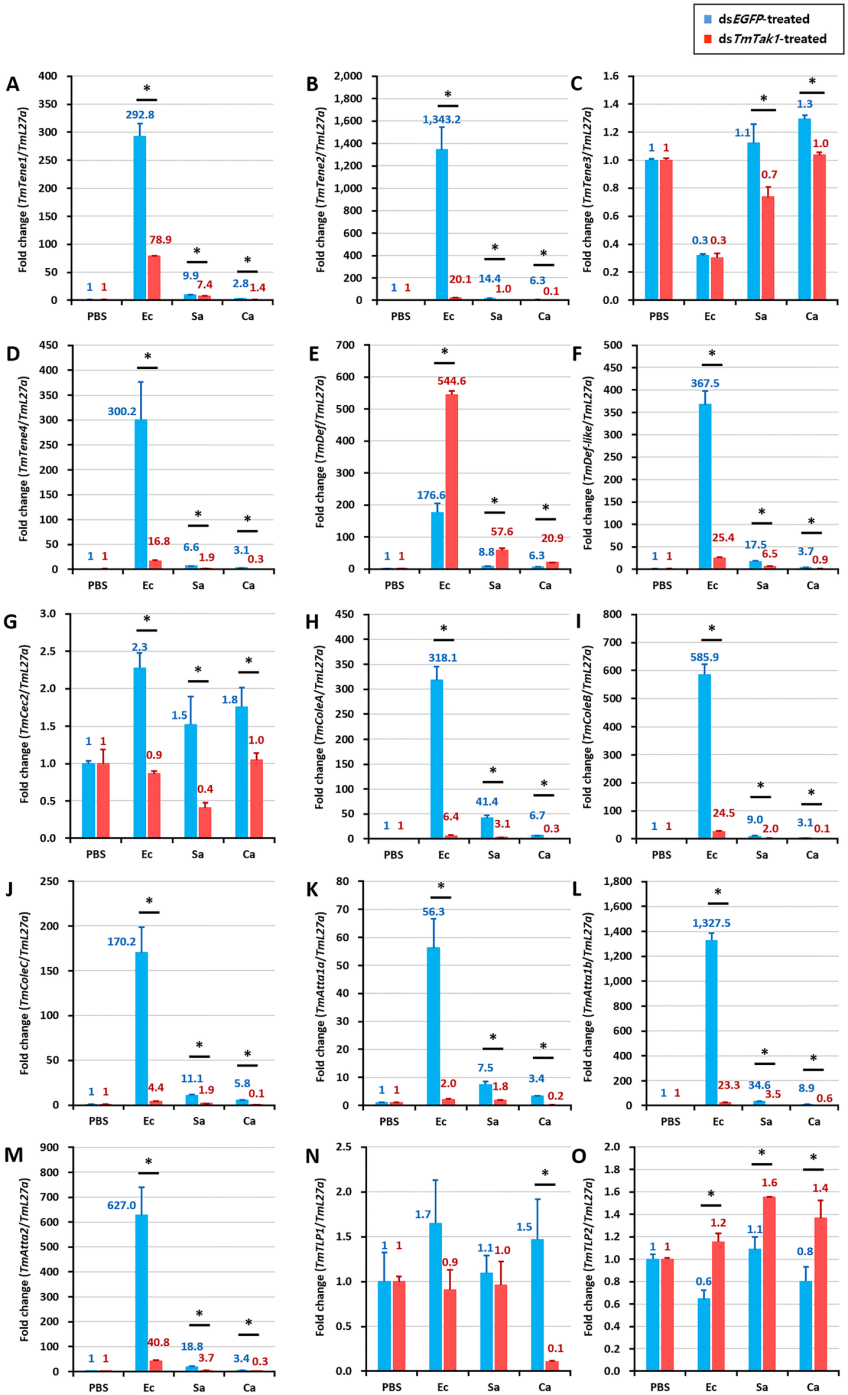
AMPs mRNA expression levels in the gut against *E. coli*, *S. aureus*, and *C. albicans* after *TmTak1* knockdown. To evaluate the functional properties of *TmTak1* in regulating AMP genes expression in response to pathogens, we used ds*TmTak1*, the amount of AMP expression was examined 24 h after systemic infection. AMP genes, including *TmTene1* (**A**), *TmTene2* (**B**), *TmTene3* (**C**), *TmTene4* (**D**), *TmDef* (**E**), *TmDef-like* (**F**), *TmCec2* (**G**), *TmColeA* (**H**), *TmColeB* (**I**), *TmColeC* (**J**), *TmAtt1a* (**K**), *TmAtt1b* (**L**), *TmAtt2* (**M**), *TmTLP1* (**N**), and *TmTLP2* (**O**).

**Figure 5:**
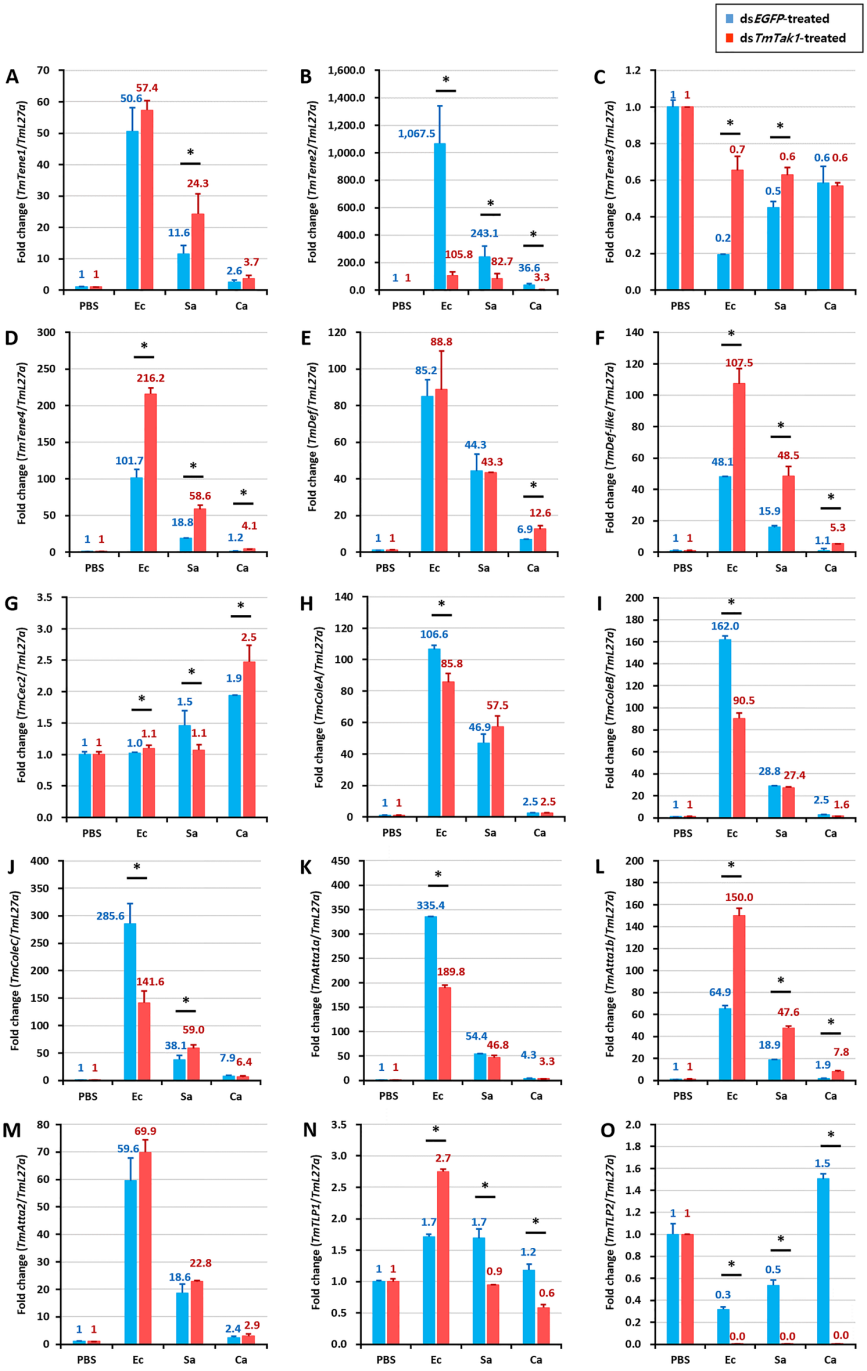
AMPs mRNA expression levels in the integument against *E. coli*, *S. aureus*, and *C. albicans* after *TmTak1* knockdown. To evaluate the functional properties of *TmTak1* in regulating AMP genes expression in response to pathogens, we used ds*TmTak1*, the amount of AMP expression was examined 24 h after systemic infection. AMP genes, including *TmTene1* (**A**), *TmTene2* (**B**), *TmTene3* (**C**), *TmTene4* (**D**), *TmDef* (**E**), *TmDef-like* (**F**), *TmCec2* (**G**), *TmColeA* (**H**), *TmColeB* (**I**), *TmColeC* (**J**), *TmAtt1a* (**K**), *TmAtt1b* (**L**), *TmAtt2* (**M**), *TmTLP1* (**N**), and *TmTLP2* (**O**).

**Figure 6:**
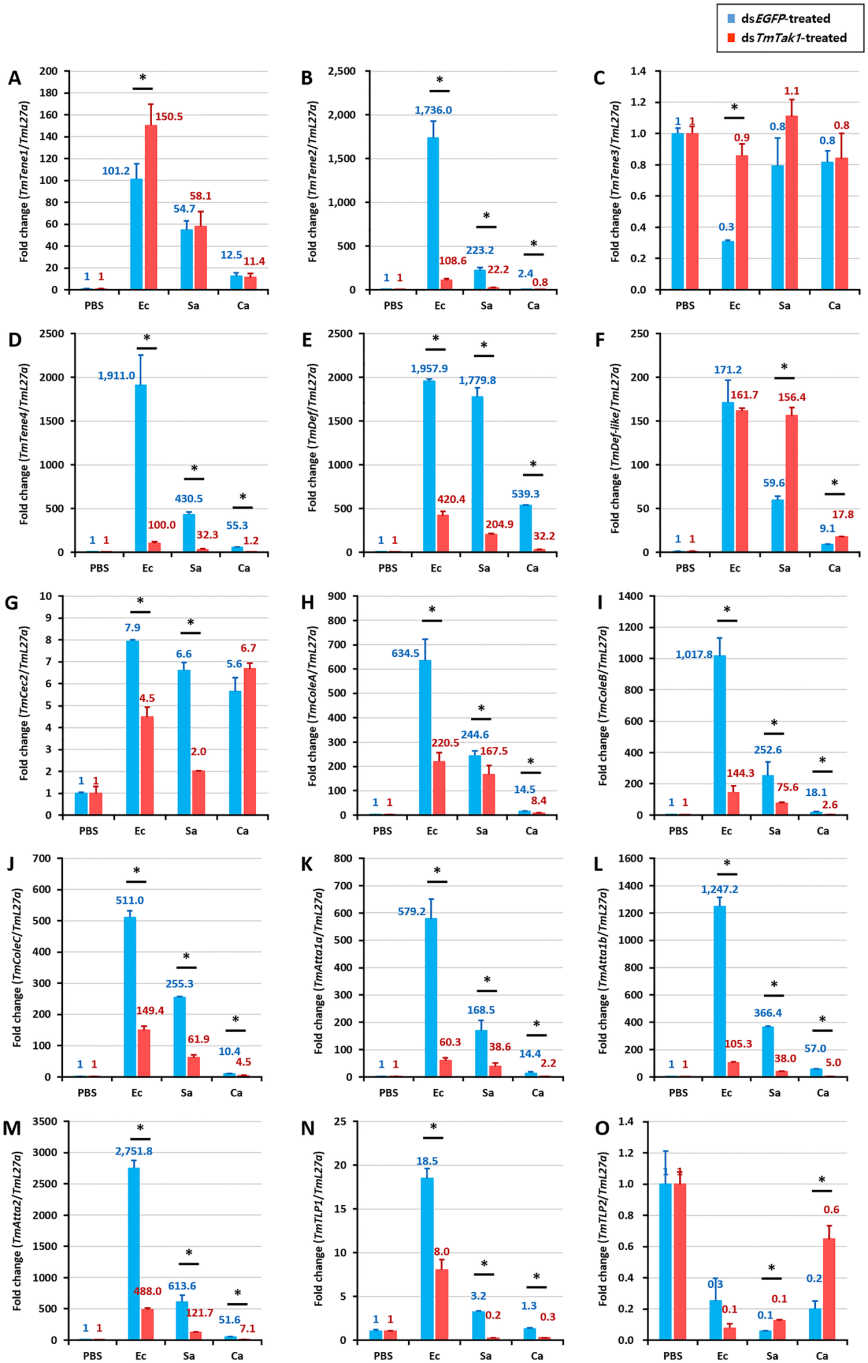
AMPs mRNA expression levels in the Malpighian tubules against *E. coli*, *S. aureus*, and *C. albicans* after *TmTak1* knockdown. To evaluate the functional properties of *TmTak1* in regulating AMP genes expression in response to pathogens, we used ds*TmTak1*, the amount of AMP expression was examined 24 h after systemic infection. AMP genes, including *TmTene1* (**A**), *TmTene2* (**B**), *TmTene3* (**C**), *TmTene4* (**D**), *TmDef* (**E**), *TmDef-like* (**F**), *TmCec2* (**G**), *TmColeA* (**H**), *TmColeB* (**I**), *TmColeC* (**J**), *TmAtt1a* (**K**), *TmAtt1b* (**L**), *TmAtt2* (**M**), *TmTLP1* (**N**), and *TmTLP2* (**O**).

**Figure 7:**
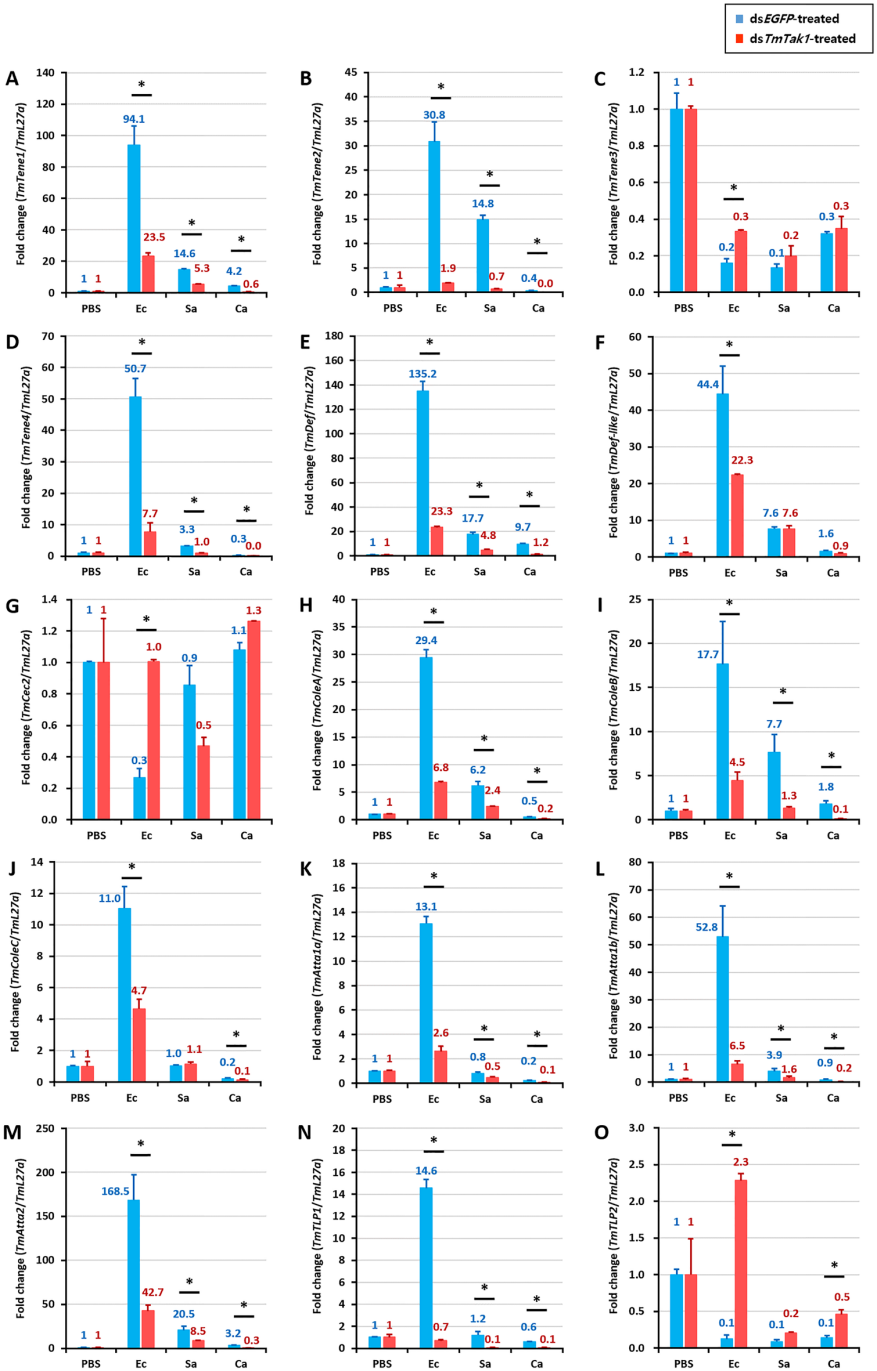
AMPs mRNA expression levels in the hemocytes against *E. coli*, *S. aureus*, and *C. albicans* after *TmTak1* knockdown. To evaluate the functional properties of *TmTak1* in regulating AMP genes expression in response to pathogens, we used ds*TmTak1*, the amount of AMP expression was examined 24 h after systemic infection. AMP genes, including *TmTene1* (**A**), *TmTene2* (**B**), *TmTene3* (**C**), *TmTene4* (**D**), *TmDef* (**E**), *TmDef-like* (**F**), *TmCec2* (**G**), *TmColeA* (**H**), *TmColeB* (**I**), *TmColeC* (**J**), *TmAtt1a* (**K**), *TmAtt1b* (**L**), *TmAtt2* (**M**), *TmTLP1* (**N**), and *TmTLP2* (**O**).

In the GT, mRNA expression of ten AMPs including *TmTene1*, *2*, *4*, *TmDef-like*, *TmColeA*, *B*, *C*, *TmAtta1a*, *1b*, and *2* were significantly decreased by *TmTak1* knockdown after *E. coli* (Fig. 4). *TmTene2* and *TmAtta1b* were the AMPs most affected by *TmTak1* gene silencing in the GT (Fig. 4B, L). In the INT, although the number of decreased AMP expression is smaller than that of the three tissues mentioned above, four AMPs showed most significantly reduced mRNA expression are including *TmTene2*, *TmColeB*, *C*, and *TmAtta1a* were the AMPs most affected by *TmTak1* gene silencing (Fig. 5). In the MTs, mRNA expression of ten AMPs including *TmTene2*, *4*, *TmDef*, *TmColeA*, *B*, *C*, *TmAtta1a*, *1b*, *2*, and *TmTLP1* were significantly decreased by *TmTak1* knockdown after *E. coli* (Fig. 6). Six AMPs showed most significantly reduced mRNA expression in the MTs are including *TmTene2*, *4*, *TmDef*, *TmColeB*, *TmAtta1b* and *2* were the AMPs most affected by *TmTak1* gene silencing (Fig. 6B, D, E, I, L, M). In the HCs, mRNA expression of twelve AMPs including *TmTene1*, *2*, *4*, *TmDef*, *TmDef-like*, *TmColeA*, *B*, *C*, *TmAtta1a*, *1b*, *2*, and *TmTLP1* were significantly decreased by *TmTak1* knockdown after *E. coli* (Fig. 7). *TmTene1*, *TmDef* and *TmAtta2* were the AMPs most affected by *TmTak1* gene silencing in the HCs (Fig. 7E, M).

### Effects of *TmTak1* RNAi on the expression of *Tenebrio* NF-κB genes

**Figure 8:**
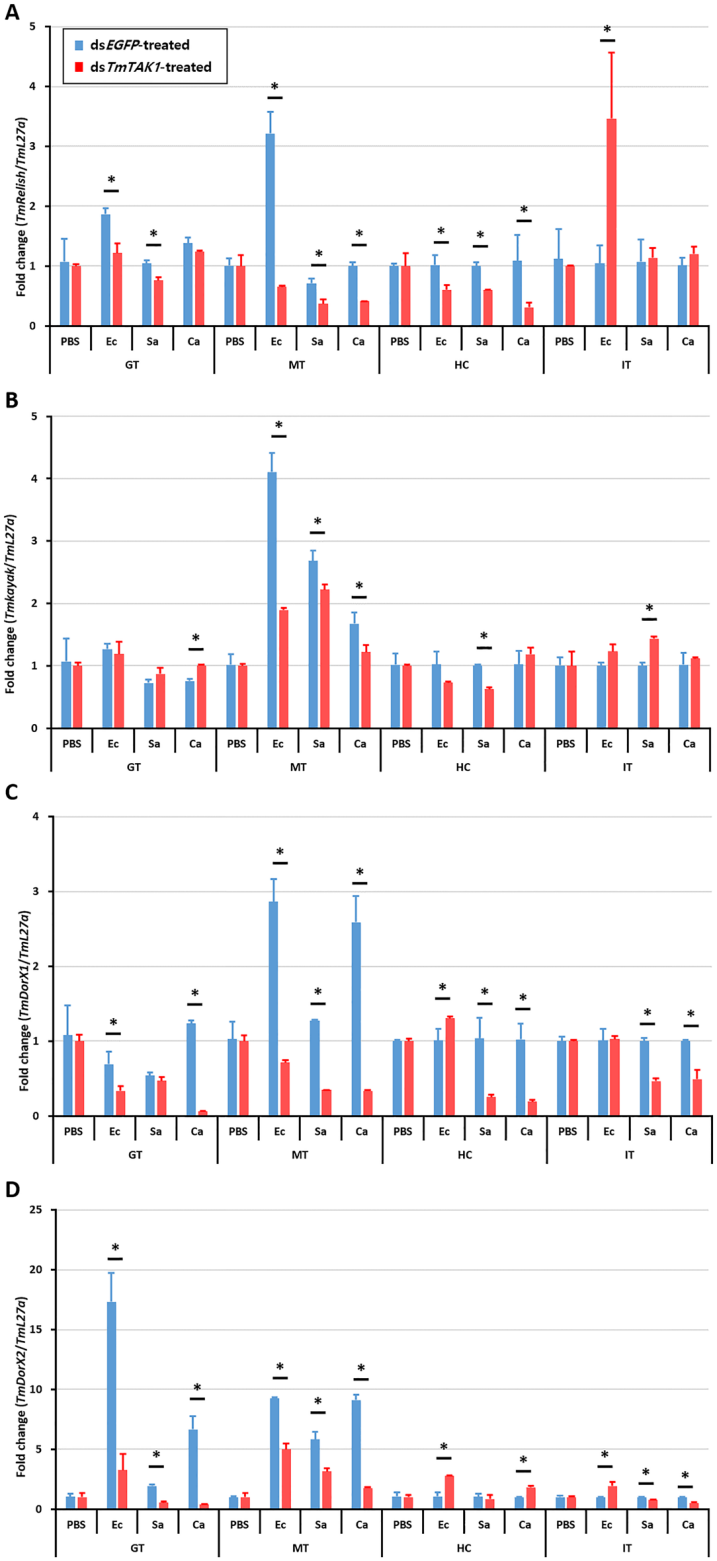
Effects of *TmTak1* RNAi on NF-κB genes. To investigated the effect of ds*TmTak1* on the expression of NF-κB genes, including *TmRelish* (**A**)*, TmKayak* (**B**), *TmDorX1* (**C**), and *TmDorX2* (**D**). Gut (GT), Malpighian tubules (MT), hemocytes (HC), and integument (INT) were dissected and collected from 20 early larvae for analysis. *T. molitor* 60S ribosomal protein L27a (TmL27a)-encoding gene was used as an internal control.

The expression of *T. molitor* NF-κB genes implicated in the IMD (*TmRelish*), JNK (*TmKayak*), and Toll (*TmDorX1* and *TmDorX2*) pathways were studied in *TmTak1*-silenced individuals upon microbial challenge (Fig. 8). The study was conducted to address the antimicrobial activity of *TmTak1*. The mRNA expression of *TmRel* and *TmKay* transcripts were significantly decreased in the MTs, GT, and HCs of *TmTak1*-silenced *T. molitor* in a descending manner. Conversely, relevant expression of *TmRel* upregulated in the *TmTak1* silenced INT and FBs, suggesting involvement of *TmTak1* in the canonical pathway of AMP production which effects *TmRel* mRNA expression MTs, GT, and HCs. Furthermore, the relevant downregulation of *TmRel* and *TmKay* mRNA expression was more significant after *E. coli* challenge compared to *S. aureus* and *C. albicans* challenge. Regarding the *TmDorX1* and *TmDorX2* identical result was reported, while the most significant depletion of transcription factor expression was related to *TmDorX2* following *E. coli* challenge in the GT of silenced larvae. Accordingly, these results justified the significant mortality of the *T. molitor* larvae following *E. coli* challenge.

## Discussion

The innate immune system is the first line of defense against invading pathogens and is comprised of a variety of cellular and humoral components^35^. In terms of immunological effects, TAK1 has been shown to play a critical role in the regulation of the innate immune response in insects^20,25,36–38^. In this study, we found that TAK1 might be as regulator Imd and JNK signaling pathway which response to pathogenic infection such as *E. coli*.

TAK1 is a serine/threonine kinase that is involved in a wide range of signaling pathways in various organisms, including mammalian and insects^39–41^. In mammalian, TAK1 is complexed with adaptor proteins TAB1, TAB2 and TAB3. TAB1 interact with TAK1 and triggers autophosphorylation of TAK1, which is essential for TAK1 kinase activity. TAB2 and TAB3 are involved in complex structure and share 48% of both amino acid sequence. TAB1 binds to the kinase domain in N-terminus of TAK1, and TAB2 and TAB3 bind to the C-terminal region of TAK1^42^. Complex between the kinase domain of TAK1 and the C-terminal TAB1 stimulates phosphorylation-dependent TAK1 activation^43^. Herein, we found *Tm*TAK1 includes the protein tyrosine kinase domain from amino acid residues no. 24 to 256 and four tyrosine kinase catalytic domains were located at no. 93 to 106, no. 134 to 152, no. 179 to 189, and no. 247 to 269 residues (Supplementary Fig. 2). Further, the sequence alignment results revealed the presence of homologs of TAK1 in many insect species, this study assigned Odonata, Isoptera, Hemiptera, Hymenoptera, Coleoptera, Lepidoptera, and Diptera (Supplementary Fig. 3) and phylogenetic characteristics of *Tm*TAK1 disclose adjacent association within the Coleoptera (Supplementary Fig. 4).

In this study, mRNA of *TmTak1* was expressed in all developmental stage and the highest level of expression was detected in Egg and YL stages (Fig. 1A). In *Xenopus* embryos, *x*TAK1 and *x*TAB1 is relative with ventral mesoderm development. The ventralization caused by constitutively active the bone morphogenetic protein receptor IA (BMPR-IA) and Smad1/5 is reversed by the kinase-negative form of *x*TAK1. Ectopic expression of *xTak1* led cell death in early embryos^44^. It must be borne in mind that previous studies in *Drosophila* and silkworm demonstrated that during the developmental stages and metamorphosis further to pathogenic infections 20-hydroxyecdysone (20E), one of the two most important developmental hormones, improves the insect innate immune response, via upregulation of the PGRP-LCs^45,46^. Accordingly, our findings also support the solid interactions of developmental and immune system compartments.

*TmTak1* is required for activation of both Imd and JNK NF-κB after three type of pathogen such as Gram negative-, Gram positive-bacteria, and Fungi infection (Fig. 2). In *Drosophila*, PGN stimulation triggered TAK1 activation in both NF-κB and JNK mitogen-activated protein kinase (MAPK) signaling^25,37,47,48^. In *Drosophila* infected with Gram-negative bacteria, negative regulation of JNK signaling was demonstrated to be associated with proteasomal degradation of TAK1^36^. In crustacean, TAK1 activates the Imd and JNK pathways in response to Gram negative infections^49^. The endogenous TAK1 is phosphorylated in LPS-stimulated RAW 264.7 cells^50^. In mammalian, TAK1 is critical mediator of the antifungal immune response and a key component of the signaling cascade downstream of the c-type lectin receptor^51^.

The pathogen-derived metabolite, phenazine-1-carboxamide, has recently been discovered to be recognized by nuclear hormone receptors and to induce anti-pathogen defense in *C. elegans*^52^. Because a live microorganism was employed in this study, it is possible that the bacterial metabolite activates a non-canonical immunological pathway. Moreover, it has be reported repeatedly that various bacterial metabolite compositions which differ in distinct bacterial species including but not limited to *E. coli* might sequentially and cooperatively interfere with humoral and cellular defense response^53–55^. While these secondary metabolites might highly effect immune related signaling pathways via induction of host immunosuppression little is known about the relevant interaction. Hence, more research is needed to clarify the effect of bacterial metabolites on the immune system of *T. molitor*.

In *T. molitor*, TAK1 has been shown to be involved in the regulation of immune-related processes. For example, TAK1 activation has been shown to induce the expression of AMPs in *T. molitor* larvae. After infection with bacterial and fungal pathogens, the highest expression levels of *TmTak1* were observed specifically in response to *C. albicans* infection in multiple tissues including the gut, fat bodies, Malpighian tubules, integument, and hemocytes (Fig. 2). In mosquito, two entomopathogenic fungi *Beauveria bassiana* and *Isaria javanica* trigger Imd pathway component and induce AMPs in the gut and fat bodies^56^. In mice, fungal infection induce JNK1 which negatively controls innate immune response by suppressing CD23 expression^57^.

Like *Drosophila*, AMP production is regulated by Dorsal and Relish, which are transcription factors of Toll and Imd signaling pathways^58–60^. Moreover, *d*TAK1 is a factor involved in the JNK pathway^36^. JNK pathway is one of the main conserved signaling branch of the MAPK signaling pathway^61,62^. Kayak is one of the components of AP-1 transcription factor complex^63^. The sequences of *TmKayak* were retrieved from the *T. molitor* RNAseq and expressed sequence tag (EST) databases using local-tblastn analysis (unpublished). After gene silencing of *TmTak1*, the mRNA expression pattern of AMPs was significantly decreased by pathogen infection, proving that *Tm*TAK1 plays a critical role in AMP production. In order to confirm which transcription factors caused the absence of AMP expression due to gene silencing of *TmTak1*, the expression levels of Toll, Imd, and JNK former factors were examined. Despite systemic infection with *E. coli* and *S. aureus, TmRel* and *TmKay* were significantly decreased after *TmTak1* silencing in the MTs proposing critical roles of *Tm*Rel and *TmKay* in AMP induction. According to our finding, Gram-negative bacteria infection in shrimp revealed that TAK1 activated AMP production via c-jun and Relish^49^.

Knockdown of *TmTak1* using RNA interference resulted in increased mortality in *T. molitor* larvae following *E. coli* infection (Fig. 3B). TAK1 activates downstream kinase the inhibitor of NF-κB (IκB) kinase (IKK) complex^64^. Several studies have been conducted on immunological roles of IKK complex components^65–67^. For instance it has been shown that similar to *TmTak1*, knock-down of *TmIKKε* causes higher mortality rate post *E. coli* systemic infection^67^. Moreover, *TmIKKβ* and *TmIKKγ/NEMO* are critical immune factor for *T. molitor* survival following *E. coli*, *S. aureus* and *C. albicans* infections^65,66^.

Our observations bring out the importance of reaching a better perspective over *Tm*TAK1 regulatory acts on each IKK components independently and IKKs complex. In addition, the direct action of *Tm*TAK1 on components of the JNK pathway remains to be elucidated. This study suggests that *Tm*TAK1 plays a role in the innate immune response of *T. molitor* to Gram negative infection and also triggers Imd and JNK pathway.

## Materials and method

### Insect rearing

Insects were reared in a plastic container measuring 29 cm in width, 21 cm in length, and 9.5 cm in height, and wheat bran was used as food. The mealworms used in the experiment were separately fed to an artificial diet (170 g wheat flour, 20 g roasted soy flour, 10 g protein, 100 g wheat bran, 0.5 g sorbic acid, 0.5 mL propionic acid, and 0.5 g chloramphenicol in 200 mL of distilled water) and sterilized water was supplied once a day to maintain the temperature of 26 °C ± 1 °C and humidity of 60% ± 5% in an incubator. The larvae used in all experiments except for the developmental experiment were 10–12 years old (1.34–1.88  cm), and the adult was 5 days old after eclosion.

### Identification and in silico characterization

The sequences of *TmTak1* were retrieved from the *T. molitor* RNAseq (unpublished) and expressed sequence tag (EST) databases using local-tblastn analysis. For the retrieval, the amino acid sequences of *Tribolium castanuem* TAK1 (*Tc*TAK1) (XP_968547. 1) were used as a query. For *TmTak1* gene architecture, local-tblastn analysis was performed with *TmTak1* nucleotide sequence as query against the *T. molitor* DNASeq database (unpublished) as the subject. Conserved domains were identified using InterProScan (https://www.ebi.ac.uk/interpro/search/sequence-search) and blastp (https://blast.ncbi.nlm.nih.gov/Blast.cgi) programs. cDNA translation and predictions of the deduced protein were analyzed using BioPHP minitools software (http://www.biophp.org/minitools/dna_to_protein/demo.php). FGENESH eukaryotic gene prediction was used to predict *TmTak1* open reading frame (ORF) region (http://www.softberry.com/berry.phtml?topic=fgenesh&group=programs&subgroup=gfind). The complete cDNA sequence was formatted using UltraEdit64-bit text editor (http://www.ultraedit.com). For the reverse complement of the nucleotide sequence, ExPASy Translate tool was used (https://web.expasy.org/translate/). Multiple sequence alignment was performed with representative TAK1 protein sequences from other insects retrieved from Genbank (https://www.ncbi.nlm.nih.gov/) using Clustal X2^68^. The percentage identity and phylogenetic analysis were conducted using Clustal X2 and MEGA 7^69^. The Maximum likelihood approach was used to investigate phylogenetic relationships using the Jones–Taylor–Thorton model, with a bootstrap consensus of 1000 replications. The following protein sequences were used in the multiple sequence alignment and phylogenetic analysis: *Ie*MKKK7-like-isoX2, *Ischnura elegans* MKKK7-like-isoX2 (XP_046405324. 1); *Ie*MKKK7-like-isoX1, *Ischnura elegans* MKKK7-like-isoX1 (XP_046405323. 1); *Cs*MKKK7-isoX2, *Cryptotermes secundus* MKKK7-isoX2 (XP_023703195. 1); *Zn*MKKK7-isoX1, *Zootermopsis nevadensis* (XP_021932762. 1); *Dv*MKKK7-like, *Daktulosphaira virifoliae* MKKK7-like (XP_050524364. 1); *Sf*MKKK7-like, *Sipha flava* MKKK7-like (XP_025413188. 1); *Ac*MKKK7-like, *Aphis craccivora* MKKK7-like (KAF0770127. 1); *Fv*MKKK7-like, *Frieseomelitta varia* MKKK7-like (XP_043516360. 1); *Vc*MKKK7-like, *Venturia canescens* MKKK7-like (XP_043281270. 1); *Vc*MKKK7-like, *Venturia canescens* MKKK7-like (XP_043281270. 1); *Bk*MKKK7-like, *Belonocnema kinseyi* MKKK7-like (XP_033208032. 1); *Ar*MKKK7-like, *Athalia rosae* MKKK7-like (XP_012253187. 2); *Tm*TAK1, *Tenebrio molitor* TAK1 (OR_373077); *Tc*MKKK7, *Tribolium castaneum* MKKK7 (XP_968547. 1); *Pp*MKK7-like, *Photinus pyralis* MKKK7-like (XP_031330485. 1); *Ap*MKKK7, *Agrilus planipennis* MKKK7 (XP_018333073. 1); *Hk*MKK7-like, *Hyposmocoma kahamanoa* MKKK7-like (XP_026321167. 1); *Bm*MKKK7, *Bombyx mori* MKKK7 (XP_021207294. 1); *Hz*MKKK7, *Helicoverpa zea* MKKK7 (XP_047031840. 1); *Ha*MKKK7, *Helicoverpa armigera* MKKK7 (XP_021198433. 2); *Dm*TAK1-like1, *Drosophila melanogaster* TAK1-like1 (NP_732554. 1); *Dm*TAK1, *Drosophila melanogaster* TAK1 (NP_524080. 1); *Dm*TAK1-like2, *Drosophila melanogaster* TAK1-like2 (NP_651090. 2); *Pv*MKKK7-like, *Penaeus vannamei* MKKK7-like (XP_027228781. 1).

### Preparation of microorganisms

To investigate the immune function of *Tm*TAK1 against infection, we used three microorganisms including *Escherichia coli* K12, *Staphylococcus aureus* RN4220 and *Candida albicans* AUMC 13,529. *E. coil* and *S. aureus* were cultured in Luria–Bertani (LB) broth and *C. albicans* in Sabouraud Dextrose (SD) broth at 37 °C for 16 h. Then, the microorganisms were suspended by centrifugation at 3500 rpm for 10 min, washed with phosphate buffered saline (PBS, pH 7.0), and the concentration was measured at OD600. Finally, 1 × 10^6^ cells/μl of *E. coli* and *S. aureus* and 5 × 10^4^ cells/μl of *C. albicans* were used in all infection experiment, and only in the survivability assay, they were used in increments of 10 times more units.

### Total RNA extraction, cDNA synthesis and expression of *TmTak1* mRNA

RNA extraction, cDNA synthesis, and quantitative real-time PCR (qPCR) were commonly performed to investigate the mRNA expression level of *TmTak1* in developmental stages (n = 20) of *T. molitor* and each tissue (n = 20) and against microorganism. Total RNA extraction was performed according to the manual of RNA extraction kit (Invirustech, Gwangju, South Korea). The extracted RNA was quantified to 1 μg and synthesized into cDNA using AccuPower® RT PreMix (Bioneer, Daejeon, South Korea) and Oligo (dT)12–18 primer on a MyGenie 96 thermal block (Bioneer). Then, to investigated the mRNA expression level of *TmTak1*, AccuPower® 2X GreenStar™ qPCR Master Mix (Bioneer), synthesized cDNA and specific primers (*Tm*TAK1_qPCR_Fw, *Tm*TAK1_qPCR_Rv, Supplementary Table 1) were used for qPCR was performed under conditions of initial denaturation of 95 °C for 5 min, followed by 40 cycle at 95 °C for 15 s, and 60 °C for 30 s. *T. molitor* ribosomal protein (TmL27a) was used as a reference gene and the results were analyzed using the 2^−ΔΔCq^ method^70,71^.

### *TmTak1* gene silencing

To investigate the survival rate of *T. molitor* larvae, RNA interference (RNAi) was first performed using double-stranded RNA (dsRNA) of *TmTak1*. The ds*TmTak1* primer was designed using SnapDragon software (https://www.flyrnai.org/snapdragon) and synthesized by Bionics (Seoul, South Korea). PCR was performed using AccuPower® Pfu PCR PreMix & Master Mix (Bioneer), initial-denaturation of 94 °C for 5 min, followed by 39 cycles of denaturation at 94 °C for 30 s, annealing at 54 °C for 30 s, extension at 72 °C for 1 min, and final extension at 72 °C for 5 min was performed under conditions. The PCR product was purified according to the manual of the Clear-STM PCR/Gel DNA fragment purification kit (Invirustech), and dsRNA was prepared according to the manual of the EZTM T7 high yield in vitro transcription kit (Enzynomics, Daejeon, South Korea). The dsRNA product was purified using 5 M ammonium acetate and 99% ethanol and was finally quantified to 1 μg/μl and stored at − 80 °C. As a negative control, ds*EGFP* was used, and injection was performed by quantifying at 1 μg/μl. After the injected samples were sampled, RNA was extracted and used for RT-qPCR, reverse-transcription step at 50 °C for 10 min, initial denaturation step at 95 °C for 5 min, followed by 39 cycles at 95 °C for 15 s, and 60 °C for 30 s.

### Effects of *TmTak1* RNAi on the expression mRNA pattern of AMP and NF-κB genes

To evaluate the functional properties of *TmTak1* in regulating AMP gene expression in response to pathogens, we used ds*TmTak1* to RNAi and then injected *E. coli*, *S. aureus*, and *C. albicans* into larvae. ds*EGFP* and PBS served as negative and injection controls, respectively. Twenty-four hours after injection, whole body (WB) and five tissues which are Malpighian tubules (MTs), gut (GT), Integument (INT), fat bodies (FBs), Hemocytes (HCs) were dissected, total RNA was extracted from each tissue (n = 20), and cDNA was synthesized as described above. Next, by performing qPCR with specific primers, 15 AMP genes: *TmTenecin-1*, *-2*, -*3*, *-4* (*TmTene1*, *2*, *3*, *4*), *TmAttacin-1a*, *-1b*, *-2* (*TmAtta1a*, *1b*, *2*), *TmDefensin* (*TmDef*), *TmDefensin-like* (*TmDef-like*), *TmColeoptericin-A*, *-B*, *-C* (*TmColeA*, *B*, *C*), *TmCecropin-2* (*TmCec2*) and *TmThaumatin like protein-1*, *-2* (*TmTLP1, 2*). To investigate the effect of ds*TmTak1* on the expression of NF-κB genes, including *TmRelish* (*TmRel*), *TmKayak* (*TmKay*) and *TmDorsal isoform -X1, -X2* (*TmDorX1, X2*) bacteria were injected, dissected, and qPCR was performed after RNAi as in the method for examining AMP expression.

### Statistical analysis

All experiments were carried out in triplicate. These data were subjected to *t* test or a one-way analysis of variance (ANOVA). Tukey’s multiple range tests were used to evaluate the difference between groups (*p* < 0.05).

### Supplementary Information

Supplementary Information.

## Data Availability

The datasets generated and analyzed using BLASTp during the current study are available in the (NCBI; https://blast.ncbi.nlm.nih.gov/Blast.cgi) repository, (accession number: OR373077).

## References

[1] Hong J, Han T, Kim YY (2020). Mealworm (*Tenebrio molitor* Larvae) as an alternative protein source for monogastric animal: A review. Animals (Basel).

[2] Fraenkel G, Blewett M, Coles M (1950). The nutrition of the mealworm, tenebrio molitor L (tenebrionidae, coleoptera). Physiol. Zool..

[3] Vigneron A, Jehan C, Rigaud T, Moret Y (2019). Immune defenses of a beneficial pest: The mealworm beetle, *Tenebrio molitor*. Front. Physiol..

[4] Ali Mohammadie Kojour M (2022). Current knowledge of immune priming in invertebrates, emphasizing studies on *Tenebrio molitor*. Dev. Comp. Immunol..

[5] Jang HA, Kojour MAM, Patnaik BB, Han YS, Jo YH (2022). Current status of immune deficiency pathway in *Tenebrio molitor* innate immunity. Front. Immunol..

[6] Savio C, Mugo-Kamiri L, Upfold JK (2022). Bugs in bugs: The role of probiotics and prebiotics in maintenance of health in mass-reared insects. Insects.

[7] Grau T, Vilcinskas A, Joop G (2017). Sustainable farming of the mealworm *Tenebrio molitor* for the production of food and feed. Z. Naturforsch C J. Biosci..

[8] Maistrou S, Paris V, Jensen AB, Rolff J, Meyling NV, Zanchi C (2018). A constitutively expressed antifungal peptide protects *Tenebrio molitor* during a natural infection by the entomopathogenic fungus *Beauveria bassiana*. Dev. Comp. Immunol..

[9] Jang HA (2020). In silico identification and expression analyses of Defensin genes in the mealworm beetle *Tenebrio molitor*. Entomol. Res..

[10] Ali Mohammadie Kojour M (2021). Identification, in silico characterization, and expression analysis of *Tenebrio molitor* Cecropin-2. Entomol. Res..

[11] Jo YH (2018). In silico identification, characterization and expression analysis of attacin gene family in response to bacterial and fungal pathogens in *Tenebrio molitor*. Entomol. Res..

[12] Chae J-H (2012). Purification and characterization of tenecin 4, a new anti-Gram-negative bacterial peptide, from the beetle *Tenebrio molitor*. Dev. Comp. Immunol..

[13] Hoffmann JA, Kafatos FC, Janeway CA, Ezekowitz R (1999). Phylogenetic perspectives in innate immunity. Science.

[14] Akira S, Uematsu S, Takeuchi O (2006). Pathogen recognition and innate immunity. Cell.

[15] Hoffmann JA (2003). The immune response of Drosophila. Nature.

[16] Hillyer JF (2016). Insect immunology and hematopoiesis. Dev. Comp. Immunol..

[17] Salcedo-Porras N, Lowenberger C (2019). The innate immune system of kissing bugs, vectors of chagas disease. Dev. Comp. Immunol..

[18] Lemaitre B, Hoffmann J (2007). The host defense of *Drosophila melanogaster*. Annu. Rev. Immunol..

[19] Levashina EA, Ohresser S, Lemaitre B, Imler JL (1998). Two distinct pathways can control expression of the gene encoding the Drosophila antimicrobial peptide metchnikowin. J. Mol. Biol..

[20] Delaney JR, Stöven S, Uvell H, Anderson KV, Engström Y, Mlodzik M (2006). Cooperative control of Drosophila immune responses by the JNK and NF-kappaB signaling pathways. Embo J..

[21] Kallio J, Leinonen A, Ulvila J, Valanne S, Ezekowitz RA, Rämet M (2005). Functional analysis of immune response genes in Drosophila identifies JNK pathway as a regulator of antimicrobial peptide gene expression in S2 cells. Microbes Infect..

[22] Kurata S (2014). Peptidoglycan recognition proteins in Drosophila immunity. Dev. Comp. Immunol..

[23] Michel T, Reichhart J-M, Hoffmann JA, Royet J (2001). Drosophila Toll is activated by Gram-positive bacteria through a circulating peptidoglycan recognition protein. Nature.

[24] Neyen C, Poidevin M, Roussel A, Lemaitre B (2012). Tissue-and ligand-specific sensing of gram-negative infection in drosophila by PGRP-LC isoforms and PGRP-LE. J. Immunol..

[25] Vidal S, Khush RS, Leulier F, Tzou P, Nakamura M, Lemaitre B (2001). Mutations in the Drosophila dTAK1 gene reveal a conserved function for MAPKKKs in the control of rel/NF-kappaB-dependent innate immune responses. Genes Dev..

[26] Sheehan G, Garvey A, Croke M, Kavanagh K (2018). Innate humoral immune defences in mammals and insects: The same, with differences?. Virulence.

[27] Aggarwal BB (2003). Signalling pathways of the TNF superfamily: A double-edged sword. Nat. Rev. Immunol..

[28] Broglie P, Matsumoto K, Akira S, Brautigan DL, Ninomiya-Tsuji J (2010). Transforming growth factor beta-activated kinase 1 (TAK1) kinase adaptor, TAK1-binding protein 2, plays dual roles in TAK1 signaling by recruiting both an activator and an inhibitor of TAK1 kinase in tumor necrosis factor signaling pathway. J. Biol. Chem..

[29] Kajino T (2006). Protein phosphatase 6 down-regulates TAK1 kinase activation in the IL-1 signaling pathway. J. Biol. Chem..

[30] Takaesu G, Ninomiya-Tsuji J, Kishida S, Li X, Stark GR, Matsumoto K (2001). Interleukin-1 (IL-1) receptor-associated kinase leads to activation of TAK1 by inducing TAB2 translocation in the IL-1 signaling pathway. Mol. Cell Biol..

[31] Yamaguchi K (1995). Identification of a member of the MAPKKK family as a potential mediator of TGF-β signal transduction. Science.

[32] Irie T, Muta T, Takeshige K (2000). TAK1 mediates an activation signal from toll-like receptor(s) to nuclear factor-kappaB in lipopolysaccharide-stimulated macrophages. FEBS Lett..

[33] Blonska M (2005). TAK1 is recruited to the tumor necrosis factor-alpha (TNF-alpha) receptor 1 complex in a receptor-interacting protein (RIP)-dependent manner and cooperates with MEKK3 leading to NF-kappaB activation. J. Biol. Chem..

[34] Kleino A, Silverman N (2014). The Drosophila IMD pathway in the activation of the humoral immune response. Dev. Comp. Immunol..

[35] Ali Mohammadie Kojour M, Han YS, Jo YH (2020). An overview of insect innate immunity. Entomol. Res..

[36] Park JM (2004). Targeting of TAK1 by the NF-kappa B protein Relish regulates the JNK-mediated immune response in Drosophila. Genes Dev..

[37] Silverman N (2003). Immune activation of NF-kappaB and JNK requires Drosophila TAK1. J. Biol. Chem..

[38] Tsapras P (2022). Selective autophagy controls innate immune response through a TAK1/TAB2/SH3PX1 axis. Cell Rep..

[39] Xia ZP (2009). Direct activation of protein kinases by unanchored polyubiquitin chains. Nature.

[40] Xu M, Skaug B, Zeng W, Chen ZJ (2009). A ubiquitin replacement strategy in human cells reveals distinct mechanisms of IKK activation by TNFalpha and IL-1beta. Mol. Cell.

[41] Paquette N (2012). Serine/threonine acetylation of TGFβ-activated kinase (TAK1) by Yersinia pestis YopJ inhibits innate immune signaling. Proc. Natl. Acad. Sci. U S A.

[42] Roh YS, Song J, Seki E (2014). TAK1 regulates hepatic cell survival and carcinogenesis. J. Gastroenterol..

[43] Sakurai H, Miyoshi H, Mizukami J, Sugita T (2000). Phosphorylation-dependent activation of TAK1 mitogen-activated protein kinase kinase kinase by TAB1. FEBS Lett..

[44] Shibuya H (1998). Role of TAK1 and TAB1 in BMP signaling in early Xenopus development. Embo J..

[45] Sun W, Shen YH, Zhou LX, Zhang Z (2016). Ecdysone titer determined by 3DE-3β-reductase enhances the immune response in the silkworm. J. Immunol..

[46] Rus F (2013). Ecdysone triggered PGRP-LC expression controls Drosophila innate immunity. Embo J..

[47] Boutros M, Agaisse H, Perrimon N (2002). Sequential activation of signaling pathways during innate immune responses in Drosophila. Dev. Cell.

[48] Chen W, White MA, Cobb MH (2002). Stimulus-specific requirements for MAP3 kinases in activating the JNK pathway. J. Biol. Chem..

[49] Wang S, Li H, Chen R, Jiang X, He J, Li C (2022). TAK1 confers antibacterial protection through mediating the activation of MAPK and NF-κB pathways in shrimp. Fish Shellfish Immunol..

[50] Irie T, Muta T, Takeshige K (2000). TAK1 mediates an activation signal from toll-like receptor (s) to nuclear factor-κB in lipopolysaccharide-stimulated macrophages. FEBS Lett..

[51] Gorjestani S, Darnay BG, Lin X (2012). Tumor necrosis factor receptor-associated factor 6 (TRAF6) and TGFβ-activated kinase 1 (TAK1) play essential roles in the C-type lectin receptor signaling in response to *Candida albicans* infection. J. Biol. Chem..

[52] Peterson ND, Tse SY, Huang QJ, Wani KA, Schiffer CA, Pukkila-Worley R (2023). Non-canonical pattern recognition of a pathogen-derived metabolite by a nuclear hormone receptor identifies virulent bacteria in *C. elegans*. Immunity.

[53] Eom S, Park Y, Kim Y (2014). Sequential immunosuppressive activities of bacterial secondary metabolites from the entomopahogenic bacterium *Xenorhabdus nematophila*. J. Microbiol..

[54] Kim MH (2000). Bacterial-injection-induced syntheses of N-beta-alanyldopamine and Dopa decarboxylase in the hemolymph of coleopteran insect, *Tenebrio molitor* larvae. Eur. J. Biochem..

[55] Mollah MMI, Kim Y (2020). Virulent secondary metabolites of entomopathogenic bacteria genera, Xenorhabdus and Photorhabdus, inhibit phospholipase A(2) to suppress host insect immunity. BMC Microbiol..

[56] Ramirez JL, Muturi EJ, Barletta ABF, Rooney AP (2019). The *Aedes aegypti* IMD pathway is a critical component of the mosquito antifungal immune response. Dev. Comp. Immunol..

[57] Zhao X (2017). JNK1 negatively controls antifungal innate immunity by suppressing CD23 expression. Nat. Med..

[58] Hetru, C. & Hoffmann, J. (2009).

[59] Keshavarz M (2019). *TmDorX2* positively regulates antimicrobial peptides in *Tenebrio molitor* gut, fat body, and hemocytes in response to bacterial and fungal infection. Sci. Rep..

[60] Keshavarz M (2020). *Tm*Relish is required for regulating the antimicrobial responses to *Escherichia coli* and *Staphylococcus aureus* in *Tenebrio molitor*. Sci. Rep..

[61] Pearson G (2001). Mitogen-activated protein (MAP) kinase pathways: Regulation and physiological functions. Endocr. Rev..

[62] La Marca JE, Richardson HE (2020). Two-faced: Roles of JNK signalling during tumourigenesis in the Drosophila model. Front. Cell Dev. Biol..

[63] Igaki T (2009). Correcting developmental errors by apoptosis: Lessons from Drosophila JNK signaling. Apoptosis.

[64] Cammarata-Mouchtouris A, Acker A, Goto A, Chen D, Matt N, Leclerc V (2022). Dynamic regulation of NF-κB response in innate immunity: The case of the IMD pathway in Drosophila. Biomedicines.

[65] Ko HJ (2023). IKKβ regulates antimicrobial innate immune responses in the yellow mealworm, *Tenebrio molitor*. Dev. Comp. Immunol..

[66] Ko HJ (2020). IKKγ/NEMO is required to confer antimicrobial innate immune responses in the yellow mealworm, *Tenebrio molitor*. Int. J. Mol. Sci..

[67] Ko HJ (2022). Tm IKKε is required to confer protection against gram-negative bacteria, *E. coli* by the regulation of antimicrobial peptide production in the *Tenebrio molitor* fat body. Front. Physiol..

[68] Larkin MA (2007). Clustal W and clustal X version 2.0. Bioinformatics.

[69] Kumar S, Stecher G, Tamura K (2016). MEGA7: Molecular evolutionary genetics analysis version 7.0 for bigger datasets. Mol. Biol. Evol..

[70] Livak KJ, Schmittgen TD (2001). Analysis of relative gene expression data using real-time quantitative PCR and the 2(-Delta Delta C(T)) Method. Methods.

[71] Carmona R (2017). Automated identification of reference genes based on RNA-seq data. Biomed. Eng. Online.

