# Supplementary material for: Effects of TmTak1 silencing on AMP production as an Imd pathway component in Tenebrio molitor
Source: Sci Rep. 2023 Nov 2;13:18914. doi: 10.1038/s41598-023-45978-4 (PMC10622451; doi:10.1038/s41598-023-45978-4)
Supplement: Supplementary file 1 — Supplementary Information. [file 41598_2023_45978_MOESM1_ESM.docx]

**Supplementary Figure 1.**

The coding sequence of *TmTak1* includes 1,527 bp of nucleotide sequences encompassing eight exons (shown in blue). The intervening sequences (introns) have been shown in grey. Linear map of *TmTak1* gene structure. The cDNA sequence of *TmTak1* annotated against the *T. molitor* DNA-seq database.


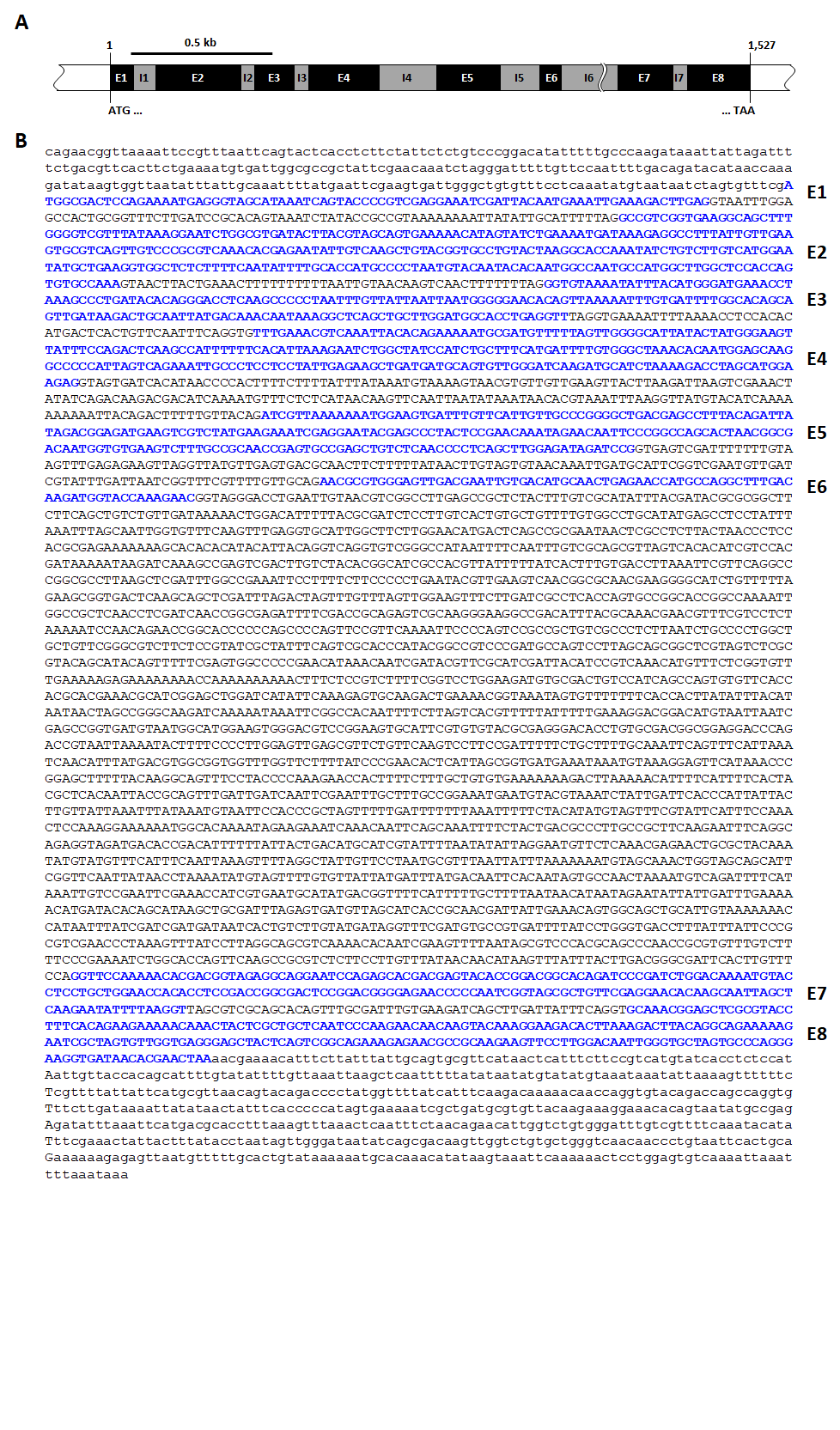


**Supplementary Figure 2. Nucleotide and deduced amino acid sequence of *Tm*Tak1.** *Tm*Tak1 contains 1,527 bp of nucleotide sequences encoding 703 amino acid residues. The nucleotide and amino acid residues are numbered. The stop codon is represented by an asterisk. The Serine/Threonine protein kinase catalytic domain is represented within the Open box.


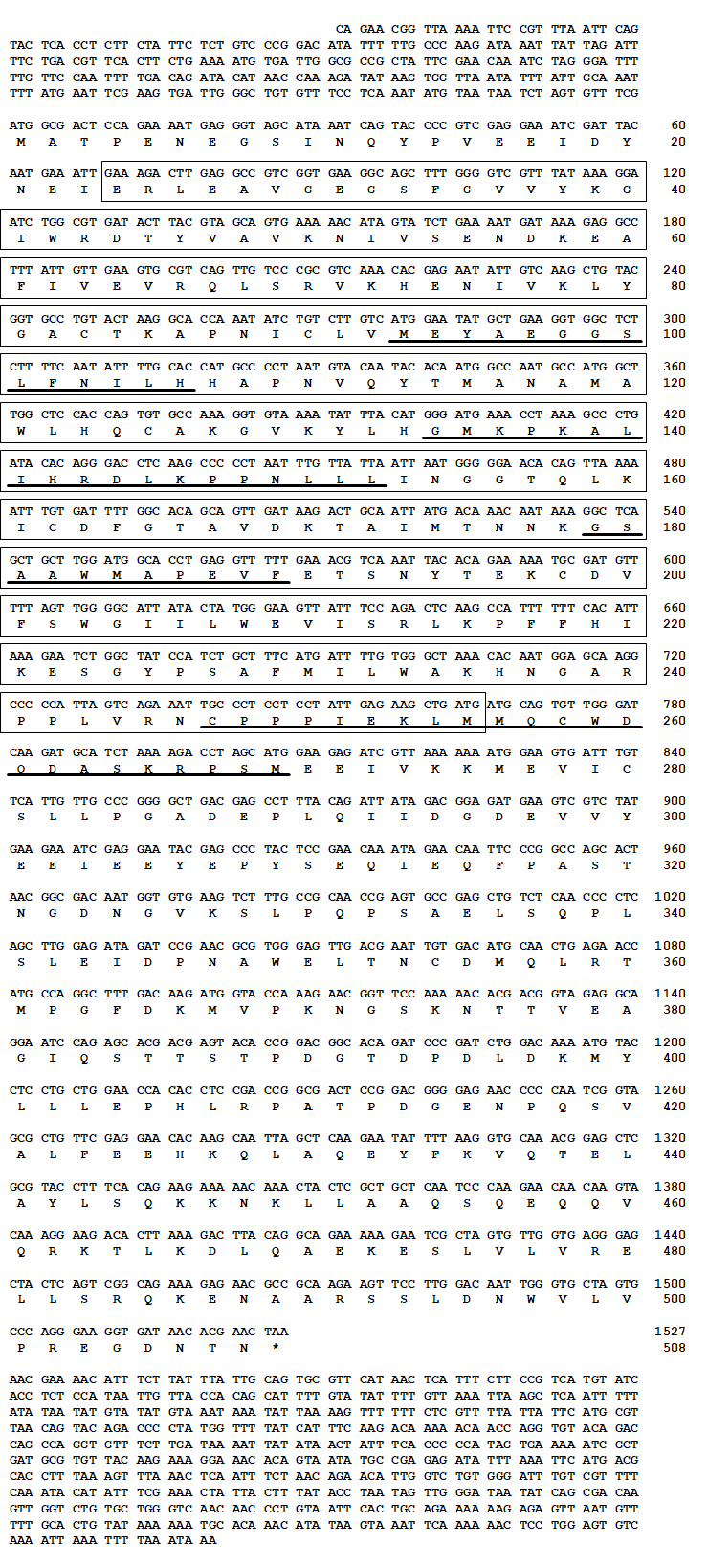


**Supplementary Figure 3.** **Multiple alignment analysis of *Tm*TAK1.** Multiple alignments of the deduced amino acid sequence of TmTAK1 aligned using Clustal X 2.0.11. ArRaws represent the phylogenetically conserved cysteine residues forming the disulfide bridge featuring in the cysteine knot domain.

The following protein sequences were used in the multiple sequence alignment and phylogenetic analysis: *Dm*TAK1, *Drosophila melanogaster* TAK1 (NP_524080. 1); *Dm*TAK1-like2, *Drosophila melanogaster* TAK1-like2 (NP_651090. 2); *Tm*TAK1, *Tenebrio molitor* TAK1 (OR_373077); *Tc*MKKK7, *Tribolium castaneum* MKKK7 (XP_968547. 1); *Pp*MKK7-like, *Photinus pyralis* MKKK7-like (XP_031330485. 1); *Ap*MKKK7, *Agrilus planipennis* MKKK7 (XP_018333073. 1); *Hz*MKKK7, *Helicoverpa zea* MKKK7 (XP_047031840. 1); *Ha*MKKK7, *Helicoverpa armigera* MKKK7 (XP_021198433. 2); *Bm*MKKK7, *Bombyx mori* MKKK7 (XP_021207294. 1); *Hk*MKK7-like, *Hyposmocoma kahamanoa* MKKK7-like (XP_026321167. 1); *Bk*MKKK7-like, *Belonocnema kinseyi* MKKK7-like (XP_033208032. 1); *Ar*MKKK7-like, *Athalia rosae* MKKK7-like (XP_012253187. 2); *Fv*MKKK7-like, *Frieseomelitta varia* MKKK7-like (XP_043516360. 1); *Vc*MKKK7-like, *Venturia canescens* MKKK7-like (XP_043281270. 1); *Sf*MKKK7-like, *Sipha flava* MKKK7-like (XP_025413188. 1); *Ac*MKKK7-like, *Aphis craccivora* MKKK7-like (KAF0770127. 1); *Dv*MKKK7-like, *Daktulosphaira virifoliae* MKKK7-like (XP_050524364. 1); *Ie*MKKK7-like-isoX2, *Ischnura elegans* MKKK7-like-isoX2 (XP_046405324. 1); *Ie*MKKK7-like-isoX1, *Ischnura elegans* MKKK7-like-isoX1 (XP_046405323. 1); *Cs*MKKK7-isoX2, *Cryptotermes secundus* MKKK7-isoX2 (XP_023703195. 1); *Zn*MKKK7-isoX1, *Zootermopsis nevadensis* (XP_021932762. 1); *Pv*MKKK7-like, *Penaeus vannamei* MKKK7-like (XP_027228781. 1); *Dm*TAK1-like1, *Drosophila melanogaster* TAK1-like1 (NP_732554. 1)

**
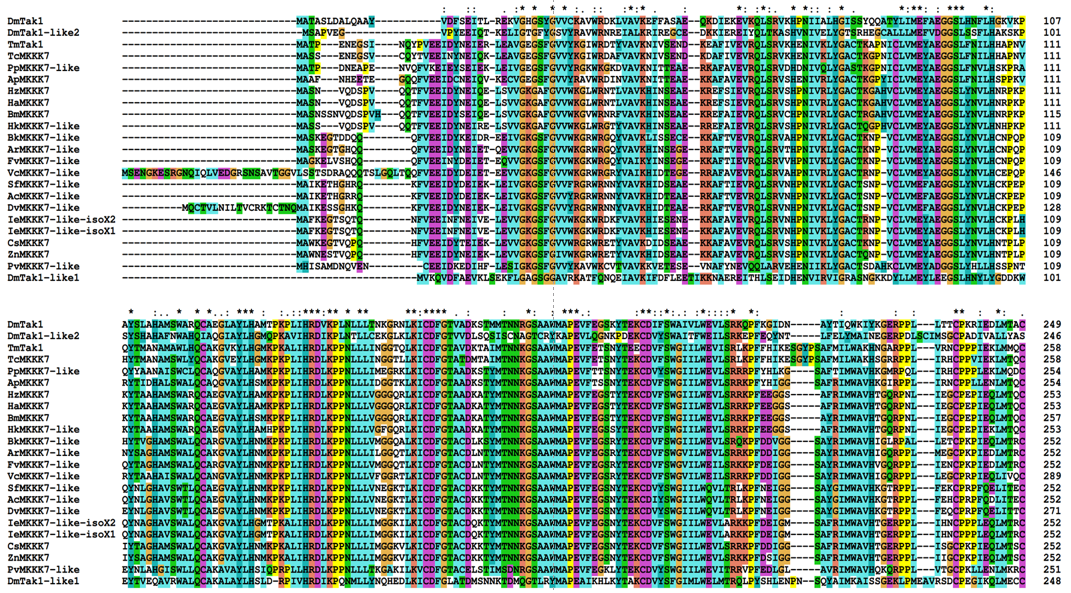
**

**
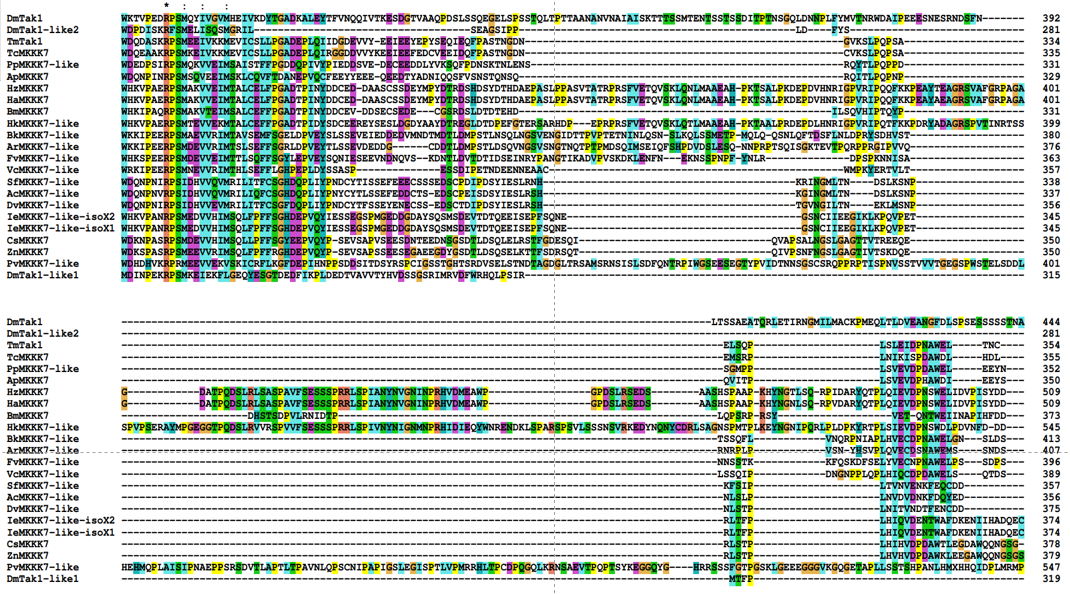
**

**
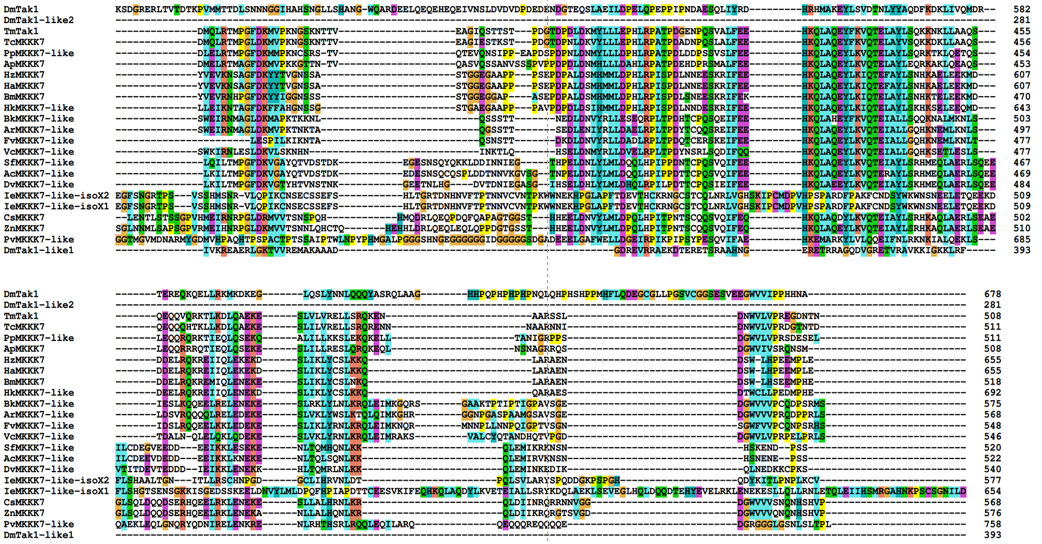
**

**Supplementary Figure 4.** **Molecular phylogenetic analysis of *Tm*TAK1.** Maximum likelihood-based phylogenetic tree based on the amino acid sequence of *Tm*TAK1 constructed using the MEGA 7.0 software. The following protein sequences were used in the multiple sequence alignment and phylogenetic analysis: *Ie*MKKK7-like-isoX2, *Ischnura elegans* MKKK7-like-isoX2 (XP_046405324. 1); *Ie*MKKK7-like-isoX1, *Ischnura elegans* MKKK7-like-isoX1 (XP_046405323. 1); *Cs*MKKK7-isoX2, *Cryptotermes secundus* MKKK7-isoX2 (XP_023703195. 1); *Zn*MKKK7-isoX1, *Zootermopsis nevadensis* (XP_021932762. 1); *Dv*MKKK7-like, *Daktulosphaira virifoliae* MKKK7-like (XP_050524364. 1); *Sf*MKKK7-like, *Sipha flava* MKKK7-like (XP_025413188. 1); *Ac*MKKK7-like, *Aphis craccivora* MKKK7-like (KAF0770127. 1); *Fv*MKKK7-like, *Frieseomelitta varia* MKKK7-like (XP_043516360. 1); *Vc*MKKK7-like, *Venturia canescens* MKKK7-like (XP_043281270. 1); *Vc*MKKK7-like, *Venturia canescens* MKKK7-like (XP_043281270. 1); *Bk*MKKK7-like, *Belonocnema kinseyi* MKKK7-like (XP_033208032. 1); *Ar*MKKK7-like, *Athalia rosae* MKKK7-like (XP_012253187. 2); *Tm*TAK1, *Tenebrio molitor* TAK1 (OR_373077); TcMKKK7, *Tribolium castaneum* MKKK7 (XP_968547. 1); *Pp*MKK7-like, *Photinus pyralis* MKKK7-like (XP_031330485. 1); *Ap*MKKK7, *Agrilus planipennis* MKKK7 (XP_018333073. 1); *Hk*MKK7-like, *Hyposmocoma kahamanoa* MKKK7-like (XP_026321167. 1); *Bm*MKKK7, *Bombyx mori* MKKK7 (XP_021207294. 1); *Hz*MKKK7, *Helicoverpa zea* MKKK7 (XP_047031840. 1); *Ha*MKKK7, *Helicoverpa armigera* MKKK7 (XP_021198433. 2); *Dm*TAK1-like1, *Drosophila melanogaster* TAK1-like1 (NP_732554. 1); *Dm*TAK1, *Drosophila melanogaster* TAK1 (NP_524080. 1); *Dm*TAK1-like2, *Drosophila melanogaster* TAK1-like2 (NP_651090. 2); *Pv*MKKK7-like, *Penaeus vannamei* MKKK7-like (XP_027228781. 1)


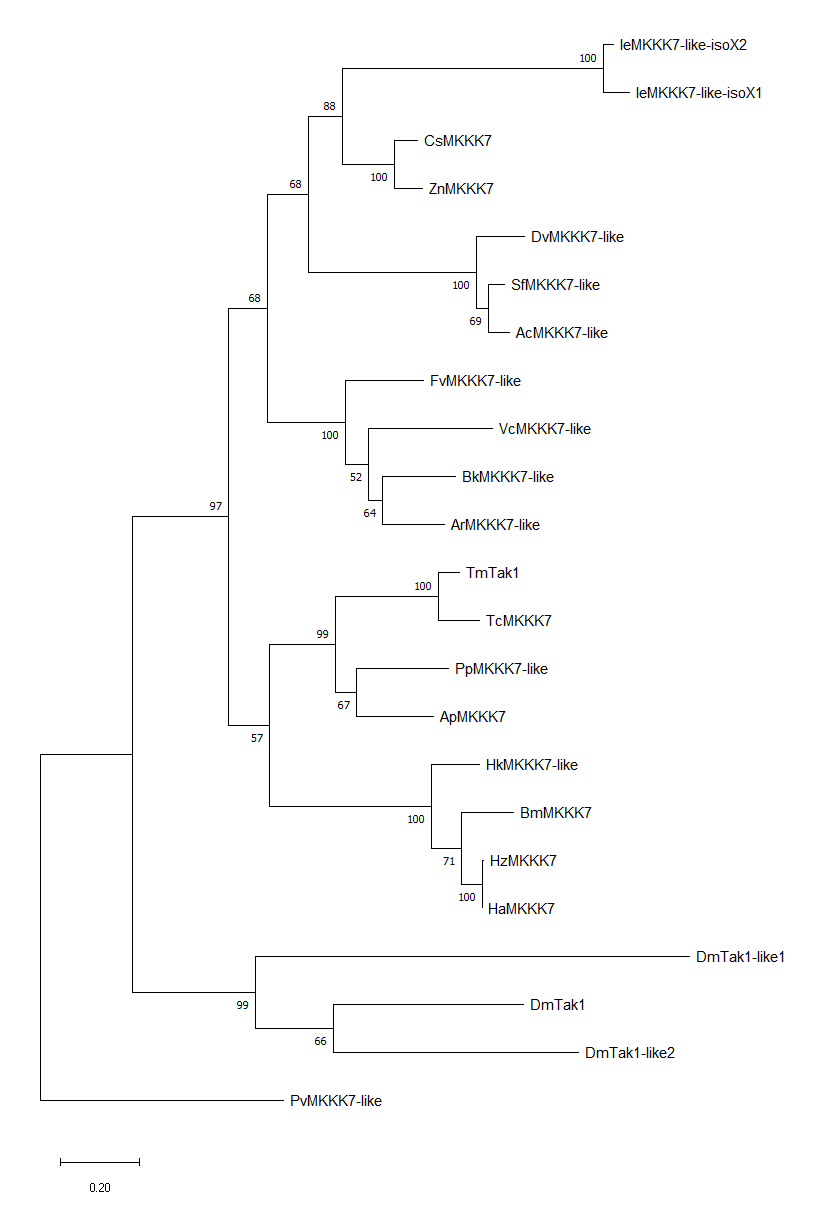


Odonata

Isoptera

Hemiptera

Coleoptera

Diptera

Hymenoptera

Lepidoptera

Decapoda

# Supplementary Figure 5. Effect of ds*TmTak1* and ds*EGFP* RNAi on *T. molitor* Larval Survival. The survivability of larva was measured after *TmTak1* knockdown and infection PBS (n = 30). Data are presented as average of three biologically independent replicate experiments.


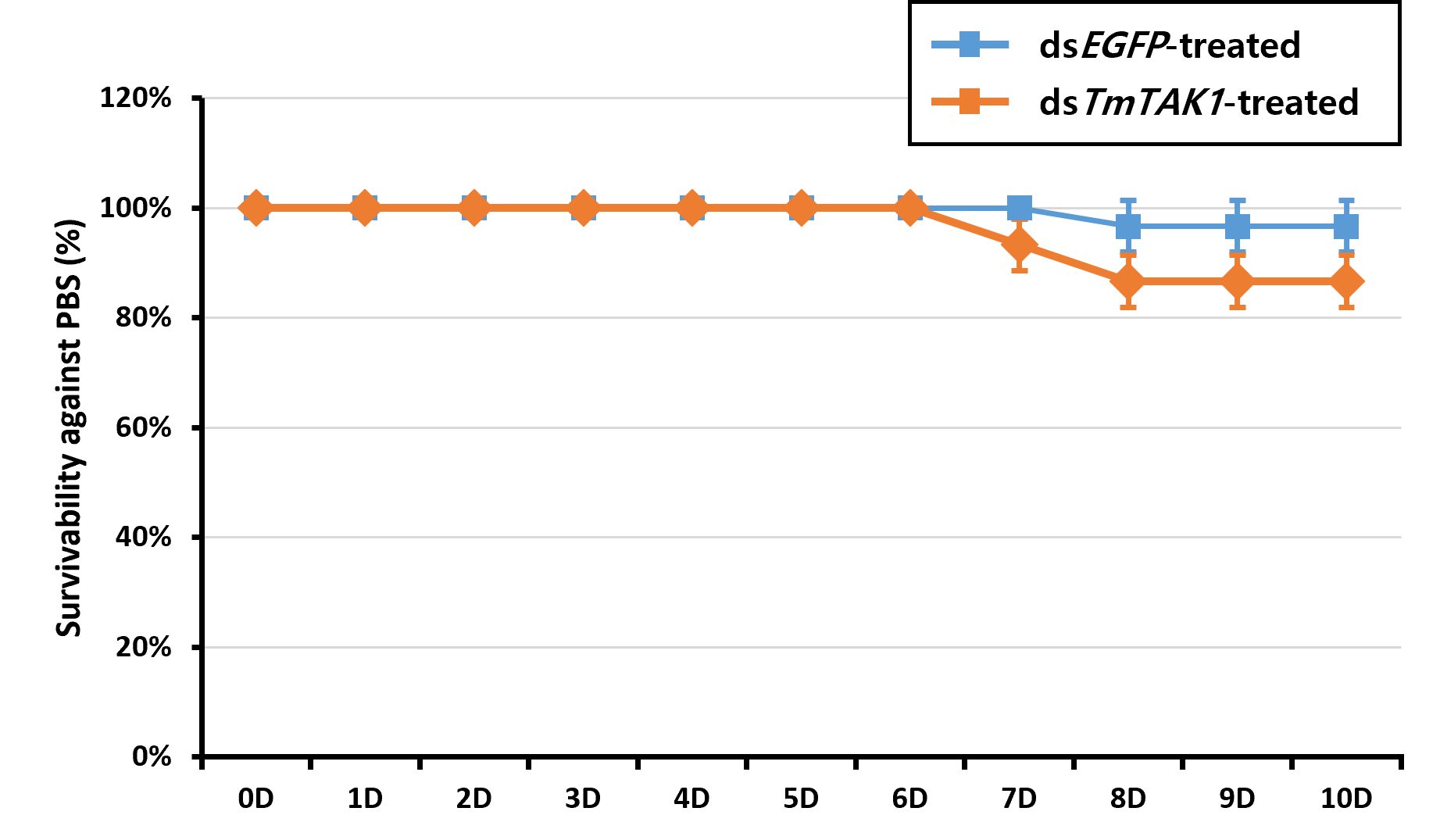


**Supplementary Table1.** **Primers used in the present study**

| **Name** | **Primer sequences** |
| --- | --- |
| *Tm*TAK1_cloning_Fw  *Tm*TAK1_cloning_Rv | 5′- TCGAAGTGATTGGGCTGTGT -3′  5′- TTATGGAGAGGTGATACATGACGG -3′ |
| *Tm*TAK1_qPCR_Fw  *Tm*TAK1_qPCR_Rv | 5′- TGTGAAGTCTTTGCCGCAAC -3′  5′- TCTTGTCAAAGCCTGGCATG -3′ |
| *Tm*TAK1_T7_Fw  *Tm*TAK1_T7_Rv | 5′-TAATACGACTCACTATAGGGT  AGTACCCCGTCGAGGAAATC -3′  5′-TAATACGACTCACTATAGGGT  GCATTGGCCATTGTGTATTG -3′ |
| *Tm*Tenecin-1_qPCR_Fw  *Tm*Tenecin-1_qPCR_Rv | 5′-CAGCTGAAGAAATCGAACAAGG-3′  5′-CAGACCCTCTTTCCGTTACAGT-3′ |
| *Tm*Tenecin-2_qPCR_Fw  *Tm*Tenecin-2_qPCR_Rv | 5′-CAGCAAAACGGAGGATGGTC-3′  5′-CGTTGAAATCGTGATCTTGTCC-3′ |
| *Tm*Tenecin-3_qPCR_Fw  *Tm*Tenecin-3_qPCR_Rv | 5′-GATTTGCTTGATTCTGGTGGTC-3′  5′-CTGATGGCCTCCTAAATGTCC-3′ |
| *Tm*Tenecin-4_qPCR_Fw  *Tm*Tenecin-4_qPCR_Rv | 5′-GGACATTGAAGATCCAGGAAAG-3′  5′-CGGTGTTCCTTATGTAGAGCTG-3′ |
| *Tm*Defensin_qPCR_Fw  *Tm*Defensin_qPCR_Rv | 5′-AAATCGAACAAGGCCAACAC-3′  5′-GCAAATGCAGACCCTCTTTC-3′ |
| *Tm*Defensin-like_qPCR_Fw  *Tm*Defensin-like_qPCR_Rv | 5′-GCGATGCCTCATGAAGATGTAG-3′  5′-CCAATGCAAACACATTCGTC-3′ |
| *Tm*Cecropin-2_qPCR_Fw  *Tm*Cecropin-2_qPCR_Rv | 5′-TACTAGCAGCGCCAAAACCT-3′  5′-CTGGAACATTAGGCGGAGAA-3′ |
| *Tm*Coleoptericin-A_qPCR_Fw  *Tm*Coleoptericin-A_qPCR_Rv | 5′-GGACAGAATGGTGGATGGTC-3′  5′-CTCCAACATTCCAGGTAGGC-3′ |
| *Tm*Coleoptericin-B_qPCR_Fw  *Tm*Coleoptericin-B_qPCR_Rv | 5′-CAGCTGTTGCCCACAAAGTG-3′  5′-CTCAACGTTGGTCCTGGTGT-3′ |
| *Tm*Coleoptericin-C-qPCR-Fw  *Tm*Coleoptericin-C-qPCR-Rv | 5'-GGACGGTTCTGATCTTCTTGAT-3'  5'CAGCTGTTTGTTTGTTCTCGTC-3' |
| *Tm*Attacin-1a_qPCR_Fw  *Tm*Attacin-1a_qPCR_Rv | 5′-AAAGTGGTCCCCACCGATTC-3′  5′-GCGCTGAATGTTTTCGGCTT-3′ |
| *Tm*Attacin-1b_qPCR_Fw  *Tm*Attacin-1b_qPCR_Rv | 5′-GAGCTGTGAATGCAGGACAA-3′  5′-CCCTCTGATGAAACCTCCAA-3′ |
| *Tm*Attacin-2-Fw  *Tm*Attacin-2-Rv | 5′-AACTGGGATATTCGCACGTC-3′  5′-CCCTCCGAAATGTCTGTTGT-3′ |
| *Tm*Thaumatin-like protein-1-Fw  *Tm*Thaumatin-like protein-1-Rv | 5′-CTCAAAGGACACGCAGGACT-3′  5′-ACTTTGAGCTTCTCGGGACA-3′ |
| *Tm*Thaumatin-like protein-2-Fw  *Tm*Thaumatin-like protein-2-Rv | 5′-CCGTCTGGCTAGGAGTTCTG-3′  5′-ACTCCTCCAGCTCCGTTACA-3′ |
| *Tm*Relish_qPCR_Fw  *Tm*Relish_qPCR_Rv | 5′-AGCGTCAAGTTGGAGCAGAT-3′  5′-GTCCGGACCTCAAGTGT-3′ |
| *Tm*kayak_qPCR_Fw  *Tm*kayak_qPCR_Rv | 5′-AAGGGAACGCAACAAAGCAG-3′  5′-AGCTCGTCGGTTTCTTGAAC-3′ |
| *Tm*Dorax1_qPCR_Fw  *Tm*Dorax1_qPCR_Rv | 5′-AGCGTTGAGGTTTCGGTATG-3′  5′-TCTTTGGTGACGCAAGACAC-3′ |
| *Tm*Dorax2_qPCR_Fw  *Tm*Dorax2_qPCR_Rv | 5′-ACACCCCCGAAATCACAAAC-3′  5′-TTTCAGAGCGCCAGGTTTTG-3′ |
| *Tm*L27a_qPCR_Fw  *Tm*L27a_qPCR_Rv | 5′-TCATCCTGAAGGCAAAGCTCCAGT-3′  5′-AGGTTGGTTAGGCAGGCACCTTTA-3′ |

※ Underline indicates T7 promotor sequences

**Supplementary Table2. Raw data of qPCR in developmental stages.**

Cq data of *TmTak1* and *L27a* in *T. molitor* at the egg, the young larval (YL) , late larval (LL), pre-pupal (PP), 1–7-day-old pupal (P1–7), and 1–5-day-old adult (A1–5) stages.

| Sample name | Gene name | Cq (∆R) | Sample name | Gene name | Cq (∆R) |
| --- | --- | --- | --- | --- | --- |
| Egg | *TmTak1* | 26.6 | P6 | *TmTak1* | 26.86 |
| Egg | *TmTak1* | 25.63 | P6 | *TmTak1* | 27.46 |
| Egg | *TmL27a* | 19.49 | P6 | *TmL27a* | 19.53 |
| Egg | *TmL27a* | 19.63 | P6 | *TmL27a* | 19.15 |
| YL | *TmTak1* | 22.72 | P7 | *TmTak1* | 27.34 |
| YL | *TmTak1* | 22.46 | P7 | *TmTak1* | 28.59 |
| YL | *TmL27a* | 16.59 | P7 | *TmL27a* | 18.05 |
| YL | *TmL27a* | 16.61 | P7 | *TmL27a* | 19.72 |
| LL | *TmTak1* | 26.85 | A1 | *TmTak1* | 29.59 |
| LL | *TmTak1* | 26.12 | A1 | *TmTak1* | 28.85 |
| LL | *TmL27a* | 19.23 | A1 | *TmL27a* | 22.69 |
| LL | *TmL27a* | 19.11 | A1 | *TmL27a* | 22.12 |
| PP | *TmTak1* | 26.23 | A2 | *TmTak1* | 24.07 |
| PP | *TmTak1* | 25.93 | A2 | *TmTak1* | 23.9 |
| PP | *TmL27a* | 17.77 | A2 | *TmL27a* | 16.98 |
| PP | *TmL27a* | 17.69 | A2 | *TmL27a* | 16.93 |
| P1 | *TmTak1* | 24.14 | A3 | *TmTak1* | 25.9 |
| P1 | *TmTak1* | 24.31 | A3 | *TmTak1* | 25.66 |
| P1 | *TmL27a* | 17.49 | A3 | *TmL27a* | 17.51 |
| P1 | *TmL27a* | 17.7 | A3 | *TmL27a* | 17.37 |
| P2 | *TmTak1* | 24.24 | A4 | *TmTak1* | 25.94 |
| P2 | *TmTak1* | 24.23 | A4 | *TmTak1* | 25.75 |
| P2 | *TmL27a* | 17.35 | A4 | *TmL27a* | 17.33 |
| P2 | *TmL27a* | 17.35 | A4 | *TmL27a* | 18.15 |
| P3 | *TmTak1* | 28.83 | A5 | *TmTak1* | 24.66 |
| P3 | *TmTak1* | 27.42 | A5 | *TmTak1* | 23.79 |
| P3 | *TmL27a* | 20.91 | A5 | *TmL27a* | 17.19 |
| P3 | *TmL27a* | 20.65 | A5 | *TmL27a* | 17.18 |
| P4 | *TmTak1* | 23.8 |  |  |  |
| P4 | *TmTak1* | 23.72 |  |  |  |
| P4 | *TmL27a* | 16.89 |  |  |  |
| P4 | *TmL27a* | 17.25 |  |  |  |
| P5 | *TmTak1* | 26.77 |  |  |  |
| P5 | *TmTak1* | 26.92 |  |  |  |
| P5 | *TmL27a* | 19.03 |  |  |  |
| P5 | *TmL27a* | 19.26 |  |  |  |

**Supplementary Table3. Raw data of qPCR in tissues.**

Cq data of *TmTak1* and *TmL27a* in *T. molitor* at Fat bodies (FB), gut (GT), hemocytes (HC), integument (INT), and Malpighian tubules (MT) of late instar larvae and adults, in addition to ovaries (OV) and testes (TT) of adults

| Sample name | Gene name | Cq (∆R) | Sample name | Gene name | Cq (∆R) |
| --- | --- | --- | --- | --- | --- |
| Larva FB | *TmTak1* | 27.35 | Adult FB | *TmTak1* | 23.52 |
| Larva FB | *TmTak1* | 27.42 | Adult FB | *TmTak1* | 23.31 |
| Larva FB | *TmL27a* | 18.87 | Adult FB | *TmL27a* | 18.23 |
| Larva FB | *TmL27a* | 18.83 | Adult FB | *TmL27a* | 18.13 |
| Larva GT | *TmTak1* | 23.98 | Adult GT | *TmTak1* | 25.78 |
| Larva GT | *TmTak1* | 24.11 | Adult GT | *TmTak1* | 25.85 |
| Larva GT | *TmL27a* | 19.86 | Adult GT | *TmL27a* | 17.75 |
| Larva GT | *TmL27a* | 19.83 | Adult GT | *TmL27a* | 17.35 |
| Larva HL | *TmTak1* | 32.46 | Adult HL | *TmTak1* | 31.68 |
| Larva HL | *TmTak1* | 33.4 | Adult HL | *TmTak1* | 31.94 |
| Larva HL | *TmL27a* | 26.99 | Adult HL | *TmL27a* | 24.79 |
| Larva HL | *TmL27a* | 27.56 | Adult HL | *TmL27a* | 24.68 |
| Larva INT | *TmTak1* | 27.6 | Adult INT | *TmTak1* | 28.15 |
| Larva INT | *TmTak1* | 27.64 | Adult INT | *TmTak1* | 28.61 |
| Larva INT | *TmL27a* | 18.5 | Adult INT | *TmL27a* | 19.7 |
| Larva INT | *TmL27a* | 18.52 | Adult INT | *TmL27a* | 19.92 |
| Larva MT | *TmTak1* | 25.74 | Adult MT | *TmTak1* | 26.17 |
| Larva MT | *TmTak1* | 25.81 | Adult MT | *TmTak1* | 25.97 |
| Larva MT | *TmL27a* | 17.8 | Adult MT | *TmL27a* | 17.71 |
| Larva MT | *TmL27a* | 17.85 | Adult MT | *TmL27a* | 17.8 |
|  |  |  | Adult OV | *TmTak1* | 28.29 |
|  |  |  | Adult OV | *TmTak1* | 28.5 |
|  |  |  | Adult OV | *TmL27a* | 20.15 |
|  |  |  | Adult OV | *TmL27a* | 19.95 |
|  |  |  | Adult TT | *TmTak1* | 27.05 |
|  |  |  | Adult TT | *TmTak1* | 26.82 |
|  |  |  | Adult TT | *TmL27a* | 18.97 |
|  |  |  | Adult TT | *TmL27a* | 18.46 |

**Supplementary Table4. Raw data of qPCR in microbial challenge.**

Cq data of *TmTak1* and *TmL27a* in *T. molitor* at whole body(WB)

| Sample name | Gene name | Cq (∆R) | Sample name | Gene name | Cq (∆R) |
| --- | --- | --- | --- | --- | --- |
| PBS 3h WB | *TmTak1* | 24.32 | *S. aureus* 3h WB | *TmTak1* | 23.31 |
| PBS 3h WB | *TmTak1* | 24.38 | *S. aureus* 3h WB | *TmTak1* | 23.21 |
| PBS 3h WB | *TmL27a* | 16.7 | *S. aureus* 3h WB | *TmL27a* | 16.07 |
| PBS 3h WB | *TmL27a* | 16.53 | *S. aureus* 3h WB | *TmL27a* | 16.09 |
| PBS 6h WB | *TmTak1* | 22.56 | *S. aureus* 6h WB | *TmTak1* | 24.99 |
| PBS 6h WB | *TmTak1* | 22.4 | *S. aureus* 6h WB | *TmTak1* | 24.72 |
| PBS 6h WB | *TmL27a* | 15.57 | *S. aureus* 6h WB | *TmL27a* | 17.77 |
| PBS 6h WB | *TmL27a* | 15.37 | *S. aureus* 6h WB | *TmL27a* | 17.77 |
| PBS 9h WB | *TmTak1* | 22.54 | *S. aureus* 9h WB | *TmTak1* | 24.16 |
| PBS 9h WB | *TmTak1* | 22.4 | *S. aureus* 9h WB | *TmTak1* | 24.42 |
| PBS 9h WB | *TmL27a* | 15.58 | *S. aureus* 9h WB | *TmL27a* | 16.25 |
| PBS 9h WB | *TmL27a* | 15.5 | *S. aureus* 9h WB | *TmL27a* | 16.79 |
| PBS 12h WB | *TmTak1* | 23.17 | *S. aureus* 12h WB | *TmTak1* | 24.5 |
| PBS 12h WB | *TmTak1* | 23.45 | *S. aureus* 12h WB | *TmTak1* | 25.08 |
| PBS 12h WB | *TmL27a* | 16.02 | *S. aureus* 12h WB | *TmL27a* | 17.94 |
| PBS 12h WB | *TmL27a* | 15.93 | *S. aureus* 12h WB | *TmL27a* | 17.99 |
| PBS 24h WB | *TmTak1* | 22.27 | *S. aureus* 24h WB | *TmTak1* | 24.04 |
| PBS 24h WB | *TmTak1* | 22.14 | *S. aureus* 24h WB | *TmTak1* | 24.29 |
| PBS 24h WB | *TmL27a* | 15.11 | *S. aureus* 24h WB | *TmL27a* | 17.05 |
| PBS 24h WB | *TmL27a* | 15.21 | *S. aureus* 24h WB | *TmL27a* | 17.05 |
| *E. coli* 3h WB | *TmTak1* | 22.75 | *C. albicans* 3h WB | *TmTak1* | 24.03 |
| *E. coli* 3h WB | *TmTak1* | 22.95 | *C. albicans* 3h WB | *TmTak1* | 24.07 |
| *E. coli* 3h WB | *TmL27a* | 16.1 | *C. albicans* 3h WB | *TmL27a* | 17.08 |
| *E. coli* 3h WB | *TmL27a* | 16.11 | *C. albicans* 3h WB | *TmL27a* | 17.18 |
| *E. coli* 6h WB | *TmTak1* | 23.32 | *C. albicans* 6h WB | *TmTak1* | 23.91 |
| *E. coli* 6h WB | *TmTak1* | 23.25 | *C. albicans* 6h WB | *TmTak1* | 23.93 |
| *E. coli* 6h WB | *TmL27a* | 16.81 | *C. albicans* 6h WB | *TmL27a* | 16.81 |
| *E. coli* 6h WB | *TmL27a* | 16.85 | *C. albicans* 6h WB | *TmL27a* | 17.1 |
| *E. coli* 9h WB | *TmTak1* | 23.06 | *C. albicans* 9h WB | *TmTak1* | 22.95 |
| *E. coli* 9h WB | *TmTak1* | 22.97 | *C. albicans* 9h WB | *TmTak1* | 22.86 |
| *E. coli* 9h WB | *TmL27a* | 16.86 | *C. albicans* 9h WB | *TmL27a* | 15.9 |
| *E. coli* 9h WB | *TmL27a* | 16.88 | *C. albicans* 9h WB | *TmL27a* | 15.59 |
| *E. coli* 12h WB | *TmTak1* | 25.04 | *C. albicans* 12h WB | *TmTak1* | 23.65 |
| *E. coli* 12h WB | *TmTak1* | 24.64 | *C. albicans* 12h WB | *TmTak1* | 23.88 |
| *E. coli* 12h WB | *TmL27a* | 17.9 | *C. albicans* 12h WB | *TmL27a* | 15.62 |
| *E. coli* 12h WB | *TmL27a* | 17.79 | *C. albicans* 12h WB | *TmL27a* | 15.9 |
| *E. coli* 24h WB | *TmTak1* | 23.88 | *C. albicans* 24h WB | *TmTak1* | 22.35 |
| *E. coli* 24h WB | *TmTak1* | 23.85 | *C. albicans* 24h WB | *TmTak1* | 22.29 |
| *E. coli* 24h WB | *TmL27a* | 16.08 | *C. albicans* 24h WB | *TmL27a* | 15.16 |
| *E. coli* 24h WB | *TmL27a* | 16.19 | *C. albicans* 24h WB | *TmL27a* | 15.19 |

**Supplementary Table5. Raw data of qPCR in microbial challenge.**

Cq data of *TmTak1* and *TmL27a* in *T. molitor* at Gut(GT).

| Sample name | Gene name | Cq (∆R) | Sample name | Gene name | Cq (∆R) |
| --- | --- | --- | --- | --- | --- |
| PBS 3h GT | *TmTak1* | 24.58 | *S. aureus* 3h GT | *TmTak1* | 24.74 |
| PBS 3h GT | *TmTak1* | 24.68 | *S. aureus* 3h GT | *TmTak1* | 25.65 |
| PBS 3h GT | *TmL27a* | 18.26 | *S. aureus* 3h GT | *TmL27a* | 18.22 |
| PBS 3h GT | *TmL27a* | 18.27 | *S. aureus* 3h GT | *TmL27a* | 18.39 |
| PBS 6h GT | *TmTak1* | 25.48 | *S. aureus* 6h GT | *TmTak1* | 24.36 |
| PBS 6h GT | *TmTak1* | 25.36 | *S. aureus* 6h GT | *TmTak1* | 24.19 |
| PBS 6h GT | *TmL27a* | 18.47 | *S. aureus* 6h GT | *TmL27a* | 17.5 |
| PBS 6h GT | *TmL27a* | 18.48 | *S. aureus* 6h GT | *TmL27a* | 17.64 |
| PBS 9h GT | *TmTak1* | 22.75 | *S. aureus* 9h GT | *TmTak1* | 23.59 |
| PBS 9h GT | *TmTak1* | 22.95 | *S. aureus* 9h GT | *TmTak1* | 23.94 |
| PBS 9h GT | *TmL27a* | 18.04 | *S. aureus* 9h GT | *TmL27a* | 17.06 |
| PBS 9h GT | *TmL27a* | 18.05 | *S. aureus* 9h GT | *TmL27a* | 16.86 |
| PBS 12h GT | *TmTak1* | 22.91 | *S. aureus* 12h GT | *TmTak1* | 25.18 |
| PBS 12h GT | *TmTak1* | 22.28 | *S. aureus* 12h GT | *TmTak1* | 25.72 |
| PBS 12h GT | *TmL27a* | 19.3 | *S. aureus* 12h GT | *TmL27a* | 17.28 |
| PBS 12h GT | *TmL27a* | 19.11 | *S. aureus* 12h GT | *TmL27a* | 16.73 |
| PBS 24h GT | *TmTak1* | 21.82 | *S. aureus* 24h GT | *TmTak1* | 24.46 |
| PBS 24h GT | *TmTak1* | 21.43 | *S. aureus* 24h GT | *TmTak1* | 24.96 |
| PBS 24h GT | *TmL27a* | 18.31 | *S. aureus* 24h GT | *TmL27a* | 17.21 |
| PBS 24h GT | *TmL27a* | 18.27 | *S. aureus* 24h GT | *TmL27a* | 17.28 |
| *E. coli* 3h GT | *TmTak1* | 24.72 | *C. albicans* 3h GT | *TmTak1* | 23.47 |
| *E. coli* 3h GT | *TmTak1* | 24.92 | *C. albicans* 3h GT | *TmTak1* | 23.48 |
| *E. coli* 3h GT | *TmL27a* | 18.07 | *C. albicans* 3h GT | *TmL27a* | 16.52 |
| *E. coli* 3h GT | *TmL27a* | 17.97 | *C. albicans* 3h GT | *TmL27a* | 16.71 |
| *E. coli* 6h GT | *TmTak1* | 25.32 | *C. albicans* 6h GT | *TmTak1* | 24.34 |
| *E. coli* 6h GT | *TmTak1* | 25.45 | *C. albicans* 6h GT | *TmTak1* | 24.48 |
| *E. coli* 6h GT | *TmL27a* | 18.22 | *C. albicans* 6h GT | *TmL27a* | 17.77 |
| *E. coli* 6h GT | *TmL27a* | 18.22 | *C. albicans* 6h GT | *TmL27a* | 18.05 |
| *E. coli* 9h GT | *TmTak1* | 24.35 | *C. albicans* 9h GT | *TmTak1* | 25.47 |
| *E. coli* 9h GT | *TmTak1* | 24.54 | *C. albicans* 9h GT | *TmTak1* | 24.95 |
| *E. coli* 9h GT | *TmL27a* | 18.05 | *C. albicans* 9h GT | *TmL27a* | 18.36 |
| *E. coli* 9h GT | *TmL27a* | 18.06 | *C. albicans* 9h GT | *TmL27a* | 18.26 |
| *E. coli* 12h GT | *TmTak1* | 25.73 | *C. albicans* 12h GT | *TmTak1* | 23.23 |
| *E. coli* 12h GT | *TmTak1* | 25.19 | *C. albicans* 12h GT | *TmTak1* | 23.07 |
| *E. coli* 12h GT | *TmL27a* | 18.35 | *C. albicans* 12h GT | *TmL27a* | 18.49 |
| *E. coli* 12h GT | *TmL27a* | 18.41 | *C. albicans* 12h GT | *TmL27a* | 18.78 |
| *E. coli* 24h GT | *TmTak1* | 23.98 | *C. albicans* 24h GT | *TmTak1* | 24.09 |
| *E. coli* 24h GT | *TmTak1* | 23.92 | *C. albicans* 24h GT | *TmTak1* | 24.18 |
| *E. coli* 24h GT | *TmL27a* | 17.13 | *C. albicans* 24h GT | *TmL27a* | 18.58 |
| *E. coli* 24h GT | *TmL27a* | 17.32 | *C. albicans* 24h GT | *TmL27a* | 19.65 |

**Supplementary Table6. Raw data of qPCR in microbial challenge.**

Cq data of *TmTak1* and *TmL27a* in *T. molitor* at Fat bodies(FB).

| Sample name | Gene name | Cq (∆R) | Sample name | Gene name | Cq (∆R) |
| --- | --- | --- | --- | --- | --- |
| PBS 3h FB | *TmTak1* | 26.26 | *S. aureus* 3h FB | *TmTak1* | 25.72 |
| PBS 3h FB | *TmTak1* | 26.19 | *S. aureus* 3h FB | *TmTak1* | 26.71 |
| PBS 3h FB | *TmL27a* | 19.51 | *S. aureus* 3h FB | *TmL27a* | 18.57 |
| PBS 3h FB | *TmL27a* | 19.41 | *S. aureus* 3h FB | *TmL27a* | 18.7 |
| PBS 6h FB | *TmTak1* | 26.82 | *S. aureus* 6h FB | *TmTak1* | 26.95 |
| PBS 6h FB | *TmTak1* | 26.71 | *S. aureus* 6h FB | *TmTak1* | 26.57 |
| PBS 6h FB | *TmL27a* | 19.59 | *S. aureus* 6h FB | *TmL27a* | 19.65 |
| PBS 6h FB | *TmL27a* | 19.35 | *S. aureus* 6h FB | *TmL27a* | 19.75 |
| PBS 9h FB | *TmTak1* | 26.81 | *S. aureus* 9h FB | *TmTak1* | 26.71 |
| PBS 9h FB | *TmTak1* | 26.95 | *S. aureus* 9h FB | *TmTak1* | 27.39 |
| PBS 9h FB | *TmL27a* | 19.65 | *S. aureus* 9h FB | *TmL27a* | 19.48 |
| PBS 9h FB | *TmL27a* | 19.42 | *S. aureus* 9h FB | *TmL27a* | 19.47 |
| PBS 12h FB | *TmTak1* | 25.59 | *S. aureus* 12h FB | *TmTak1* | 25.52 |
| PBS 12h FB | *TmTak1* | 25.66 | *S. aureus* 12h FB | *TmTak1* | 25.59 |
| PBS 12h FB | *TmL27a* | 18.75 | *S. aureus* 12h FB | *TmL27a* | 18.35 |
| PBS 12h FB | *TmL27a* | 18.91 | *S. aureus* 12h FB | *TmL27a* | 18.54 |
| PBS 24h FB | *TmTak1* | 25.59 | *S. aureus* 24h FB | *TmTak1* | 26.1 |
| PBS 24h FB | *TmTak1* | 25.54 | *S. aureus* 24h FB | *TmTak1* | 27.45 |
| PBS 24h FB | *TmL27a* | 19.02 | *S. aureus* 24h FB | *TmL27a* | 18.44 |
| PBS 24h FB | *TmL27a* | 18.48 | *S. aureus* 24h FB | *TmL27a* | 19.27 |
| *E. coli* 3h FB | *TmTak1* | 27.7 | *C. albicans* 3h FB | *TmTak1* | 26.4 |
| *E. coli* 3h FB | *TmTak1* | 27.84 | *C. albicans* 3h FB | *TmTak1* | 26.24 |
| *E. coli* 3h FB | *TmL27a* | 20.61 | *C. albicans* 3h FB | *TmL27a* | 18.57 |
| *E. coli* 3h FB | *TmL27a* | 20.44 | *C. albicans* 3h FB | *TmL27a* | 18.94 |
| *E. coli* 6h FB | *TmTak1* | 27.03 | *C. albicans* 6h FB | *TmTak1* | 26.22 |
| *E. coli* 6h FB | *TmTak1* | 27.2 | *C. albicans* 6h FB | *TmTak1* | 26.46 |
| *E. coli* 6h FB | *TmL27a* | 19.81 | *C. albicans* 6h FB | *TmL27a* | 19.72 |
| *E. coli* 6h FB | *TmL27a* | 20.06 | *C. albicans* 6h FB | *TmL27a* | 19.87 |
| *E. coli* 9h FB | *TmTak1* | 27.76 | *C. albicans* 9h FB | *TmTak1* | 28.67 |
| *E. coli* 9h FB | *TmTak1* | 27.42 | *C. albicans* 9h FB | *TmTak1* | 28.44 |
| *E. coli* 9h FB | *TmL27a* | 20.11 | *C. albicans* 9h FB | *TmL27a* | 22.19 |
| *E. coli* 9h FB | *TmL27a* | 20.24 | *C. albicans* 9h FB | *TmL27a* | 21.87 |
| *E. coli* 12h FB | *TmTak1* | 27.1 | *C. albicans* 12h FB | *TmTak1* | 25.95 |
| *E. coli* 12h FB | *TmTak1* | 27.17 | *C. albicans* 12h FB | *TmTak1* | 26.24 |
| *E. coli* 12h FB | *TmL27a* | 20.25 | *C. albicans* 12h FB | *TmL27a* | 18.87 |
| *E. coli* 12h FB | *TmL27a* | 20.34 | *C. albicans* 12h FB | *TmL27a* | 19.03 |
| *E. coli* 24h FB | *TmTak1* | 26.8 | *C. albicans* 24h FB | *TmTak1* | 27.17 |
| *E. coli* 24h FB | *TmTak1* | 26.93 | *C. albicans* 24h FB | *TmTak1* | 26.51 |
| *E. coli* 24h FB | *TmL27a* | 19.28 | *C. albicans* 24h FB | *TmL27a* | 19.06 |
| *E. coli* 24h FB | *TmL27a* | 19.34 | *C. albicans* 24h FB | *TmL27a* | 18.78 |

**Supplementary Table7. Raw data of qPCR in microbial challenge.**

Cq data of *TmTak1* and *TmL27a* in *T. molitor* at Hemocytes(HC).

| Sample name | Gene name | Cq (∆R) | Sample name | Gene name | Cq (∆R) |
| --- | --- | --- | --- | --- | --- |
| PBS 3h HC | *TmTak1* | 28.69 | *S. aureus* 3h HC | *TmTak1* | 32.43 |
| PBS 3h HC | *TmTak1* | 28.9 | *S. aureus* 3h HC | *TmTak1* | 32.85 |
| PBS 3h HC | *TmL27a* | 27.43 | *S. aureus* 3h HC | *TmL27a* | 27.43 |
| PBS 3h HC | *TmL27a* | 27.77 | *S. aureus* 3h HC | *TmL27a* | 27.6 |
| PBS 6h HC | *TmTak1* | 31.91 | *S. aureus* 6h HC | *TmTak1* | 32.46 |
| PBS 6h HC | *TmTak1* | 30.55 | *S. aureus* 6h HC | *TmTak1* | 35.48 |
| PBS 6h HC | *TmL27a* | 27.41 | *S. aureus* 6h HC | *TmL27a* | 28.03 |
| PBS 6h HC | *TmL27a* | 27.24 | *S. aureus* 6h HC | *TmL27a* | 28.63 |
| PBS 9h HC | *TmTak1* | 30.07 | *S. aureus* 9h HC | *TmTak1* | 29.79 |
| PBS 9h HC | *TmTak1* | 31.33 | *S. aureus* 9h HC | *TmTak1* | 29.59 |
| PBS 9h HC | *TmL27a* | 27.15 | *S. aureus* 9h HC | *TmL27a* | 27.07 |
| PBS 9h HC | *TmL27a* | 27.1 | *S. aureus* 9h HC | *TmL27a* | 26.96 |
| PBS 12h HC | *TmTak1* | 31 | *S. aureus* 12h HC | *TmTak1* | 31.92 |
| PBS 12h HC | *TmTak1* | 31.89 | *S. aureus* 12h HC | *TmTak1* | 30.1 |
| PBS 12h HC | *TmL27a* | 27.05 | *S. aureus* 12h HC | *TmL27a* | 28.89 |
| PBS 12h HC | *TmL27a* | 26.92 | *S. aureus* 12h HC | *TmL27a* | 28.54 |
| PBS 24h HC | *TmTak1* | 31.83 | *S. aureus* 24h HC | *TmTak1* | 31.14 |
| PBS 24h HC | *TmTak1* | 30.9 | *S. aureus* 24h HC | *TmTak1* | 30.62 |
| PBS 24h HC | *TmL27a* | 27.62 | *S. aureus* 24h HC | *TmL27a* | 25.9 |
| PBS 24h HC | *TmL27a* | 27.63 | *S. aureus* 24h HC | *TmL27a* | 25.81 |
| *E. coli* 3h HC | *TmTak1* | 28.76 | *C. albicans* 3h HC | *TmTak1* | 28.77 |
| *E. coli* 3h HC | *TmTak1* | 29.17 | *C. albicans* 3h HC | *TmTak1* | 28.31 |
| *E. coli* 3h HC | *TmL27a* | 24.81 | *C. albicans* 3h HC | *TmL27a* | 26.59 |
| *E. coli* 3h HC | *TmL27a* | 24.53 | *C. albicans* 3h HC | *TmL27a* | 26.54 |
| *E. coli* 6h HC | *TmTak1* | 32.23 | *C. albicans* 6h HC | *TmTak1* | 33.71 |
| *E. coli* 6h HC | *TmTak1* | 33.79 | *C. albicans* 6h HC | *TmTak1* | 32.41 |
| *E. coli* 6h HC | *TmL27a* | 27.62 | *C. albicans* 6h HC | *TmL27a* | 29.34 |
| *E. coli* 6h HC | *TmL27a* | 27.66 | *C. albicans* 6h HC | *TmL27a* | 28.87 |
| *E. coli* 9h HC | *TmTak1* | 26.51 | *C. albicans* 9h HC | *TmTak1* | 31.65 |
| *E. coli* 9h HC | *TmTak1* | 26.02 | *C. albicans* 9h HC | *TmTak1* | 32.09 |
| *E. coli* 9h HC | *TmL27a* | 20.04 | *C. albicans* 9h HC | *TmL27a* | 27.66 |
| *E. coli* 9h HC | *TmL27a* | 19.94 | *C. albicans* 9h HC | *TmL27a* | 27.77 |
| *E. coli* 12h HC | *TmTak1* | 27.67 | *C. albicans* 12h HC | *TmTak1* | 32.9 |
| *E. coli* 12h HC | *TmTak1* | 27.19 | *C. albicans* 12h HC | *TmTak1* | 32.9 |
| *E. coli* 12h HC | *TmL27a* | 20.27 | *C. albicans* 12h HC | *TmL27a* | 27.42 |
| *E. coli* 12h HC | *TmL27a* | 20.4 | *C. albicans* 12h HC | *TmL27a* | 27.71 |
| *E. coli* 24h HC | *TmTak1* | 24.56 | *C. albicans* 24h HC | *TmTak1* | 29.76 |
| *E. coli* 24h HC | *TmTak1* | 24.68 | *C. albicans* 24h HC | *TmTak1* | 29.34 |
| *E. coli* 24h HC | *TmL27a* | 17.87 | *C. albicans* 24h HC | *TmL27a* | 28.69 |
| *E. coli* 24h HC | *TmL27a* | 17.91 | *C. albicans* 24h HC | *TmL27a* | 28.3 |

**Supplementary Table8. Raw data of qPCR in microbial challenge.**

Cq data of *TmTak1* and *TmL27a* in *T. molitor* at Malpighian tubules.

| Sample name | Gene name | Cq (∆R) | Sample name | Gene name | Cq (∆R) |
| --- | --- | --- | --- | --- | --- |
| PBS 3h MT | *TmTak1* | 24.65 | *S. aureus* 3h MT | *TmTak1* | 23.89 |
| PBS 3h MT | *TmTak1* | 24.5 | *S. aureus* 3h MT | *TmTak1* | 22.78 |
| PBS 3h MT | *TmL27a* | 18.41 | *S. aureus* 3h MT | *TmL27a* | 19.91 |
| PBS 3h MT | *TmL27a* | 18.15 | *S. aureus* 3h MT | *TmL27a* | 19.57 |
| PBS 6h MT | *TmTak1* | 24.87 | *S. aureus* 6h MT | *TmTak1* | 23.5 |
| PBS 6h MT | *TmTak1* | 25.04 | *S. aureus* 6h MT | *TmTak1* | 23.21 |
| PBS 6h MT | *TmL27a* | 18.13 | *S. aureus* 6h MT | *TmL27a* | 18.43 |
| PBS 6h MT | *TmL27a* | 18.25 | *S. aureus* 6h MT | *TmL27a* | 18.53 |
| PBS 9h MT | *TmTak1* | 25.31 | *S. aureus* 9h MT | *TmTak1* | 23.5 |
| PBS 9h MT | *TmTak1* | 25.64 | *S. aureus* 9h MT | *TmTak1* | 23.21 |
| PBS 9h MT | *TmL27a* | 18.63 | *S. aureus* 9h MT | *TmL27a* | 18.43 |
| PBS 9h MT | *TmL27a* | 18.55 | *S. aureus* 9h MT | *TmL27a* | 18.53 |
| PBS 12h MT | *TmTak1* | 23.13 | *S. aureus* 12h MT | *TmTak1* | 23.89 |
| PBS 12h MT | *TmTak1* | 23.73 | *S. aureus* 12h MT | *TmTak1* | 23.98 |
| PBS 12h MT | *TmL27a* | 18.27 | *S. aureus* 12h MT | *TmL27a* | 18.16 |
| PBS 12h MT | *TmL27a* | 18.05 | *S. aureus* 12h MT | *TmL27a* | 18.38 |
| PBS 24h MT | *TmTak1* | 23.04 | *S. aureus* 24h MT | *TmTak1* | 23.07 |
| PBS 24h MT | *TmTak1* | 23.26 | *S. aureus* 24h MT | *TmTak1* | 23.36 |
| PBS 24h MT | *TmL27a* | 19.3 | *S. aureus* 24h MT | *TmL27a* | 18.35 |
| PBS 24h MT | *TmL27a* | 19.22 | *S. aureus* 24h MT | *TmL27a* | 18.43 |
| *E. coli* 3h MT | *TmTak1* | 27.06 | *C. albicans* 3h MT | *TmTak1* | 26.6 |
| *E. coli* 3h MT | *TmTak1* | 27.06 | *C. albicans* 3h MT | *TmTak1* | 26.08 |
| *E. coli* 3h MT | *TmL27a* | 20.43 | *C. albicans* 3h MT | *TmL27a* | 22.69 |
| *E. coli* 3h MT | *TmL27a* | 20.34 | *C. albicans* 3h MT | *TmL27a* | 22.85 |
| *E. coli* 6h MT | *TmTak1* | 25.84 | *C. albicans* 6h MT | *TmTak1* | 23.61 |
| *E. coli* 6h MT | *TmTak1* | 24.85 | *C. albicans* 6h MT | *TmTak1* | 23.78 |
| *E. coli* 6h MT | *TmL27a* | 19.34 | *C. albicans* 6h MT | *TmL27a* | 21.24 |
| *E. coli* 6h MT | *TmL27a* | 19.3 | *C. albicans* 6h MT | *TmL27a* | 21.39 |
| *E. coli* 9h MT | *TmTak1* | 26.54 | *C. albicans* 9h MT | *TmTak1* | 24.42 |
| *E. coli* 9h MT | *TmTak1* | 27.46 | *C. albicans* 9h MT | *TmTak1* | 25.48 |
| *E. coli* 9h MT | *TmL27a* | 20.44 | *C. albicans* 9h MT | *TmL27a* | 21.59 |
| *E. coli* 9h MT | *TmL27a* | 20.41 | *C. albicans* 9h MT | *TmL27a* | 21.66 |
| *E. coli* 12h MT | *TmTak1* | 26.49 | *C. albicans* 12h MT | *TmTak1* | 23.62 |
| *E. coli* 12h MT | *TmTak1* | 26.35 | *C. albicans* 12h MT | *TmTak1* | 23.63 |
| *E. coli* 12h MT | *TmL27a* | 20.29 | *C. albicans* 12h MT | *TmL27a* | 22.31 |
| *E. coli* 12h MT | *TmL27a* | 20.18 | *C. albicans* 12h MT | *TmL27a* | 22.53 |
| *E. coli* 24h MT | *TmTak1* | 26.82 | *C. albicans* 24h MT | *TmTak1* | 23.48 |
| *E. coli* 24h MT | *TmTak1* | 25.69 | *C. albicans* 24h MT | *TmTak1* | 24.44 |
| *E. coli* 24h MT | *TmL27a* | 21.36 | *C. albicans* 24h MT | *TmL27a* | 20.61 |
| *E. coli* 24h MT | *TmL27a* | 21.33 | *C. albicans* 24h MT | *TmL27a* | 20.91 |

**Supplementary Table9. Raw data of qPCR in microbial challenge.**

Cq data of *TmTak1* and *TmL27a* in *T. molitor* at integument(IT).

| Sample name | Gene name | Cq (∆R) | Sample name | Gene name | Cq (∆R) |
| --- | --- | --- | --- | --- | --- |
| PBS 3h IT | *TmTak1* | 25 | *S. aureus* 3h IT | *TmTak1* | 24.64 |
| PBS 3h IT | *TmTak1* | 24.7 | *S. aureus* 3h IT | *TmTak1* | 24.46 |
| PBS 3h IT | *TmL27a* | 17.3 | *S. aureus* 3h IT | *TmL27a* | 17.16 |
| PBS 3h IT | *TmL27a* | 17.07 | *S. aureus* 3h IT | *TmL27a* | 17.16 |
| PBS 6h IT | *TmTak1* | 24.29 | *S. aureus* 6h IT | *TmTak1* | 25.5 |
| PBS 6h IT | *TmTak1* | 23.96 | *S. aureus* 6h IT | *TmTak1* | 25.69 |
| PBS 6h IT | *TmL27a* | 16.8 | *S. aureus* 6h IT | *TmL27a* | 17.5 |
| PBS 6h IT | *TmL27a* | 17.04 | *S. aureus* 6h IT | *TmL27a* | 17.57 |
| PBS 9h IT | *TmTak1* | 23.88 | *S. aureus* 9h IT | *TmTak1* | 24.17 |
| PBS 9h IT | *TmTak1* | 23.75 | *S. aureus* 9h IT | *TmTak1* | 24.31 |
| PBS 9h IT | *TmL27a* | 16.57 | *S. aureus* 9h IT | *TmL27a* | 16.74 |
| PBS 9h IT | *TmL27a* | 16.94 | *S. aureus* 9h IT | *TmL27a* | 16.96 |
| PBS 12h IT | *TmTak1* | 24.21 | *S. aureus* 12h IT | *TmTak1* | 24.68 |
| PBS 12h IT | *TmTak1* | 23.92 | *S. aureus* 12h IT | *TmTak1* | 24.77 |
| PBS 12h IT | *TmL27a* | 16.63 | *S. aureus* 12h IT | *TmL27a* | 17.35 |
| PBS 12h IT | *TmL27a* | 16.34 | *S. aureus* 12h IT | *TmL27a* | 17.38 |
| PBS 24h IT | *TmTak1* | 23.71 | *S. aureus* 24h IT | *TmTak1* | 25.96 |
| PBS 24h IT | *TmTak1* | 23.55 | *S. aureus* 24h IT | *TmTak1* | 26.54 |
| PBS 24h IT | *TmL27a* | 16.21 | *S. aureus* 24h IT | *TmL27a* | 18.78 |
| PBS 24h IT | *TmL27a* | 16.28 | *S. aureus* 24h IT | *TmL27a* | 19 |
| *E. coli* 3h IT | *TmTak1* | 25.5 | *C. albicans* 3h IT | *TmTak1* | 26.76 |
| *E. coli* 3h IT | *TmTak1* | 25.57 | *C. albicans* 3h IT | *TmTak1* | 26.95 |
| *E. coli* 3h IT | *TmL27a* | 17.84 | *C. albicans* 3h IT | *TmL27a* | 19.41 |
| *E. coli* 3h IT | *TmL27a* | 17.87 | *C. albicans* 3h IT | *TmL27a* | 19.34 |
| *E. coli* 6h IT | *TmTak1* | 24.88 | *C. albicans* 6h IT | *TmTak1* | 25.16 |
| *E. coli* 6h IT | *TmTak1* | 25.19 | *C. albicans* 6h IT | *TmTak1* | 25.34 |
| *E. coli* 6h IT | *TmL27a* | 18.07 | *C. albicans* 6h IT | *TmL27a* | 17.34 |
| *E. coli* 6h IT | *TmL27a* | 18.11 | *C. albicans* 6h IT | *TmL27a* | 18.3 |
| *E. coli* 9h IT | *TmTak1* | 25.94 | *C. albicans* 9h IT | *TmTak1* | 23.48 |
| *E. coli* 9h IT | *TmTak1* | 26.33 | *C. albicans* 9h IT | *TmTak1* | 22.68 |
| *E. coli* 9h IT | *TmL27a* | 18.22 | *C. albicans* 9h IT | *TmL27a* | 18.53 |
| *E. coli* 9h IT | *TmL27a* | 18.53 | *C. albicans* 9h IT | *TmL27a* | 18.33 |
| *E. coli* 12h IT | *TmTak1* | 26.24 | *C. albicans* 12h IT | *TmTak1* | 23.57 |
| *E. coli* 12h IT | *TmTak1* | 26.3 | *C. albicans* 12h IT | *TmTak1* | 24.08 |
| *E. coli* 12h IT | *TmL27a* | 18.49 | *C. albicans* 12h IT | *TmL27a* | 17.34 |
| *E. coli* 12h IT | *TmL27a* | 18.38 | *C. albicans* 12h IT | *TmL27a* | 17.24 |
| *E. coli* 24h IT | *TmTak1* | 25.12 | *C. albicans* 24h IT | *TmTak1* | 24.69 |
| *E. coli* 24h IT | *TmTak1* | 25.43 | *C. albicans* 24h IT | *TmTak1* | 25.02 |
| *E. coli* 24h IT | *TmL27a* | 17.74 | *C. albicans* 24h IT | *TmL27a* | 17.21 |
| *E. coli* 24h IT | *TmL27a* | 18.07 | *C. albicans* 24h IT | *TmL27a* | 17.3 |

**Supplementary Table10. Raw data of qPCR in AMP expression patterns.**

Cq data of *TmTak1* and *TmL27a* in *T. molitor* at Gut(GT).

| Sample name | Gene name | Cq (∆R) | Sample name | Gene name | Cq (∆R) |
| --- | --- | --- | --- | --- | --- |
| EGFP PBS | *TmTene1* | 23.43 | EGFP PBS | *TmTene2* | 30.36 |
| EGFP PBS | *TmTene1* | 23.25 | EGFP PBS | *TmTene2* | 29.81 |
| EGFP PBS | *TmL27a* | 19.33 | EGFP PBS | *TmL27a* | 19.33 |
| EGFP PBS | *TmL27a* | 19.2 | EGFP PBS | *TmL27a* | 19.2 |
| TAK1 PBS | *TmTene1* | 21.8 | TAK1 PBS | *TmTene2* | 26.17 |
| TAK1 PBS | *TmTene1* | 21.14 | TAK1 PBS | *TmTene2* | 25.77 |
| TAK1 PBS | *TmL27a* | 18.55 | TAK1 PBS | *TmL27a* | 18.55 |
| TAK1 PBS | *TmL27a* | 18.34 | TAK1 PBS | *TmL27a* | 18.34 |
| EGFP *E. coli* | *TmTene1* | 15.29 | EGFP *E. coli* | *TmTene2* | 19.91 |
| EGFP *E. coli* | *TmTene1* | 15.44 | EGFP *E. coli* | *TmTene2* | 19.91 |
| EGFP *E. coli* | *TmL27a* | 19.49 | EGFP *E. coli* | *TmL27a* | 19.49 |
| EGFP *E. coli* | *TmL27a* | 19.47 | EGFP *E. coli* | *TmL27a* | 19.47 |
| TAK1 *E. coli* | *TmTene1* | 16.39 | TAK1 *E. coli* | *TmTene2* | 22.93 |
| TAK1 *E. coli* | *TmTene1* | 16.45 | TAK1 *E. coli* | *TmTene2* | 22.88 |
| TAK1 *E. coli* | *TmL27a* | 19.66 | TAK1 *E. coli* | *TmL27a* | 19.66 |
| TAK1 *E. coli* | *TmL27a* | 19.76 | TAK1 *E. coli* | *TmL27a* | 19.76 |
| EGFP *S. aureus* | *TmTene1* | 20.93 | EGFP *S. aureus* | *TmTene2* | 27.1 |
| EGFP *S. aureus* | *TmTene1* | 21.01 | EGFP *S. aureus* | *TmTene2* | 27.26 |
| EGFP *S. aureus* | *TmL27a* | 20.14 | EGFP *S. aureus* | *TmL27a* | 20.14 |
| EGFP *S. aureus* | *TmL27a* | 20.26 | EGFP *S. aureus* | *TmL27a* | 20.26 |
| TAK1 *S. aureus* | *TmTene1* | 18.38 | TAK1 *S. aureus* | *TmTene2* | 25.7 |
| TAK1 *S. aureus* | *TmTene1* | 18.36 | TAK1 *S. aureus* | *TmTene2* | 25.76 |
| TAK1 *S. aureus* | *TmL27a* | 18.3 | TAK1 *S. aureus* | *TmL27a* | 18.3 |
| TAK1 *S. aureus* | *TmL27a* | 18.2 | TAK1 *S. aureus* | *TmL27a* | 18.2 |
| EGFP *C. albicans* | *TmTene1* | 20.78 | EGFP *C. albicans* | *TmTene2* | 26.57 |
| EGFP *C. albicans* | *TmTene1* | 20.93 | EGFP *C. albicans* | *TmTene2* | 26.23 |
| EGFP *C. albicans* | *TmL27a* | 18.28 | EGFP *C. albicans* | *TmL27a* | 18.28 |
| EGFP *C. albicans* | *TmL27a* | 18.24 | EGFP *C. albicans* | *TmL27a* | 18.24 |
| TAK1 *C. albicans* | *TmTene1* | 22.23 | TAK1 *C. albicans* | *TmTene2* | 30 |
| TAK1 *C. albicans* | *TmTene1* | 22.21 | TAK1 *C. albicans* | *TmTene2* | 30.4 |
| TAK1 *C. albicans* | *TmL27a* | 19.73 | TAK1 *C. albicans* | *TmL27a* | 19.73 |
| TAK1 *C. albicans* | *TmL27a* | 19.7 | TAK1 *C. albicans* | *TmL27a* | 19.7 |

| Sample name | Gene name | Cq (∆R) | Sample name | Gene name | Cq (∆R) |
| --- | --- | --- | --- | --- | --- |
| EGFP PBS | *TmTene3* | 22.82 | EGFP PBS | *TmTene4* | 24.57 |
| EGFP PBS | *TmTene3* | 22.71 | EGFP PBS | *TmTene4* | 24.64 |
| EGFP PBS | *TmL27a* | 19.33 | EGFP PBS | *TmL27a* | 19.33 |
| EGFP PBS | *TmL27a* | 19.2 | EGFP PBS | *TmL27a* | 19.2 |
| TAK1 PBS | *TmTene3* | 21.47 | TAK1 PBS | *TmTene4* | 20.85 |
| TAK1 PBS | *TmTene3* | 21.24 | TAK1 PBS | *TmTene4* | 21.24 |
| TAK1 PBS | *TmL27a* | 18.55 | TAK1 PBS | *TmL27a* | 18.55 |
| TAK1 PBS | *TmL27a* | 18.34 | TAK1 PBS | *TmL27a* | 18.34 |
| EGFP *E. coli* | *TmTene3* | 24.58 | EGFP *E. coli* | *TmTene4* | 16.17 |
| EGFP *E. coli* | *TmTene3* | 24.69 | EGFP *E. coli* | *TmTene4* | 17.1 |
| EGFP *E. coli* | *TmL27a* | 19.49 | EGFP *E. coli* | *TmL27a* | 19.49 |
| EGFP *E. coli* | *TmL27a* | 19.47 | EGFP *E. coli* | *TmL27a* | 19.47 |
| TAK1 *E. coli* | *TmTene3* | 24.14 | TAK1 *E. coli* | *TmTene4* | 18.18 |
| TAK1 *E. coli* | *TmTene3* | 24.56 | TAK1 *E. coli* | *TmTene4* | 18.28 |
| TAK1 *E. coli* | *TmL27a* | 19.66 | TAK1 *E. coli* | *TmL27a* | 19.66 |
| TAK1 *E. coli* | *TmL27a* | 19.76 | TAK1 *E. coli* | *TmL27a* | 19.76 |
| EGFP *S. aureus* | *TmTene3* | 23.65 | EGFP *S. aureus* | *TmTene4* | 22.66 |
| EGFP *S. aureus* | *TmTene3* | 23.44 | EGFP *S. aureus* | *TmTene4* | 22.96 |
| EGFP *S. aureus* | *TmL27a* | 20.14 | EGFP *S. aureus* | *TmL27a* | 20.14 |
| EGFP *S. aureus* | *TmL27a* | 20.26 | EGFP *S. aureus* | *TmL27a* | 20.26 |
| TAK1 *S. aureus* | *TmTene3* | 21.79 | TAK1 *S. aureus* | *TmTene4* | 19.85 |
| TAK1 *S. aureus* | *TmTene3* | 21.43 | TAK1 *S. aureus* | *TmTene4* | 19.97 |
| TAK1 *S. aureus* | *TmL27a* | 18.3 | TAK1 *S. aureus* | *TmL27a* | 18.3 |
| TAK1 *S. aureus* | *TmL27a* | 18.2 | TAK1 *S. aureus* | *TmL27a* | 18.2 |
| EGFP *C. albicans* | *TmTene3* | 21.37 | EGFP *C. albicans* | *TmTene4* | 21.88 |
| EGFP *C. albicans* | *TmTene3* | 21.41 | EGFP *C. albicans* | *TmTene4* | 22.05 |
| EGFP *C. albicans* | *TmL27a* | 18.28 | EGFP *C. albicans* | *TmL27a* | 18.28 |
| EGFP *C. albicans* | *TmL27a* | 18.24 | EGFP *C. albicans* | *TmL27a* | 18.24 |
| TAK1 *C. albicans* | *TmTene3* | 22.55 | TAK1 *C. albicans* | *TmTene4* | 24.18 |
| TAK1 *C. albicans* | *TmTene3* | 22.6 | TAK1 *C. albicans* | *TmTene4* | 24.38 |
| TAK1 *C. albicans* | *TmL27a* | 19.73 | TAK1 *C. albicans* | *TmL27a* | 19.73 |
| TAK1 *C. albicans* | *TmL27a* | 19.7 | TAK1 *C. albicans* | *TmL27a* | 19.7 |

| Sample name | Gene name | Cq (∆R) | Sample name | Gene name | Cq (∆R) |
| --- | --- | --- | --- | --- | --- |
| EGFP PBS | *TmDef* | 25.74 | EGFP PBS | *TmDef-like* | 24.98 |
| EGFP PBS | *TmDef* | 25.6 | EGFP PBS | *TmDef-like* | 24.77 |
| EGFP PBS | *TmL27a* | 19.07 | EGFP PBS | *TmL27a* | 18.98 |
| EGFP PBS | *TmL27a* | 18.93 | EGFP PBS | *TmL27a* | 18.97 |
| TAK1 PBS | *TmDef* | 27.29 | TAK1 PBS | *TmDef-like* | 21.82 |
| TAK1 PBS | *TmDef* | 26.59 | TAK1 PBS | *TmDef-like* | 21.76 |
| TAK1 PBS | *TmL27a* | 18.68 | TAK1 PBS | *TmL27a* | 18.42 |
| TAK1 PBS | *TmL27a* | 18.1 | TAK1 PBS | *TmL27a* | 18.03 |
| EGFP *E. coli* | *TmDef* | 18.71 | EGFP *E. coli* | *TmDef-like* | 16.92 |
| EGFP *E. coli* | *TmDef* | 18.69 | EGFP *E. coli* | *TmDef-like* | 16.39 |
| EGFP *E. coli* | *TmL27a* | 19.72 | EGFP *E. coli* | *TmL27a* | 19.32 |
| EGFP *E. coli* | *TmL27a* | 19.23 | EGFP *E. coli* | *TmL27a* | 19.23 |
| TAK1 *E. coli* | *TmDef* | 19.21 | TAK1 *E. coli* | *TmDef-like* | 18.58 |
| TAK1 *E. coli* | *TmDef* | 19.2 | TAK1 *E. coli* | *TmDef-like* | 17.99 |
| TAK1 *E. coli* | *TmL27a* | 19.78 | TAK1 *E. coli* | *TmL27a* | 19.52 |
| TAK1 *E. coli* | *TmL27a* | 19.71 | TAK1 *E. coli* | *TmL27a* | 19.3 |
| EGFP *S. aureus* | *TmDef* | 23.42 | EGFP *S. aureus* | *TmDef-like* | 21.73 |
| EGFP *S. aureus* | *TmDef* | 23.57 | EGFP *S. aureus* | *TmDef-like* | 21.66 |
| EGFP *S. aureus* | *TmL27a* | 20 | EGFP *S. aureus* | *TmL27a* | 19.96 |
| EGFP *S. aureus* | *TmL27a* | 19.93 | EGFP *S. aureus* | *TmL27a* | 19.89 |
| TAK1 *S. aureus* | *TmDef* | 20.48 | TAK1 *S. aureus* | *TmDef-like* | 19 |
| TAK1 *S. aureus* | *TmDef* | 20.83 | TAK1 *S. aureus* | *TmDef-like* | 18.93 |
| TAK1 *S. aureus* | *TmL27a* | 17.96 | TAK1 *S. aureus* | *TmL27a* | 18.26 |
| TAK1 *S. aureus* | *TmL27a* | 17.92 | TAK1 *S. aureus* | *TmL27a* | 17.98 |
| EGFP *C. albicans* | *TmDef* | 21.79 | EGFP *C. albicans* | *TmDef-like* | 21.88 |
| EGFP *C. albicans* | *TmDef* | 21.94 | EGFP *C. albicans* | *TmDef-like* | 21.96 |
| EGFP *C. albicans* | *TmL27a* | 17.89 | EGFP *C. albicans* | *TmL27a* | 17.95 |
| EGFP *C. albicans* | *TmL27a* | 17.82 | EGFP *C. albicans* | *TmL27a* | 17.86 |
| TAK1 *C. albicans* | *TmDef* | 23.81 | TAK1 *C. albicans* | *TmDef-like* | 23.02 |
| TAK1 *C. albicans* | *TmDef* | 23.77 | TAK1 *C. albicans* | *TmDef-like* | 22.96 |
| TAK1 *C. albicans* | *TmL27a* | 19.64 | TAK1 *C. albicans* | *TmL27a* | 19.29 |
| TAK1 *C. albicans* | *TmL27a* | 19.62 | TAK1 *C. albicans* | *TmL27a* | 19.18 |

| Sample name | *Gene name* | Cq (∆R) | Sample name | *Gene name* | Cq (∆R) |
| --- | --- | --- | --- | --- | --- |
| EGFP PBS | *TmColeA* | 27.26 | EGFP PBS | *TmColeB* | 31.16 |
| EGFP PBS | *TmColeA* | 26.96 | EGFP PBS | *TmColeB* | 30.69 |
| EGFP PBS | *TmL27a* | 18.98 | EGFP PBS | *TmL27a* | 18.98 |
| EGFP PBS | *TmL27a* | 18.97 | EGFP PBS | *TmL27a* | 18.97 |
| TAK1 PBS | *TmColeA* | 22.84 | TAK1 PBS | *TmColeB* | 27.17 |
| TAK1 PBS | *TmColeA* | 22.1 | TAK1 PBS | *TmColeB* | 27.07 |
| TAK1 PBS | *TmL27a* | 18.42 | TAK1 PBS | *TmL27a* | 18.42 |
| TAK1 PBS | *TmL27a* | 18.03 | TAK1 PBS | *TmL27a* | 18.03 |
| EGFP *E. coli* | *TmColeA* | 19.16 | EGFP *E. coli* | *TmColeB* | 22.2 |
| EGFP *E. coli* | *TmColeA* | 19.03 | EGFP *E. coli* | *TmColeB* | 21.83 |
| EGFP *E. coli* | *TmL27a* | 19.32 | EGFP *E. coli* | *TmL27a* | 19.32 |
| EGFP *E. coli* | *TmL27a* | 19.23 | EGFP *E. coli* | *TmL27a* | 19.23 |
| TAK1 *E. coli* | *TmColeA* | 20.98 | TAK1 *E. coli* | *TmColeB* | 23.8 |
| TAK1 *E. coli* | *TmColeA* | 21.01 | TAK1 *E. coli* | *TmColeB* | 23.51 |
| TAK1 *E. coli* | *TmL27a* | 19.52 | TAK1 *E. coli* | *TmL27a* | 19.52 |
| TAK1 *E. coli* | *TmL27a* | 19.3 | TAK1 *E. coli* | *TmL27a* | 19.3 |
| EGFP *S. aureus* | *TmColeA* | 22.68 | EGFP *S. aureus* | *TmColeB* | 28.54 |
| EGFP *S. aureus* | *TmColeA* | 22.71 | EGFP *S. aureus* | *TmColeB* | 28.98 |
| EGFP *S. aureus* | *TmL27a* | 19.96 | EGFP *S. aureus* | *TmL27a* | 19.96 |
| EGFP *S. aureus* | *TmL27a* | 19.89 | EGFP *S. aureus* | *TmL27a* | 19.89 |
| TAK1 *S. aureus* | *TmColeA* | 20.92 | TAK1 *S. aureus* | *TmColeB* | 26.12 |
| TAK1 *S. aureus* | *TmColeA* | 20.57 | TAK1 *S. aureus* | *TmColeB* | 25.86 |
| TAK1 *S. aureus* | *TmL27a* | 18.26 | TAK1 *S. aureus* | *TmL27a* | 18.26 |
| TAK1 *S. aureus* | *TmL27a* | 17.98 | TAK1 *S. aureus* | *TmL27a* | 17.98 |
| EGFP *C. albicans* | *TmColeA* | 23.48 | EGFP *C. albicans* | *TmColeB* | 28.46 |
| EGFP *C. albicans* | *TmColeA* | 23.11 | EGFP *C. albicans* | *TmColeB* | 27.94 |
| EGFP *C. albicans* | *TmL27a* | 17.95 | EGFP *C. albicans* | *TmL27a* | 17.95 |
| EGFP *C. albicans* | *TmL27a* | 17.86 | EGFP *C. albicans* | *TmL27a* | 17.86 |
| TAK1 *C. albicans* | *TmColeA* | 25.5 | TAK1 *C. albicans* | *TmColeB* | 31.24 |
| TAK1 *C. albicans* | *TmColeA* | 25.29 | TAK1 *C. albicans* | *TmColeB* | 30.69 |
| TAK1 *C. albicans* | *TmL27a* | 19.29 | TAK1 *C. albicans* | *TmL27a* | 19.29 |
| TAK1 *C. albicans* | *TmL27a* | 19.18 | TAK1 *C. albicans* | *TmL27a* | 19.18 |

| Sample name | Gene name | Cq (∆R) | Sample name | Gene name | Cq (∆R) |
| --- | --- | --- | --- | --- | --- |
| EGFP PBS | *TmColeC* | 27.5 | EGFP PBS | *TmCec2* | 20.85 |
| EGFP PBS | *TmColeC* | 27.73 | EGFP PBS | *TmCec2* | 20.73 |
| EGFP PBS | *TmL27a* | 18.98 | EGFP PBS | *TmL27a* | 18.98 |
| EGFP PBS | *TmL27a* | 18.97 | EGFP PBS | *TmL27a* | 18.97 |
| TAK1 PBS | *TmColeC* | 22.84 | TAK1 PBS | *TmCec2* | 18.95 |
| TAK1 PBS | *TmColeC* | 23.05 | TAK1 PBS | *TmCec2* | 19 |
| TAK1 PBS | *TmL27a* | 18.42 | TAK1 PBS | *TmL27a* | 18.42 |
| TAK1 PBS | *TmL27a* | 18.03 | TAK1 PBS | *TmL27a* | 18.03 |
| EGFP *E. coli* | *TmColeC* | 20.69 | EGFP *E. coli* | *TmCec2* | 19.88 |
| EGFP *E. coli* | *TmColeC* | 20.35 | EGFP *E. coli* | *TmCec2* | 19.94 |
| EGFP *E. coli* | *TmL27a* | 19.32 | EGFP *E. coli* | *TmL27a* | 19.32 |
| EGFP *E. coli* | *TmL27a* | 19.23 | EGFP *E. coli* | *TmL27a* | 19.23 |
| TAK1 *E. coli* | *TmColeC* | 21.96 | TAK1 *E. coli* | *TmCec2* | 20.58 |
| TAK1 *E. coli* | *TmColeC* | 21.98 | TAK1 *E. coli* | *TmCec2* | 20.12 |
| TAK1 *E. coli* | *TmL27a* | 19.52 | TAK1 *E. coli* | *TmL27a* | 19.52 |
| TAK1 *E. coli* | *TmL27a* | 19.3 | TAK1 *E. coli* | *TmL27a* | 19.3 |
| EGFP *S. aureus* | *TmColeC* | 25.08 | EGFP *S. aureus* | *TmCec2* | 21.62 |
| EGFP *S. aureus* | *TmColeC* | 25.09 | EGFP *S. aureus* | *TmCec2* | 20.73 |
| EGFP *S. aureus* | *TmL27a* | 19.96 | EGFP *S. aureus* | *TmL27a* | 19.96 |
| EGFP *S. aureus* | *TmL27a* | 19.89 | EGFP *S. aureus* | *TmL27a* | 19.89 |
| TAK1 *S. aureus* | *TmColeC* | 21.86 | TAK1 *S. aureus* | *TmCec2* | 20.6 |
| TAK1 *S. aureus* | *TmColeC* | 21.9 | TAK1 *S. aureus* | *TmCec2* | 19.73 |
| TAK1 *S. aureus* | *TmL27a* | 18.26 | TAK1 *S. aureus* | *TmL27a* | 18.26 |
| TAK1 *S. aureus* | *TmL27a* | 17.98 | TAK1 *S. aureus* | *TmL27a* | 17.98 |
| EGFP *C. albicans* | *TmColeC* | 23.93 | EGFP *C. albicans* | *TmCec2* | 18.81 |
| EGFP *C. albicans* | *TmColeC* | 24.1 | EGFP *C. albicans* | *TmCec2* | 19.04 |
| EGFP *C. albicans* | *TmL27a* | 17.95 | EGFP *C. albicans* | *TmL27a* | 17.95 |
| EGFP *C. albicans* | *TmL27a* | 17.86 | EGFP *C. albicans* | *TmL27a* | 17.86 |
| TAK1 *C. albicans* | *TmColeC* | 26.61 | TAK1 *C. albicans* | *TmCec2* | 19.88 |
| TAK1 *C. albicans* | *TmColeC* | 27.16 | TAK1 *C. albicans* | *TmCec2* | 19.92 |
| TAK1 *C. albicans* | *TmL27a* | 19.29 | TAK1 *C. albicans* | *TmL27a* | 19.29 |
| TAK1 *C. albicans* | *TmL27a* | 19.18 | TAK1 *C. albicans* | *TmL27a* | 19.18 |

| Sample name | Gene name | Cq (∆R) | Sample name | Gene name | Cq (∆R) |
| --- | --- | --- | --- | --- | --- |
| EGFP PBS | *TmAtta1a* | 26.64 | EGFP PBS | *TmAtta1b* | 25.86 |
| EGFP PBS | *TmAtta1a* | 26.98 | EGFP PBS | *TmAtta1b* | 25.67 |
| EGFP PBS | *TmL27a* | 19.53 | EGFP PBS | *TmL27a* | 17.3 |
| EGFP PBS | *TmL27a* | 19.52 | EGFP PBS | *TmL27a* | 17.26 |
| TAK1 PBS | *TmAtta1a* | 22.14 | TAK1 PBS | *TmAtta1b* | 22.94 |
| TAK1 PBS | *TmAtta1a* | 22.49 | TAK1 PBS | *TmAtta1b* | 22.92 |
| TAK1 PBS | *TmL27a* | 18.6 | TAK1 PBS | *TmL27a* | 18.6 |
| TAK1 PBS | *TmL27a* | 18.63 | TAK1 PBS | *TmL27a* | 18.63 |
| EGFP *E. coli* | *TmAtta1a* | 21.21 | EGFP *E. coli* | *TmAtta1b* | 17.75 |
| EGFP *E. coli* | *TmAtta1a* | 20.97 | EGFP *E. coli* | *TmAtta1b* | 17.68 |
| EGFP *E. coli* | *TmL27a* | 19.63 | EGFP *E. coli* | *TmL27a* | 19.63 |
| EGFP *E. coli* | *TmL27a* | 19.58 | EGFP *E. coli* | *TmL27a* | 19.58 |
| TAK1 *E. coli* | *TmAtta1a* | 22.54 | TAK1 *E. coli* | *TmAtta1b* | 19.5 |
| TAK1 *E. coli* | *TmAtta1a* | 22.36 | TAK1 *E. coli* | *TmAtta1b* | 19.48 |
| TAK1 *E. coli* | *TmL27a* | 19.73 | TAK1 *E. coli* | *TmL27a* | 19.73 |
| TAK1 *E. coli* | *TmL27a* | 19.7 | TAK1 *E. coli* | *TmL27a* | 19.7 |
| EGFP *S. aureus* | *TmAtta1a* | 24.83 | EGFP *S. aureus* | *TmAtta1b* | 23.91 |
| EGFP *S. aureus* | *TmAtta1a* | 24.8 | EGFP *S. aureus* | *TmAtta1b* | 23.68 |
| EGFP *S. aureus* | *TmL27a* | 20.41 | EGFP *S. aureus* | *TmL27a* | 20.41 |
| EGFP *S. aureus* | *TmL27a* | 20.44 | EGFP *S. aureus* | *TmL27a* | 20.44 |
| TAK1 *S. aureus* | *TmAtta1a* | 21.31 | TAK1 *S. aureus* | *TmAtta1b* | 21.02 |
| TAK1 *S. aureus* | *TmAtta1a* | 21.3 | TAK1 *S. aureus* | *TmAtta1b* | 20.85 |
| TAK1 *S. aureus* | *TmL27a* | 18.5 | TAK1 *S. aureus* | *TmL27a* | 18.5 |
| TAK1 *S. aureus* | *TmL27a* | 18.39 | TAK1 *S. aureus* | *TmL27a* | 18.39 |
| EGFP *C. albicans* | *TmAtta1a* | 23.82 | EGFP *C. albicans* | *TmAtta1b* | 22 |
| EGFP *C. albicans* | *TmAtta1a* | 23.88 | EGFP *C. albicans* | *TmAtta1b* | 22.1 |
| EGFP *C. albicans* | *TmL27a* | 18.46 | EGFP *C. albicans* | *TmL27a* | 16.84 |
| EGFP *C. albicans* | *TmL27a* | 18.24 | EGFP *C. albicans* | *TmL27a* | 16.55 |
| TAK1 *C. albicans* | *TmAtta1a* | 26.49 | TAK1 *C. albicans* | *TmAtta1b* | 25.2 |
| TAK1 *C. albicans* | *TmAtta1a* | 26.17 | TAK1 *C. albicans* | *TmAtta1b* | 24.99 |
| TAK1 *C. albicans* | *TmL27a* | 19.93 | TAK1 *C. albicans* | *TmL27a* | 19.93 |
| TAK1 *C. albicans* | *TmL27a* | 19.93 | TAK1 *C. albicans* | *TmL27a* | 19.93 |

| Sample name | Gene name | Cq (∆R) | Sample name | Gene name | Cq (∆R) |
| --- | --- | --- | --- | --- | --- |
| EGFP PBS | *TmAtta2* | 25.91 | EGFP PBS | *TmTLP1* | 27.44 |
| EGFP PBS | *TmAtta2* | 25.79 | EGFP PBS | *TmTLP1* | 28.4 |
| EGFP PBS | *TmL27a* | 19.53 | EGFP PBS | *TmL27a* | 19.53 |
| EGFP PBS | *TmL27a* | 19.52 | EGFP PBS | *TmL27a* | 19.52 |
| TAK1 PBS | *TmAtta2* | 22.85 | TAK1 PBS | *TmTLP1* | 23.67 |
| TAK1 PBS | *TmAtta2* | 22.75 | TAK1 PBS | *TmTLP1* | 24.83 |
| TAK1 PBS | *TmL27a* | 18.6 | TAK1 PBS | *TmL27a* | 18.6 |
| TAK1 PBS | *TmL27a* | 18.63 | TAK1 PBS | *TmL27a* | 18.63 |
| EGFP *E. coli* | *TmAtta2* | 16.48 | EGFP *E. coli* | *TmTLP1* | 27.23 |
| EGFP *E. coli* | *TmAtta2* | 16.84 | EGFP *E. coli* | *TmTLP1* | 27.29 |
| EGFP *E. coli* | *TmL27a* | 19.63 | EGFP *E. coli* | *TmL27a* | 19.63 |
| EGFP *E. coli* | *TmL27a* | 19.58 | EGFP *E. coli* | *TmL27a* | 19.58 |
| TAK1 *E. coli* | *TmAtta2* | 18.46 | TAK1 *E. coli* | *TmTLP1* | 25.42 |
| TAK1 *E. coli* | *TmAtta2* | 18.66 | TAK1 *E. coli* | *TmTLP1* | 25.64 |
| TAK1 *E. coli* | *TmL27a* | 19.73 | TAK1 *E. coli* | *TmL27a* | 19.73 |
| TAK1 *E. coli* | *TmL27a* | 19.7 | TAK1 *E. coli* | *TmL27a* | 19.7 |
| EGFP *S. aureus* | *TmAtta2* | 22.35 | EGFP *S. aureus* | *TmTLP1* | 28.39 |
| EGFP *S. aureus* | *TmAtta2* | 22.71 | EGFP *S. aureus* | *TmTLP1* | 28.87 |
| EGFP *S. aureus* | *TmL27a* | 20.41 | EGFP *S. aureus* | *TmL27a* | 20.41 |
| EGFP *S. aureus* | *TmL27a* | 20.44 | EGFP *S. aureus* | *TmL27a* | 20.44 |
| TAK1 *S. aureus* | *TmAtta2* | 20.7 | TAK1 *S. aureus* | *TmTLP1* | 24.16 |
| TAK1 *S. aureus* | *TmAtta2* | 20.77 | TAK1 *S. aureus* | *TmTLP1* | 24.22 |
| TAK1 *S. aureus* | *TmL27a* | 18.5 | TAK1 *S. aureus* | *TmL27a* | 18.5 |
| TAK1 *S. aureus* | *TmL27a* | 18.39 | TAK1 *S. aureus* | *TmL27a* | 18.39 |
| EGFP *C. albicans* | *TmAtta2* | 22.94 | EGFP *C. albicans* | *TmTLP1* | 26.26 |
| EGFP *C. albicans* | *TmAtta2* | 22.88 | EGFP *C. albicans* | *TmTLP1* | 26.1 |
| EGFP *C. albicans* | *TmL27a* | 18.46 | EGFP *C. albicans* | *TmL27a* | 18.46 |
| EGFP *C. albicans* | *TmL27a* | 18.24 | EGFP *C. albicans* | *TmL27a* | 18.24 |
| TAK1 *C. albicans* | *TmAtta2* | 25.71 | TAK1 *C. albicans* | *TmTLP1* | 28.56 |
| TAK1 *C. albicans* | *TmAtta2* | 25.89 | TAK1 *C. albicans* | *TmTLP1* | 29.13 |
| TAK1 *C. albicans* | *TmL27a* | 19.93 | TAK1 *C. albicans* | *TmL27a* | 19.93 |
| TAK1 *C. albicans* | *TmL27a* | 19.93 | TAK1 *C. albicans* | *TmL27a* | 19.93 |

| Sample name | Gene name | Cq (∆R) |
| --- | --- | --- |
| EGFP PBS | *TmTLP2* | 20.1 |
| EGFP PBS | *TmTLP2* | 19.96 |
| EGFP PBS | *TmL27a* | 19.53 |
| EGFP PBS | *TmL27a* | 19.52 |
| TAK1 PBS | *TmTLP2* | 19.9 |
| TAK1 PBS | *TmTLP2* | 19.76 |
| TAK1 PBS | *TmL27a* | 18.6 |
| TAK1 PBS | *TmL27a* | 18.63 |
| EGFP *E. coli* | *TmTLP2* | 20.67 |
| EGFP *E. coli* | *TmTLP2* | 20.84 |
| EGFP *E. coli* | *TmL27a* | 19.63 |
| EGFP *E. coli* | *TmL27a* | 19.58 |
| TAK1 *E. coli* | *TmTLP2* | 20.71 |
| TAK1 *E. coli* | *TmTLP2* | 20.74 |
| TAK1 *E. coli* | *TmL27a* | 19.73 |
| TAK1 *E. coli* | *TmL27a* | 19.7 |
| EGFP *S. aureus* | *TmTLP2* | 20.72 |
| EGFP *S. aureus* | *TmTLP2* | 20.91 |
| EGFP *S. aureus* | *TmL27a* | 20.41 |
| EGFP *S. aureus* | *TmL27a* | 20.44 |
| TAK1 *S. aureus* | *TmTLP2* | 19.14 |
| TAK1 *S. aureus* | *TmTLP2* | 18.91 |
| TAK1 *S. aureus* | *TmL27a* | 18.5 |
| TAK1 *S. aureus* | *TmL27a* | 18.39 |
| EGFP *C. albicans* | *TmTLP2* | 19.13 |
| EGFP *C. albicans* | *TmTLP2* | 19.25 |
| EGFP *C. albicans* | *TmL27a* | 18.46 |
| EGFP *C. albicans* | *TmL27a* | 18.24 |
| TAK1 *C. albicans* | *TmTLP2* | 20.6 |
| TAK1 *C. albicans* | *TmTLP2* | 20.81 |
| TAK1 *C. albicans* | *TmL27a* | 19.93 |
| TAK1 *C. albicans* | *TmL27a* | 19.93 |

**Supplementary Table11. Raw data of qPCR in AMP expression patterns.**

Cq data of *TmTak1* and *TmL27a* in *T. molitor* at integument(IT).

| Sample name | Gene name | Cq (∆R) | Sample name | Gene name | Cq (∆R) |
| --- | --- | --- | --- | --- | --- |
| EGFP PBS | *TmTene1* | 21.57 | EGFP PBS | *TmTene2* | 31.35 |
| EGFP PBS | *TmTene1* | 21.05 | EGFP PBS | *TmTene2* | 30.63 |
| EGFP PBS | *TmL27a* | 19.92 | EGFP PBS | *TmL27a* | 19.92 |
| EGFP PBS | *TmL27a* | 19.88 | EGFP PBS | *TmL27a* | 19.88 |
| TAK1 PBS | *TmTene1* | 22.56 | TAK1 PBS | *TmTene2* | 31.98 |
| TAK1 PBS | *TmTene1* | 21.93 | TAK1 PBS | *TmTene2* | 30.74 |
| TAK1 PBS | *TmL27a* | 19.91 | TAK1 PBS | *TmL27a* | 19.91 |
| TAK1 PBS | *TmL27a* | 19.79 | TAK1 PBS | *TmL27a* | 19.79 |
| EGFP *E. coli* | *TmTene1* | 15.05 | EGFP *E. coli* | *TmTene2* | 20.31 |
| EGFP *E. coli* | *TmTene1* | 14.9 | EGFP *E. coli* | *TmTene2* | 20.21 |
| EGFP *E. coli* | *TmL27a* | 19.28 | EGFP *E. coli* | *TmL27a* | 19.28 |
| EGFP *E. coli* | *TmL27a* | 19.18 | EGFP *E. coli* | *TmL27a* | 19.18 |
| TAK1 *E. coli* | *TmTene1* | 16.14 | TAK1 *E. coli* | *TmTene2* | 24.54 |
| TAK1 *E. coli* | *TmTene1* | 15.97 | TAK1 *E. coli* | *TmTene2* | 24.07 |
| TAK1 *E. coli* | *TmL27a* | 19.27 | TAK1 *E. coli* | *TmL27a* | 19.73 |
| TAK1 *E. coli* | *TmL27a* | 19.73 | TAK1 *E. coli* | *TmL27a* | 19.27 |
| EGFP *S. aureus* | *TmTene1* | 18.02 | EGFP *S. aureus* | *TmTene2* | 23.52 |
| EGFP *S. aureus* | *TmTene1* | 17.98 | EGFP *S. aureus* | *TmTene2* | 23.07 |
| EGFP *S. aureus* | *TmL27a* | 20.22 | EGFP *S. aureus* | *TmL27a* | 20.22 |
| EGFP *S. aureus* | *TmL27a* | 19.98 | EGFP *S. aureus* | *TmL27a* | 19.98 |
| TAK1 *S. aureus* | *TmTene1* | 17.78 | TAK1 *S. aureus* | *TmTene2* | 25.63 |
| TAK1 *S. aureus* | *TmTene1* | 17.88 | TAK1 *S. aureus* | *TmTene2* | 24.86 |
| TAK1 *S. aureus* | *TmL27a* | 20.08 | TAK1 *S. aureus* | *TmL27a* | 20.08 |
| TAK1 *S. aureus* | *TmL27a* | 19.89 | TAK1 *S. aureus* | *TmL27a* | 19.89 |
| EGFP *C. albicans* | *TmTene1* | 19.98 | EGFP *C. albicans* | *TmTene2* | 25.88 |
| EGFP *C. albicans* | *TmTene1* | 19.82 | EGFP *C. albicans* | *TmTene2* | 25.6 |
| EGFP *C. albicans* | *TmL27a* | 19.99 | EGFP *C. albicans* | *TmL27a* | 19.99 |
| EGFP *C. albicans* | *TmL27a* | 19.7 | EGFP *C. albicans* | *TmL27a* | 19.7 |
| TAK1 *C. albicans* | *TmTene1* | 19.99 | TAK1 *C. albicans* | *TmTene2* | 29.37 |
| TAK1 *C. albicans* | *TmTene1* | 19.96 | TAK1 *C. albicans* | *TmTene2* | 28.99 |
| TAK1 *C. albicans* | *TmL27a* | 19.59 | TAK1 *C. albicans* | *TmL27a* | 19.59 |
| TAK1 *C. albicans* | *TmL27a* | 19.23 | TAK1 *C. albicans* | *TmL27a* | 19.23 |

| Sample name | Gene name | Cq (∆R) | Sample name | Gene name | Cq (∆R) |
| --- | --- | --- | --- | --- | --- |
| EGFP PBS | *TmTene3* | 19.37 | EGFP PBS | *TmTene4* | 23.93 |
| EGFP PBS | *TmTene3* | 19.22 | EGFP PBS | *TmTene4* | 24.01 |
| EGFP PBS | *TmL27a* | 19.92 | EGFP PBS | *TmL27a* | 19.92 |
| EGFP PBS | *TmL27a* | 19.88 | EGFP PBS | *TmL27a* | 19.88 |
| TAK1 PBS | *TmTene3* | 20.1 | TAK1 PBS | *TmTene4* | 26.3 |
| TAK1 PBS | *TmTene3* | 19.87 | TAK1 PBS | *TmTene4* | 25.96 |
| TAK1 PBS | *TmL27a* | 19.91 | TAK1 PBS | *TmL27a* | 19.91 |
| TAK1 PBS | *TmL27a* | 19.79 | TAK1 PBS | *TmL27a* | 19.79 |
| EGFP *E. coli* | *TmTene3* | 21.07 | EGFP *E. coli* | *TmTene4* | 16.79 |
| EGFP *E. coli* | *TmTene3* | 20.93 | EGFP *E. coli* | *TmTene4* | 16.49 |
| EGFP *E. coli* | *TmL27a* | 19.28 | EGFP *E. coli* | *TmL27a* | 19.28 |
| EGFP *E. coli* | *TmL27a* | 19.18 | EGFP *E. coli* | *TmL27a* | 19.18 |
| TAK1 *E. coli* | *TmTene3* | 20.37 | TAK1 *E. coli* | *TmTene4* | 18.13 |
| TAK1 *E. coli* | *TmTene3* | 20.14 | TAK1 *E. coli* | *TmTene4* | 17.9 |
| TAK1 *E. coli* | *TmL27a* | 19.73 | TAK1 *E. coli* | *TmL27a* | 19.73 |
| TAK1 *E. coli* | *TmL27a* | 19.27 | TAK1 *E. coli* | *TmL27a* | 19.27 |
| EGFP *S. aureus* | *TmTene3* | 20.72 | EGFP *S. aureus* | *TmTene4* | 19.98 |
| EGFP *S. aureus* | *TmTene3* | 20.57 | EGFP *S. aureus* | *TmTene4* | 19.89 |
| EGFP *S. aureus* | *TmL27a* | 20.22 | EGFP *S. aureus* | *TmL27a* | 20.22 |
| EGFP *S. aureus* | *TmL27a* | 19.98 | EGFP *S. aureus* | *TmL27a* | 19.98 |
| TAK1 *S. aureus* | *TmTene3* | 20.85 | TAK1 *S. aureus* | *TmTene4* | 20.57 |
| TAK1 *S. aureus* | *TmTene3* | 20.73 | TAK1 *S. aureus* | *TmTene4* | 20.21 |
| TAK1 *S. aureus* | *TmL27a* | 20.08 | TAK1 *S. aureus* | *TmL27a* | 20.08 |
| TAK1 *S. aureus* | *TmL27a* | 19.89 | TAK1 *S. aureus* | *TmL27a* | 19.89 |
| EGFP *C. albicans* | *TmTene3* | 20 | EGFP *C. albicans* | *TmTene4* | 23.64 |
| EGFP *C. albicans* | *TmTene3* | 20.07 | EGFP *C. albicans* | *TmTene4* | 23.75 |
| EGFP *C. albicans* | *TmL27a* | 19.99 | EGFP *C. albicans* | *TmL27a* | 19.99 |
| EGFP *C. albicans* | *TmL27a* | 19.7 | EGFP *C. albicans* | *TmL27a* | 19.7 |
| TAK1 *C. albicans* | *TmTene3* | 20.64 | TAK1 *C. albicans* | *TmTene4* | 23.71 |
| TAK1 *C. albicans* | *TmTene3* | 20.08 | TAK1 *C. albicans* | *TmTene4* | 23.59 |
| TAK1 *C. albicans* | *TmL27a* | 19.59 | TAK1 *C. albicans* | *TmL27a* | 19.59 |
| TAK1 *C. albicans* | *TmL27a* | 19.23 | TAK1 *C. albicans* | *TmL27a* | 19.23 |

| Sample name | Gene name | Cq (∆R) | Sample name | Gene name | Cq (∆R) |
| --- | --- | --- | --- | --- | --- |
| EGFP PBS | *TmDef* | 24.38 | EGFP PBS | *TmDef-like* | 22.99 |
| EGFP PBS | *TmDef* | 23.98 | EGFP PBS | *TmDef-like* | 22.94 |
| EGFP PBS | *TmL27a* | 19.92 | EGFP PBS | *TmL27a* | 20.35 |
| EGFP PBS | *TmL27a* | 19.88 | EGFP PBS | *TmL27a* | 20.22 |
| TAK1 PBS | *TmDef* | 24.44 | TAK1 PBS | *TmDef-like* | 24.84 |
| TAK1 PBS | *TmDef* | 24.86 | TAK1 PBS | *TmDef-like* | 24.04 |
| TAK1 PBS | *TmL27a* | 19.91 | TAK1 PBS | *TmL27a* | 20.37 |
| TAK1 PBS | *TmL27a* | 19.79 | TAK1 PBS | *TmL27a* | 20.2 |
| EGFP *E. coli* | *TmDef* | 17.17 | EGFP *E. coli* | *TmDef-like* | 16.5 |
| EGFP *E. coli* | *TmDef* | 17.02 | EGFP *E. coli* | *TmDef-like* | 16.48 |
| EGFP *E. coli* | *TmL27a* | 19.28 | EGFP *E. coli* | *TmL27a* | 19.45 |
| EGFP *E. coli* | *TmL27a* | 19.18 | EGFP *E. coli* | *TmL27a* | 19.35 |
| TAK1 *E. coli* | *TmDef* | 17.86 | TAK1 *E. coli* | *TmDef-like* | 17.21 |
| TAK1 *E. coli* | *TmDef* | 17.74 | TAK1 *E. coli* | *TmDef-like* | 17.08 |
| TAK1 *E. coli* | *TmL27a* | 19.73 | TAK1 *E. coli* | *TmL27a* | 20.01 |
| TAK1 *E. coli* | *TmL27a* | 19.27 | TAK1 *E. coli* | *TmL27a* | 19.54 |
| EGFP *S. aureus* | *TmDef* | 18.93 | EGFP *S. aureus* | *TmDef-like* | 19.1 |
| EGFP *S. aureus* | *TmDef* | 18.93 | EGFP *S. aureus* | *TmDef-like* | 19.42 |
| EGFP *S. aureus* | *TmL27a* | 20.22 | EGFP *S. aureus* | *TmL27a* | 20.53 |
| EGFP *S. aureus* | *TmL27a* | 19.98 | EGFP *S. aureus* | *TmL27a* | 20.6 |
| TAK1 *S. aureus* | *TmDef* | 19.56 | TAK1 *S. aureus* | *TmDef-like* | 18.92 |
| TAK1 *S. aureus* | *TmDef* | 19 | TAK1 *S. aureus* | *TmDef-like* | 18.62 |
| TAK1 *S. aureus* | *TmL27a* | 20.08 | TAK1 *S. aureus* | *TmL27a* | 20.25 |
| TAK1 *S. aureus* | *TmL27a* | 19.89 | TAK1 *S. aureus* | *TmL27a* | 20.24 |
| EGFP *C. albicans* | *TmDef* | 21.65 | EGFP *C. albicans* | *TmDef-like* | 21.9 |
| EGFP *C. albicans* | *TmDef* | 20.99 | EGFP *C. albicans* | *TmDef-like* | 27.6 |
| EGFP *C. albicans* | *TmL27a* | 19.99 | EGFP *C. albicans* | *TmL27a* | 20.37 |
| EGFP *C. albicans* | *TmL27a* | 19.7 | EGFP *C. albicans* | *TmL27a* | 20.35 |
| TAK1 *C. albicans* | *TmDef* | 20.64 | TAK1 *C. albicans* | *TmDef-like* | 21.56 |
| TAK1 *C. albicans* | *TmDef* | 20.36 | TAK1 *C. albicans* | *TmDef-like* | 21.52 |
| TAK1 *C. albicans* | *TmL27a* | 19.59 | TAK1 *C. albicans* | *TmL27a* | 19.91 |
| TAK1 *C. albicans* | *TmL27a* | 19.23 | TAK1 *C. albicans* | *TmL27a* | 19.76 |

| Sample name | Gene name | Cq (∆R) | Sample name | Gene name | Cq (∆R) |
| --- | --- | --- | --- | --- | --- |
| EGFP PBS | *TmColeA* | 24.71 | EGFP PBS | *TmColeB* | 33.04 |
| EGFP PBS | *TmColeA* | 24.9 | EGFP PBS | *TmColeB* | 32.78 |
| EGFP PBS | *TmL27a* | 20.35 | EGFP PBS | *TmL27a* | 21.45 |
| EGFP PBS | *TmL27a* | 20.22 | EGFP PBS | *TmL27a* | 21.14 |
| TAK1 PBS | *TmColeA* | 25.85 | TAK1 PBS | *TmColeB* | 33.88 |
| TAK1 PBS | *TmColeA* | 25.92 | TAK1 PBS | *TmColeB* | 33.1 |
| TAK1 PBS | *TmL27a* | 20.37 | TAK1 PBS | *TmL27a* | 21.12 |
| TAK1 PBS | *TmL27a* | 20.2 | TAK1 PBS | *TmL27a* | 21.24 |
| EGFP *E. coli* | *TmColeA* | 17.03 | EGFP *E. coli* | *TmColeB* | 24.44 |
| EGFP *E. coli* | *TmColeA* | 17.32 | EGFP *E. coli* | *TmColeB* | 24.48 |
| EGFP *E. coli* | *TmL27a* | 19.45 | EGFP *E. coli* | *TmL27a* | 20.22 |
| EGFP *E. coli* | *TmL27a* | 19.35 | EGFP *E. coli* | *TmL27a* | 20.15 |
| TAK1 *E. coli* | *TmColeA* | 18.94 | TAK1 *E. coli* | *TmColeB* | 26.51 |
| TAK1 *E. coli* | *TmColeA* | 18.97 | TAK1 *E. coli* | *TmColeB* | 26.21 |
| TAK1 *E. coli* | *TmL27a* | 20.01 | TAK1 *E. coli* | *TmL27a* | 20.72 |
| TAK1 *E. coli* | *TmL27a* | 19.54 | TAK1 *E. coli* | *TmL27a* | 20.53 |
| EGFP *S. aureus* | *TmColeA* | 19.52 | EGFP *S. aureus* | *TmColeB* | 27.86 |
| EGFP *S. aureus* | *TmColeA* | 19.55 | EGFP *S. aureus* | *TmColeB* | 28.02 |
| EGFP *S. aureus* | *TmL27a* | 20.53 | EGFP *S. aureus* | *TmL27a* | 21.09 |
| EGFP *S. aureus* | *TmL27a* | 20.6 | EGFP *S. aureus* | *TmL27a* | 21.26 |
| TAK1 *S. aureus* | *TmColeA* | 20.03 | TAK1 *S. aureus* | *TmColeB* | 28.47 |
| TAK1 *S. aureus* | *TmColeA* | 19.99 | TAK1 *S. aureus* | *TmColeB* | 28.57 |
| TAK1 *S. aureus* | *TmL27a* | 20.25 | TAK1 *S. aureus* | *TmL27a* | 21.07 |
| TAK1 *S. aureus* | *TmL27a* | 20.24 | TAK1 *S. aureus* | *TmL27a* | 21.05 |
| EGFP *C. albicans* | *TmColeA* | 23.54 | EGFP *C. albicans* | *TmColeB* | 31.28 |
| EGFP *C. albicans* | *TmColeA* | 23.53 | EGFP *C. albicans* | *TmColeB* | 31.22 |
| EGFP *C. albicans* | *TmL27a* | 20.37 | EGFP *C. albicans* | *TmL27a* | 20.79 |
| EGFP *C. albicans* | *TmL27a* | 20.35 | EGFP *C. albicans* | *TmL27a* | 21.12 |
| TAK1 *C. albicans* | *TmColeA* | 23.99 | TAK1 *C. albicans* | *TmColeB* | 32.03 |
| TAK1 *C. albicans* | *TmColeA* | 24.26 | TAK1 *C. albicans* | *TmColeB* | 32.24 |
| TAK1 *C. albicans* | *TmL27a* | 19.91 | TAK1 *C. albicans* | *TmL27a* | 20.52 |
| TAK1 *C. albicans* | *TmL27a* | 19.76 | TAK1 *C. albicans* | *TmL27a* | 20.7 |

| Sample name | Gene name | Cq (∆R) | Sample name | Gene name | Cq (∆R) |
| --- | --- | --- | --- | --- | --- |
| EGFP PBS | *TmColeC* | 27.13 | EGFP PBS | *TmCec2* | 18.85 |
| EGFP PBS | *TmColeC* | 26.77 | EGFP PBS | *TmCec2* | 18.95 |
| EGFP PBS | *TmL27a* | 19.02 | EGFP PBS | *TmL27a* | 19.02 |
| EGFP PBS | *TmL27a* | 18.98 | EGFP PBS | *TmL27a* | 18.98 |
| TAK1 PBS | *TmColeC* | 27.38 | TAK1 PBS | *TmCec2* | 18.61 |
| TAK1 PBS | *TmColeC* | 27.27 | TAK1 PBS | *TmCec2* | 18.73 |
| TAK1 PBS | *TmL27a* | 18.8 | TAK1 PBS | *TmL27a* | 18.8 |
| TAK1 PBS | *TmL27a* | 18.65 | TAK1 PBS | *TmL27a* | 18.65 |
| EGFP *E. coli* | *TmColeC* | 17.86 | EGFP *E. coli* | *TmCec2* | 17.91 |
| EGFP *E. coli* | *TmColeC* | 17.84 | EGFP *E. coli* | *TmCec2* | 17.93 |
| EGFP *E. coli* | *TmL27a* | 18.09 | EGFP *E. coli* | *TmL27a* | 18.09 |
| EGFP *E. coli* | *TmL27a* | 18.02 | EGFP *E. coli* | *TmL27a* | 18.02 |
| TAK1 *E. coli* | *TmColeC* | 20.7 | TAK1 *E. coli* | *TmCec2* | 18.53 |
| TAK1 *E. coli* | *TmColeC* | 19.91 | TAK1 *E. coli* | *TmCec2* | 18.78 |
| TAK1 *E. coli* | *TmL27a* | 18.86 | TAK1 *E. coli* | *TmL27a* | 18.86 |
| TAK1 *E. coli* | *TmL27a* | 18.83 | TAK1 *E. coli* | *TmL27a* | 18.83 |
| EGFP *S. aureus* | *TmColeC* | 21.48 | EGFP *S. aureus* | *TmCec2* | 18.41 |
| EGFP *S. aureus* | *TmColeC* | 21.62 | EGFP *S. aureus* | *TmCec2* | 17.99 |
| EGFP *S. aureus* | *TmL27a* | 18.88 | EGFP *S. aureus* | *TmL27a* | 18.88 |
| EGFP *S. aureus* | *TmL27a* | 18.79 | EGFP *S. aureus* | *TmL27a* | 18.79 |
| TAK1 *S. aureus* | *TmColeC* | 21.53 | TAK1 *S. aureus* | *TmCec2* | 18.71 |
| TAK1 *S. aureus* | *TmColeC* | 21.61 | TAK1 *S. aureus* | *TmCec2* | 18.7 |
| TAK1 *S. aureus* | *TmL27a* | 18.81 | TAK1 *S. aureus* | *TmL27a* | 18.81 |
| TAK1 *S. aureus* | *TmL27a* | 18.9 | TAK1 *S. aureus* | *TmL27a* | 18.9 |
| EGFP *C. albicans* | *TmColeC* | 23.61 | EGFP *C. albicans* | *TmCec2* | 17.59 |
| EGFP *C. albicans* | *TmColeC* | 23.71 | EGFP *C. albicans* | *TmCec2* | 17.64 |
| EGFP *C. albicans* | *TmL27a* | 18.72 | EGFP *C. albicans* | *TmL27a* | 18.72 |
| EGFP *C. albicans* | *TmL27a* | 18.62 | EGFP *C. albicans* | *TmL27a* | 18.62 |
| TAK1 *C. albicans* | *TmColeC* | 24.2 | TAK1 *C. albicans* | *TmCec2* | 16.99 |
| TAK1 *C. albicans* | *TmColeC* | 24.5 | TAK1 *C. albicans* | *TmCec2* | 16.95 |
| TAK1 *C. albicans* | *TmL27a* | 18.57 | TAK1 *C. albicans* | *TmL27a* | 18.57 |
| TAK1 *C. albicans* | *TmL27a* | 18.08 | TAK1 *C. albicans* | *TmL27a* | 18.08 |

| Sample name | Gene name | Cq (∆R) | Sample name | Gene name | Cq (∆R) |
| --- | --- | --- | --- | --- | --- |
| EGFP PBS | *TmAtta1a* | 28.15 | EGFP PBS | *TmAtta1b* | 24.93 |
| EGFP PBS | *TmAtta1a* | 28.14 | EGFP PBS | *TmAtta1b* | 24.88 |
| EGFP PBS | *TmL27a* | 20.91 | EGFP PBS | *TmL27a* | 20.91 |
| EGFP PBS | *TmL27a* | 20.96 | EGFP PBS | *TmL27a* | 20.96 |
| TAK1 PBS | *TmAtta1a* | 28.66 | TAK1 PBS | *TmAtta1b* | 26.3 |
| TAK1 PBS | *TmAtta1a* | 28.09 | TAK1 PBS | *TmAtta1b* | 26.65 |
| TAK1 PBS | *TmL27a* | 20.97 | TAK1 PBS | *TmL27a* | 20.97 |
| TAK1 PBS | *TmL27a* | 20.91 | TAK1 PBS | *TmL27a* | 20.91 |
| EGFP *E. coli* | *TmAtta1a* | 19.13 | EGFP *E. coli* | *TmAtta1b* | 18.21 |
| EGFP *E. coli* | *TmAtta1a* | 19.11 | EGFP *E. coli* | *TmAtta1b* | 18.29 |
| EGFP *E. coli* | *TmL27a* | 20.28 | EGFP *E. coli* | *TmL27a* | 20.28 |
| EGFP *E. coli* | *TmL27a* | 20.32 | EGFP *E. coli* | *TmL27a* | 20.32 |
| TAK1 *E. coli* | *TmAtta1a* | 20.61 | TAK1 *E. coli* | *TmAtta1b* | 19.04 |
| TAK1 *E. coli* | *TmAtta1a* | 20.5 | TAK1 *E. coli* | *TmAtta1b* | 18.94 |
| TAK1 *E. coli* | *TmL27a* | 20.77 | TAK1 *E. coli* | *TmL27a* | 20.77 |
| TAK1 *E. coli* | *TmL27a* | 20.64 | TAK1 *E. coli* | *TmL27a* | 20.64 |
| EGFP *S. aureus* | *TmAtta1a* | 22.77 | EGFP *S. aureus* | *TmAtta1b* | 21.03 |
| EGFP *S. aureus* | *TmAtta1a* | 22.61 | EGFP *S. aureus* | *TmAtta1b* | 20.92 |
| EGFP *S. aureus* | *TmL27a* | 21.28 | EGFP *S. aureus* | *TmL27a* | 21.28 |
| EGFP *S. aureus* | *TmL27a* | 21.21 | EGFP *S. aureus* | *TmL27a* | 21.21 |
| TAK1 *S. aureus* | *TmAtta1a* | 22.78 | TAK1 *S. aureus* | *TmAtta1b* | 20.94 |
| TAK1 *S. aureus* | *TmAtta1a* | 22.86 | TAK1 *S. aureus* | *TmAtta1b* | 20.83 |
| TAK1 *S. aureus* | *TmL27a* | 21.01 | TAK1 *S. aureus* | *TmL27a* | 21.01 |
| TAK1 *S. aureus* | *TmL27a* | 20.88 | TAK1 *S. aureus* | *TmL27a* | 20.88 |
| EGFP *C. albicans* | *TmAtta1a* | 26 | EGFP *C. albicans* | *TmAtta1b* | 24.03 |
| EGFP *C. albicans* | *TmAtta1a* | 25.94 | EGFP *C. albicans* | *TmAtta1b* | 23.85 |
| EGFP *C. albicans* | *TmL27a* | 20.99 | EGFP *C. albicans* | *TmL27a* | 20.99 |
| EGFP *C. albicans* | *TmL27a* | 20.74 | EGFP *C. albicans* | *TmL27a* | 20.74 |
| TAK1 *C. albicans* | *TmAtta1a* | 26.28 | TAK1 *C. albicans* | *TmAtta1b* | 23.12 |
| TAK1 *C. albicans* | *TmAtta1a* | 26.25 | TAK1 *C. albicans* | *TmAtta1b* | 23.11 |
| TAK1 *C. albicans* | *TmL27a* | 20.68 | TAK1 *C. albicans* | *TmL27a* | 20.68 |
| TAK1 *C. albicans* | *TmL27a* | 20.44 | TAK1 *C. albicans* | *TmL27a* | 20.44 |

| Sample name | Gene name | Cq (∆R) | Sample name | Gene name | Cq (∆R) |
| --- | --- | --- | --- | --- | --- |
| EGFP PBS | *TmAtta2* | 23.54 | EGFP PBS | *TmTLP1* | 23.21 |
| EGFP PBS | *TmAtta2* | 23.3 | EGFP PBS | *TmTLP1* | 23.29 |
| EGFP PBS | *TmL27a* | 20.91 | EGFP PBS | *TmL27a* | 20.91 |
| EGFP PBS | *TmL27a* | 20.96 | EGFP PBS | *TmL27a* | 20.96 |
| TAK1 PBS | *TmAtta2* | 25.02 | TAK1 PBS | *TmTLP1* | 23.68 |
| TAK1 PBS | *TmAtta2* | 24.71 | TAK1 PBS | *TmTLP1* | 23.77 |
| TAK1 PBS | *TmL27a* | 20.97 | TAK1 PBS | *TmL27a* | 20.97 |
| TAK1 PBS | *TmL27a* | 20.91 | TAK1 PBS | *TmL27a* | 20.91 |
| EGFP *E. coli* | *TmAtta2* | 16.82 | EGFP *E. coli* | *TmTLP1* | 21.84 |
| EGFP *E. coli* | *TmAtta2* | 16.97 | EGFP *E. coli* | *TmTLP1* | 21.84 |
| EGFP *E. coli* | *TmL27a* | 20.28 | EGFP *E. coli* | *TmL27a* | 20.28 |
| EGFP *E. coli* | *TmL27a* | 20.32 | EGFP *E. coli* | *TmL27a* | 20.32 |
| TAK1 *E. coli* | *TmAtta2* | 18.62 | TAK1 *E. coli* | *TmTLP1* | 22.06 |
| TAK1 *E. coli* | *TmAtta2* | 18.39 | TAK1 *E. coli* | *TmTLP1* | 22 |
| TAK1 *E. coli* | *TmL27a* | 20.77 | TAK1 *E. coli* | *TmL27a* | 20.77 |
| TAK1 *E. coli* | *TmL27a* | 20.64 | TAK1 *E. coli* | *TmL27a* | 20.64 |
| EGFP *S. aureus* | *TmAtta2* | 19.45 | EGFP *S. aureus* | *TmTLP1* | 22.7 |
| EGFP *S. aureus* | *TmAtta2* | 19.6 | EGFP *S. aureus* | *TmTLP1* | 22.92 |
| EGFP *S. aureus* | *TmL27a* | 21.28 | EGFP *S. aureus* | *TmL27a* | 21.28 |
| EGFP *S. aureus* | *TmL27a* | 21.21 | EGFP *S. aureus* | *TmL27a* | 21.21 |
| TAK1 *S. aureus* | *TmAtta2* | 20.54 | TAK1 *S. aureus* | *TmTLP1* | 23.85 |
| TAK1 *S. aureus* | *TmAtta2* | 20.18 | TAK1 *S. aureus* | *TmTLP1* | 23.77 |
| TAK1 *S. aureus* | *TmL27a* | 21.01 | TAK1 *S. aureus* | *TmL27a* | 21.01 |
| TAK1 *S. aureus* | *TmL27a* | 20.88 | TAK1 *S. aureus* | *TmL27a* | 20.88 |
| EGFP *C. albicans* | *TmAtta2* | 22.09 | EGFP *C. albicans* | *TmTLP1* | 22.94 |
| EGFP *C. albicans* | *TmAtta2* | 22.08 | EGFP *C. albicans* | *TmTLP1* | 22.95 |
| EGFP *C. albicans* | *TmL27a* | 20.99 | EGFP *C. albicans* | *TmL27a* | 20.99 |
| EGFP *C. albicans* | *TmL27a* | 20.74 | EGFP *C. albicans* | *TmL27a* | 20.74 |
| TAK1 *C. albicans* | *TmAtta2* | 22.87 | TAK1 *C. albicans* | *TmTLP1* | 24.12 |
| TAK1 *C. albicans* | *TmAtta2* | 23.09 | TAK1 *C. albicans* | *TmTLP1* | 24.13 |
| TAK1 *C. albicans* | *TmL27a* | 20.68 | TAK1 *C. albicans* | *TmL27a* | 20.68 |
| TAK1 *C. albicans* | *TmL27a* | 20.44 | TAK1 *C. albicans* | *TmL27a* | 20.44 |

| Sample name | Gene name | Cq (∆R) |
| --- | --- | --- |
| EGFP PBS | *TmTLP2* | 31.43 |
| EGFP PBS | *TmTLP2* | 31.77 |
| EGFP PBS | *TmL27a* | 20.91 |
| EGFP PBS | *TmL27a* | 20.96 |
| TAK1 PBS | *TmTLP2* | 24.16 |
| TAK1 PBS | *TmTLP2* | 24.39 |
| TAK1 PBS | *TmL27a* | 20.97 |
| TAK1 PBS | *TmL27a* | 20.91 |
| EGFP *E. coli* | *TmTLP2* | 32.56 |
| EGFP *E. coli* | *TmTLP2* | 32.67 |
| EGFP *E. coli* | *TmL27a* | 20.28 |
| EGFP *E. coli* | *TmL27a* | 20.32 |
| TAK1 *E. coli* | *TmTLP2* | 32.25 |
| TAK1 *E. coli* | *TmTLP2* | 32.82 |
| TAK1 *E. coli* | *TmL27a* | 20.77 |
| TAK1 *E. coli* | *TmL27a* | 20.64 |
| EGFP *S. aureus* | *TmTLP2* | 32.57 |
| EGFP *S. aureus* | *TmTLP2* | 33.05 |
| EGFP *S. aureus* | *TmL27a* | 21.28 |
| EGFP *S. aureus* | *TmL27a* | 21.21 |
| TAK1 *S. aureus* | *TmTLP2* | 31.85 |
| TAK1 *S. aureus* | *TmTLP2* | 32.33 |
| TAK1 *S. aureus* | *TmL27a* | 21.01 |
| TAK1 *S. aureus* | *TmL27a* | 20.88 |
| EGFP *C. albicans* | *TmTLP2* | 30.96 |
| EGFP *C. albicans* | *TmTLP2* | 30.91 |
| EGFP *C. albicans* | *TmL27a* | 20.99 |
| EGFP *C. albicans* | *TmL27a* | 20.74 |
| TAK1 *C. albicans* | *TmTLP2* | 31.77 |
| TAK1 *C. albicans* | *TmTLP2* | 33.67 |
| TAK1 *C. albicans* | *TmL27a* | 20.68 |
| TAK1 *C. albicans* | *TmL27a* | 20.44 |

**Supplementary Table12. Raw data of qPCR in AMP expression patterns.**

Cq data of *TmTak1* and *TmL27a* in *T. molitor* at Malpighian tubules(MT).

| Sample name | Gene name | Cq (∆R) | Sample name | Gene name | Cq (∆R) |
| --- | --- | --- | --- | --- | --- |
| EGFP PBS | *TmTene1* | 23.83 | EGFP PBS | *TmTene2* | 32.63 |
| EGFP PBS | *TmTene1* | 23.12 | EGFP PBS | *TmTene2* | 32.21 |
| EGFP PBS | *TmL27a* | 22.27 | EGFP PBS | *TmL27a* | 22.27 |
| EGFP PBS | *TmL27a* | 22.14 | EGFP PBS | *TmL27a* | 22.14 |
| TAK1 PBS | *TmTene1* | 24.65 | TAK1 PBS | *TmTene2* | 30.82 |
| TAK1 PBS | *TmTene1* | 24.14 | TAK1 PBS | *TmTene2* | 30.6 |
| TAK1 PBS | *TmL27a* | 21.84 | TAK1 PBS | *TmL27a* | 21.84 |
| TAK1 PBS | *TmL27a* | 21.71 | TAK1 PBS | *TmL27a* | 21.71 |
| EGFP *E. coli* | *TmTene1* | 16.65 | EGFP *E. coli* | *TmTene2* | 21.41 |
| EGFP *E. coli* | *TmTene1* | 16.44 | EGFP *E. coli* | *TmTene2* | 21.4 |
| EGFP *E. coli* | *TmL27a* | 21.97 | EGFP *E. coli* | *TmL27a* | 21.97 |
| EGFP *E. coli* | *TmL27a* | 21.93 | EGFP *E. coli* | *TmL27a* | 21.93 |
| TAK1 *E. coli* | *TmTene1* | 15.82 | TAK1 *E. coli* | *TmTene2* | 22.41 |
| TAK1 *E. coli* | *TmTene1* | 15.18 | TAK1 *E. coli* | *TmTene2* | 22.18 |
| TAK1 *E. coli* | *TmL27a* | 20.32 | TAK1 *E. coli* | *TmL27a* | 20.32 |
| TAK1 *E. coli* | *TmL27a* | 19.89 | TAK1 *E. coli* | *TmL27a* | 19.89 |
| EGFP *S. aureus* | *TmTene1* | 15.06 | EGFP *S. aureus* | *TmTene2* | 21.87 |
| EGFP *S. aureus* | *TmTene1* | 14.81 | EGFP *S. aureus* | *TmTene2* | 21.87 |
| EGFP *S. aureus* | *TmL27a* | 19.51 | EGFP *S. aureus* | *TmL27a* | 19.51 |
| EGFP *S. aureus* | *TmL27a* | 19.39 | EGFP *S. aureus* | *TmL27a* | 19.39 |
| TAK1 *S. aureus* | *TmTene1* | 14.63 | TAK1 *S. aureus* | *TmTene2* | 22.31 |
| TAK1 *S. aureus* | *TmTene1* | 14.64 | TAK1 *S. aureus* | *TmTene2* | 22.31 |
| TAK1 *S. aureus* | *TmL27a* | 17.88 | TAK1 *S. aureus* | *TmL27a* | 17.88 |
| TAK1 *S. aureus* | *TmL27a* | 17.8 | TAK1 *S. aureus* | *TmL27a* | 17.8 |
| EGFP *C. albicans* | *TmTene1* | 18.32 | EGFP *C. albicans* | *TmTene2* | 29.91 |
| EGFP *C. albicans* | *TmTene1* | 18.24 | EGFP *C. albicans* | *TmTene2* | 29.31 |
| EGFP *C. albicans* | *TmL27a* | 20.72 | EGFP *C. albicans* | *TmL27a* | 20.72 |
| EGFP *C. albicans* | *TmL27a* | 20.58 | EGFP *C. albicans* | *TmL27a* | 20.58 |
| TAK1 *C. albicans* | *TmTene1* | 17.26 | TAK1 *C. albicans* | *TmTene2* | 27.71 |
| TAK1 *C. albicans* | *TmTene1* | 17.31 | TAK1 *C. albicans* | *TmTene2* | 27.04 |
| TAK1 *C. albicans* | *TmL27a* | 18.25 | TAK1 *C. albicans* | *TmL27a* | 18.25 |
| TAK1 *C. albicans* | *TmL27a* | 17.95 | TAK1 *C. albicans* | *TmL27a* | 17.95 |

| Sample name | Gene name | Cq (∆R) | Sample name | Gene name | Cq (∆R) |
| --- | --- | --- | --- | --- | --- |
| EGFP PBS | *TmTene3* | 22.23 | EGFP PBS | *TmTene4* | 28.67 |
| EGFP PBS | *TmTene3* | 22.26 | EGFP PBS | *TmTene4* | 29.07 |
| EGFP PBS | *TmL27a* | 23.06 | EGFP PBS | *TmL27a* | 23.06 |
| EGFP PBS | *TmL27a* | 22.99 | EGFP PBS | *TmL27a* | 22.99 |
| TAK1 PBS | *TmTene3* | 22.9 | TAK1 PBS | *TmTene4* | 27.12 |
| TAK1 PBS | *TmTene3* | 22.8 | TAK1 PBS | *TmTene4* | 27.12 |
| TAK1 PBS | *TmL27a* | 23.35 | TAK1 PBS | *TmL27a* | 23.35 |
| TAK1 PBS | *TmL27a* | 23.28 | TAK1 PBS | *TmL27a* | 23.28 |
| EGFP *E. coli* | *TmTene3* | 24.24 | EGFP *E. coli* | *TmTene4* | 18.4 |
| EGFP *E. coli* | *TmTene3* | 24.45 | EGFP *E. coli* | *TmTene4* | 18.35 |
| EGFP *E. coli* | *TmL27a* | 23.42 | EGFP *E. coli* | *TmL27a* | 23.42 |
| EGFP *E. coli* | *TmL27a* | 23.43 | EGFP *E. coli* | *TmL27a* | 23.43 |
| TAK1 *E. coli* | *TmTene3* | 20.92 | TAK1 *E. coli* | *TmTene4* | 18.33 |
| TAK1 *E. coli* | *TmTene3* | 20.88 | TAK1 *E. coli* | *TmTene4* | 18.31 |
| TAK1 *E. coli* | *TmL27a* | 21.08 | TAK1 *E. coli* | *TmL27a* | 21.08 |
| TAK1 *E. coli* | *TmL27a* | 21.2 | TAK1 *E. coli* | *TmL27a* | 21.2 |
| EGFP *S. aureus* | *TmTene3* | 20.16 | EGFP *S. aureus* | *TmTene4* | 17.9 |
| EGFP *S. aureus* | *TmTene3* | 20.49 | EGFP *S. aureus* | *TmTene4* | 17.73 |
| EGFP *S. aureus* | *TmL27a* | 20.95 | EGFP *S. aureus* | *TmL27a* | 20.95 |
| EGFP *S. aureus* | *TmL27a* | 20.52 | EGFP *S. aureus* | *TmL27a* | 20.52 |
| TAK1 *S. aureus* | *TmTene3* | 18.39 | TAK1 *S. aureus* | *TmTene4* | 17.98 |
| TAK1 *S. aureus* | *TmTene3* | 18.72 | TAK1 *S. aureus* | *TmTene4* | 17.94 |
| TAK1 *S. aureus* | *TmL27a* | 19.19 | TAK1 *S. aureus* | *TmL27a* | 19.19 |
| TAK1 *S. aureus* | *TmL27a* | 19.14 | TAK1 *S. aureus* | *TmL27a* | 19.14 |
| EGFP *C. albicans* | *TmTene3* | 21.47 | EGFP *C. albicans* | *TmTene4* | 22.09 |
| EGFP *C. albicans* | *TmTene3* | 21.62 | EGFP *C. albicans* | *TmTene4* | 22.05 |
| EGFP *C. albicans* | *TmL27a* | 22.13 | EGFP *C. albicans* | *TmL27a* | 22.13 |
| EGFP *C. albicans* | *TmL27a* | 21.92 | EGFP *C. albicans* | *TmL27a* | 21.92 |
| TAK1 *C. albicans* | *TmTene3* | 19.52 | TAK1 *C. albicans* | *TmTene4* | 23.17 |
| TAK1 *C. albicans* | *TmTene3* | 19.01 | TAK1 *C. albicans* | *TmTene4* | 22.96 |
| TAK1 *C. albicans* | *TmL27a* | 19.48 | TAK1 *C. albicans* | *TmL27a* | 19.48 |
| TAK1 *C. albicans* | *TmL27a* | 19.43 | TAK1 *C. albicans* | *TmL27a* | 19.43 |

| Sample name | Gene name | Cq (∆R) | Sample name | Gene name | Cq (∆R) |
| --- | --- | --- | --- | --- | --- |
| EGFP PBS | *TmDef* | 29.53 | EGFP PBS | *TmDef-like* | 24.82 |
| EGFP PBS | *TmDef* | 29.41 | EGFP PBS | *TmDef-like* | 24.74 |
| EGFP PBS | *TmL27a* | 22.27 | EGFP PBS | *TmL27a* | 21.82 |
| EGFP PBS | *TmL27a* | 22.14 | EGFP PBS | *TmL27a* | 21.85 |
| TAK1 PBS | *TmDef* | 26.94 | TAK1 PBS | *TmDef-like* | 26.66 |
| TAK1 PBS | *TmDef* | 26.51 | TAK1 PBS | *TmDef-like* | 26.7 |
| TAK1 PBS | *TmL27a* | 21.84 | TAK1 PBS | *TmL27a* | 22.69 |
| TAK1 PBS | *TmL27a* | 21.71 | TAK1 PBS | *TmL27a* | 22.13 |
| EGFP *E. coli* | *TmDef* | 18.31 | EGFP *E. coli* | *TmDef-like* | 18.07 |
| EGFP *E. coli* | *TmDef* | 18.25 | EGFP *E. coli* | *TmDef-like* | 17.98 |
| EGFP *E. coli* | *TmL27a* | 21.97 | EGFP *E. coli* | *TmL27a* | 22.26 |
| EGFP *E. coli* | *TmL27a* | 21.93 | EGFP *E. coli* | *TmL27a* | 22.71 |
| TAK1 *E. coli* | *TmDef* | 16.39 | TAK1 *E. coli* | *TmDef-like* | 16.88 |
| TAK1 *E. coli* | *TmDef* | 16.29 | TAK1 *E. coli* | *TmDef-like* | 16.8 |
| TAK1 *E. coli* | *TmL27a* | 20.32 | TAK1 *E. coli* | *TmL27a* | 19.96 |
| TAK1 *E. coli* | *TmL27a* | 19.89 | TAK1 *E. coli* | *TmL27a* | 19.94 |
| EGFP *S. aureus* | *TmDef* | 15.89 | EGFP *S. aureus* | *TmDef-like* | 16.75 |
| EGFP *S. aureus* | *TmDef* | 15.95 | EGFP *S. aureus* | *TmDef-like* | 16.81 |
| EGFP *S. aureus* | *TmL27a* | 19.51 | EGFP *S. aureus* | *TmL27a* | 19.75 |
| EGFP *S. aureus* | *TmL27a* | 19.39 | EGFP *S. aureus* | *TmL27a* | 19.71 |
| TAK1 *S. aureus* | *TmDef* | 15.22 | TAK1 *S. aureus* | *TmDef-like* | 15.6 |
| TAK1 *S. aureus* | *TmDef* | 14.99 | TAK1 *S. aureus* | *TmDef-like* | 15.3 |
| TAK1 *S. aureus* | *TmL27a* | 17.88 | TAK1 *S. aureus* | *TmL27a* | 18.52 |
| TAK1 *S. aureus* | *TmL27a* | 17.8 | TAK1 *S. aureus* | *TmL27a* | 18.5 |
| EGFP *C. albicans* | *TmDef* | 18.9 | EGFP *C. albicans* | *TmDef-like* | 20.74 |
| EGFP *C. albicans* | *TmDef* | 18.78 | EGFP *C. albicans* | *TmDef-like* | 20.69 |
| EGFP *C. albicans* | *TmL27a* | 20.72 | EGFP *C. albicans* | *TmL27a* | 20.98 |
| EGFP *C. albicans* | *TmL27a* | 20.58 | EGFP *C. albicans* | *TmL27a* | 20.94 |
| TAK1 *C. albicans* | *TmDef* | 18.08 | TAK1 *C. albicans* | *TmDef-like* | 18.75 |
| TAK1 *C. albicans* | *TmDef* | 17.99 | TAK1 *C. albicans* | *TmDef-like* | 18.73 |
| TAK1 *C. albicans* | *TmL27a* | 18.25 | TAK1 *C. albicans* | *TmL27a* | 18.62 |
| TAK1 *C. albicans* | *TmL27a* | 17.95 | TAK1 *C. albicans* | *TmL27a* | 18.72 |

| Sample name | Gene name | Cq (∆R) | Sample name | Gene name | Cq (∆R) |
| --- | --- | --- | --- | --- | --- |
| EGFP PBS | *TmColeA* | 27.45 | EGFP PBS | *TmColeB* | 34.91 |
| EGFP PBS | *TmColeA* | 27.41 | EGFP PBS | *TmColeB* | 34.05 |
| EGFP PBS | *TmL27a* | 21.85 | EGFP PBS | *TmL27a* | 21.82 |
| EGFP PBS | *TmL27a* | 21.82 | EGFP PBS | *TmL27a* | 21.85 |
| TAK1 PBS | *TmColeA* | 28.65 | TAK1 PBS | *TmColeB* | 34.23 |
| TAK1 PBS | *TmColeA* | 28.51 | TAK1 PBS | *TmColeB* | 35.58 |
| TAK1 PBS | *TmL27a* | 22.69 | TAK1 PBS | *TmL27a* | 22.69 |
| TAK1 PBS | *TmL27a* | 22.13 | TAK1 PBS | *TmL27a* | 22.13 |
| EGFP *E. coli* | *TmColeA* | 18.77 | EGFP *E. coli* | *TmColeB* | 25.46 |
| EGFP *E. coli* | *TmColeA* | 18.8 | EGFP *E. coli* | *TmColeB* | 24.7 |
| EGFP *E. coli* | *TmL27a* | 22.26 | EGFP *E. coli* | *TmL27a* | 22.26 |
| EGFP *E. coli* | *TmL27a* | 22.71 | EGFP *E. coli* | *TmL27a* | 22.71 |
| TAK1 *E. coli* | *TmColeA* | 18.11 | TAK1 *E. coli* | *TmColeB* | 24.74 |
| TAK1 *E. coli* | *TmColeA* | 18.57 | TAK1 *E. coli* | *TmColeB* | 24.75 |
| TAK1 *E. coli* | *TmL27a* | 19.96 | TAK1 *E. coli* | *TmL27a* | 19.94 |
| TAK1 *E. coli* | *TmL27a* | 19.94 | TAK1 *E. coli* | *TmL27a* | 19.96 |
| EGFP *S. aureus* | *TmColeA* | 17.53 | EGFP *S. aureus* | *TmColeB* | 24.37 |
| EGFP *S. aureus* | *TmColeA* | 17.26 | EGFP *S. aureus* | *TmColeB* | 24.46 |
| EGFP *S. aureus* | *TmL27a* | 19.75 | EGFP *S. aureus* | *TmL27a* | 19.75 |
| EGFP *S. aureus* | *TmL27a* | 19.71 | EGFP *S. aureus* | *TmL27a* | 19.71 |
| TAK1 *S. aureus* | *TmColeA* | 17.64 | TAK1 *S. aureus* | *TmColeB* | 24.48 |
| TAK1 *S. aureus* | *TmColeA* | 16.98 | TAK1 *S. aureus* | *TmColeB* | 23.88 |
| TAK1 *S. aureus* | *TmL27a* | 18.52 | TAK1 *S. aureus* | *TmL27a* | 18.52 |
| TAK1 *S. aureus* | *TmL27a* | 18.5 | TAK1 *S. aureus* | *TmL27a* | 18.5 |
| EGFP *C. albicans* | *TmColeA* | 22.62 | EGFP *C. albicans* | *TmColeB* | 29.41 |
| EGFP *C. albicans* | *TmColeA* | 22.78 | EGFP *C. albicans* | *TmColeB* | 29.47 |
| EGFP *C. albicans* | *TmL27a* | 20.98 | EGFP *C. albicans* | *TmL27a* | 20.98 |
| EGFP *C. albicans* | *TmL27a* | 20.94 | EGFP *C. albicans* | *TmL27a* | 20.94 |
| TAK1 *C. albicans* | *TmColeA* | 21.77 | TAK1 *C. albicans* | *TmColeB* | 29.16 |
| TAK1 *C. albicans* | *TmColeA* | 21.75 | TAK1 *C. albicans* | *TmColeB* | 29.38 |
| TAK1 *C. albicans* | *TmL27a* | 18.62 | TAK1 *C. albicans* | *TmL27a* | 18.62 |
| TAK1 *C. albicans* | *TmL27a* | 18.72 | TAK1 *C. albicans* | *TmL27a* | 18.72 |

| Sample name | Gene name | Cq (∆R) | Sample name | Gene name | Cq (∆R) |
| --- | --- | --- | --- | --- | --- |
| EGFP PBS | *TmColeC* | 29.88 | EGFP PBS | *TmCec2* | 24.56 |
| EGFP PBS | *TmColeC* | 30.13 | EGFP PBS | *TmCec2* | 24.49 |
| EGFP PBS | *TmL27a* | 21.82 | EGFP PBS | *TmL27a* | 21.82 |
| EGFP PBS | *TmL27a* | 21.85 | EGFP PBS | *TmL27a* | 21.85 |
| TAK1 PBS | *TmColeC* | 28.9 | TAK1 PBS | *TmCec2* | 26.11 |
| TAK1 PBS | *TmColeC* | 30.25 | TAK1 PBS | *TmCec2* | 24.57 |
| TAK1 PBS | *TmL27a* | 22.13 | TAK1 PBS | *TmL27a* | 22.69 |
| TAK1 PBS | *TmL27a* | 22.69 | TAK1 PBS | *TmL27a* | 22.13 |
| EGFP *E. coli* | *TmColeC* | 21.71 | EGFP *E. coli* | *TmCec2* | 22.45 |
| EGFP *E. coli* | *TmColeC* | 21.6 | EGFP *E. coli* | *TmCec2* | 21.92 |
| EGFP *E. coli* | *TmL27a* | 22.71 | EGFP *E. coli* | *TmL27a* | 22.71 |
| EGFP *E. coli* | *TmL27a* | 22.26 | EGFP *E. coli* | *TmL27a* | 22.26 |
| TAK1 *E. coli* | *TmColeC* | 19.9 | TAK1 *E. coli* | *TmCec2* | 20.57 |
| TAK1 *E. coli* | *TmColeC* | 19.84 | TAK1 *E. coli* | *TmCec2* | 20.74 |
| TAK1 *E. coli* | *TmL27a* | 19.96 | TAK1 *E. coli* | *TmL27a* | 19.96 |
| TAK1 *E. coli* | *TmL27a* | 19.94 | TAK1 *E. coli* | *TmL27a* | 19.94 |
| EGFP *S. aureus* | *TmColeC* | 19.8 | EGFP *S. aureus* | *TmCec2* | 19.69 |
| EGFP *S. aureus* | *TmColeC* | 20 | EGFP *S. aureus* | *TmCec2* | 19.7 |
| EGFP *S. aureus* | *TmL27a* | 19.75 | EGFP *S. aureus* | *TmL27a* | 19.75 |
| EGFP *S. aureus* | *TmL27a* | 19.71 | EGFP *S. aureus* | *TmL27a* | 19.71 |
| TAK1 *S. aureus* | *TmColeC* | 19.81 | TAK1 *S. aureus* | *TmCec2* | 20.41 |
| TAK1 *S. aureus* | *TmColeC* | 19.61 | TAK1 *S. aureus* | *TmCec2* | 20.31 |
| TAK1 *S. aureus* | *TmL27a* | 18.52 | TAK1 *S. aureus* | *TmL27a* | 18.52 |
| TAK1 *S. aureus* | *TmL27a* | 18.5 | TAK1 *S. aureus* | *TmL27a* | 18.5 |
| EGFP *C. albicans* | *TmColeC* | 25.74 | EGFP *C. albicans* | *TmCec2* | 21.4 |
| EGFP *C. albicans* | *TmColeC* | 25.77 | EGFP *C. albicans* | *TmCec2* | 20.93 |
| EGFP *C. albicans* | *TmL27a* | 20.98 | EGFP *C. albicans* | *TmL27a* | 20.98 |
| EGFP *C. albicans* | *TmL27a* | 20.94 | EGFP *C. albicans* | *TmL27a* | 20.94 |
| TAK1 *C. albicans* | *TmColeC* | 23.57 | TAK1 *C. albicans* | *TmCec2* | 18.74 |
| TAK1 *C. albicans* | *TmColeC* | 23.7 | TAK1 *C. albicans* | *TmCec2* | 18.85 |
| TAK1 *C. albicans* | *TmL27a* | 18.62 | TAK1 *C. albicans* | *TmL27a* | 18.62 |
| TAK1 *C. albicans* | *TmL27a* | 18.72 | TAK1 *C. albicans* | *TmL27a* | 18.72 |

| Sample name | Gene name | Cq (∆R) | Sample name | Gene name | Cq (∆R) |
| --- | --- | --- | --- | --- | --- |
| EGFP PBS | *TmAtta1a* | 29.51 | EGFP PBS | *TmAtta1b* | 28.89 |
| EGFP PBS | *TmAtta1a* | 28.68 | EGFP PBS | *TmAtta1b* | 28.88 |
| EGFP PBS | *TmL27a* | 22.79 | EGFP PBS | *TmL27a* | 22.79 |
| EGFP PBS | *TmL27a* | 22.48 | EGFP PBS | *TmL27a* | 22.48 |
| TAK1 PBS | *TmAtta1a* | 28.2 | TAK1 PBS | *TmAtta1b* | 27.47 |
| TAK1 PBS | *TmAtta1a* | 28.16 | TAK1 PBS | *TmAtta1b* | 27.75 |
| TAK1 PBS | *TmL27a* | 22.9 | TAK1 PBS | *TmL27a* | 23.27 |
| TAK1 PBS | *TmL27a* | 23.27 | TAK1 PBS | *TmL27a* | 22.9 |
| EGFP *E. coli* | *TmAtta1a* | 20.55 | EGFP *E. coli* | *TmAtta1b* | 19.09 |
| EGFP *E. coli* | *TmAtta1a* | 20.28 | EGFP *E. coli* | *TmAtta1b* | 19.12 |
| EGFP *E. coli* | *TmL27a* | 23.2 | EGFP *E. coli* | *TmL27a* | 23.2 |
| EGFP *E. coli* | *TmL27a* | 23.09 | EGFP *E. coli* | *TmL27a* | 23.09 |
| TAK1 *E. coli* | *TmAtta1a* | 19.9 | TAK1 *E. coli* | *TmAtta1b* | 18.41 |
| TAK1 *E. coli* | *TmAtta1a* | 19.66 | TAK1 *E. coli* | *TmAtta1b* | 18.36 |
| TAK1 *E. coli* | *TmL27a* | 20.66 | TAK1 *E. coli* | *TmL27a* | 20.66 |
| TAK1 *E. coli* | *TmL27a* | 20.51 | TAK1 *E. coli* | *TmL27a* | 20.51 |
| EGFP *S. aureus* | *TmAtta1a* | 19.45 | EGFP *S. aureus* | *TmAtta1b* | 18.01 |
| EGFP *S. aureus* | *TmAtta1a* | 19.31 | EGFP *S. aureus* | *TmAtta1b* | 18.04 |
| EGFP *S. aureus* | *TmL27a* | 20.45 | EGFP *S. aureus* | *TmL27a* | 20.45 |
| EGFP *S. aureus* | *TmL27a* | 20.15 | EGFP *S. aureus* | *TmL27a* | 20.15 |
| TAK1 *S. aureus* | *TmAtta1a* | 18.71 | TAK1 *S. aureus* | *TmAtta1b* | 18.05 |
| TAK1 *S. aureus* | *TmAtta1a* | 18.64 | TAK1 *S. aureus* | *TmAtta1b* | 18.04 |
| TAK1 *S. aureus* | *TmL27a* | 19.03 | TAK1 *S. aureus* | *TmL27a* | 19.03 |
| TAK1 *S. aureus* | *TmL27a* | 18.52 | TAK1 *S. aureus* | *TmL27a* | 18.52 |
| EGFP *C. albicans* | *TmAtta1a* | 24.16 | EGFP *C. albicans* | *TmAtta1b* | 21.95 |
| EGFP *C. albicans* | *TmAtta1a* | 24.14 | EGFP *C. albicans* | *TmAtta1b* | 21.9 |
| EGFP *C. albicans* | *TmL27a* | 21.64 | EGFP *C. albicans* | *TmL27a* | 21.64 |
| EGFP *C. albicans* | *TmL27a* | 21.39 | EGFP *C. albicans* | *TmL27a* | 21.39 |
| TAK1 *C. albicans* | *TmAtta1a* | 23.16 | TAK1 *C. albicans* | *TmAtta1b* | 21.3 |
| TAK1 *C. albicans* | *TmAtta1a* | 23.22 | TAK1 *C. albicans* | *TmAtta1b* | 21.39 |
| TAK1 *C. albicans* | *TmL27a* | 19.32 | TAK1 *C. albicans* | *TmL27a* | 19.32 |
| TAK1 *C. albicans* | *TmL27a* | 19 | TAK1 *C. albicans* | *TmL27a* | 19 |

| Sample name | Gene name | Cq (∆R) | Sample name | Gene name | Cq (∆R) |
| --- | --- | --- | --- | --- | --- |
| EGFP PBS | *TmAtta2* | 29.21 | EGFP PBS | *TmTLP1* | 31.46 |
| EGFP PBS | *TmAtta2* | 28.84 | EGFP PBS | *TmTLP1* | 31.68 |
| EGFP PBS | *TmL27a* | 22.79 | EGFP PBS | *TmL27a* | 22.79 |
| EGFP PBS | *TmL27a* | 22.48 | EGFP PBS | *TmL27a* | 22.48 |
| TAK1 PBS | *TmAtta2* | 28.76 | TAK1 PBS | *TmTLP1* | 29.81 |
| TAK1 PBS | *TmAtta2* | 29.1 | TAK1 PBS | *TmTLP1* | 30.1 |
| TAK1 PBS | *TmL27a* | 23.27 | TAK1 PBS | *TmL27a* | 23.27 |
| TAK1 PBS | *TmL27a* | 22.9 | TAK1 PBS | *TmL27a* | 22.9 |
| EGFP *E. coli* | *TmAtta2* | 18.13 | EGFP *E. coli* | *TmTLP1* | 27.73 |
| EGFP *E. coli* | *TmAtta2* | 18.09 | EGFP *E. coli* | *TmTLP1* | 27.97 |
| EGFP *E. coli* | *TmL27a* | 23.2 | EGFP *E. coli* | *TmL27a* | 23.2 |
| EGFP *E. coli* | *TmL27a* | 23.09 | EGFP *E. coli* | *TmL27a* | 23.09 |
| TAK1 *E. coli* | *TmAtta2* | 17.49 | TAK1 *E. coli* | *TmTLP1* | 24.48 |
| TAK1 *E. coli* | *TmAtta2* | 17.41 | TAK1 *E. coli* | *TmTLP1* | 24.44 |
| TAK1 *E. coli* | *TmL27a* | 20.66 | TAK1 *E. coli* | *TmL27a* | 20.66 |
| TAK1 *E. coli* | *TmL27a* | 20.51 | TAK1 *E. coli* | *TmL27a* | 20.51 |
| EGFP *S. aureus* | *TmAtta2* | 17.38 | EGFP *S. aureus* | *TmTLP1* | 27.37 |
| EGFP *S. aureus* | *TmAtta2* | 17.52 | EGFP *S. aureus* | *TmTLP1* | 27.66 |
| EGFP *S. aureus* | *TmL27a* | 20.45 | EGFP *S. aureus* | *TmL27a* | 20.45 |
| EGFP *S. aureus* | *TmL27a* | 20.15 | EGFP *S. aureus* | *TmL27a* | 20.15 |
| TAK1 *S. aureus* | *TmAtta2* | 17.84 | TAK1 *S. aureus* | *TmTLP1* | 27.61 |
| TAK1 *S. aureus* | *TmAtta2* | 17.45 | TAK1 *S. aureus* | *TmTLP1* | 28.01 |
| TAK1 *S. aureus* | *TmL27a* | 19.03 | TAK1 *S. aureus* | *TmL27a* | 19.03 |
| TAK1 *S. aureus* | *TmL27a* | 18.52 | TAK1 *S. aureus* | *TmL27a* | 18.52 |
| EGFP *C. albicans* | *TmAtta2* | 22.18 | EGFP *C. albicans* | *TmTLP1* | 30.05 |
| EGFP *C. albicans* | *TmAtta2* | 22.28 | EGFP *C. albicans* | *TmTLP1* | 30.08 |
| EGFP *C. albicans* | *TmL27a* | 21.64 | EGFP *C. albicans* | *TmL27a* | 21.64 |
| EGFP *C. albicans* | *TmL27a* | 21.39 | EGFP *C. albicans* | *TmL27a* | 21.39 |
| TAK1 *C. albicans* | *TmAtta2* | 22.17 | TAK1 *C. albicans* | *TmTLP1* | 27.7 |
| TAK1 *C. albicans* | *TmAtta2* | 22.12 | TAK1 *C. albicans* | *TmTLP1* | 27.92 |
| TAK1 *C. albicans* | *TmL27a* | 19.32 | TAK1 *C. albicans* | *TmL27a* | 19.32 |
| TAK1 *C. albicans* | *TmL27a* | 19 | TAK1 *C. albicans* | *TmL27a* | 19 |

| Sample name | Gene name | Cq (∆R) |
| --- | --- | --- |
| EGFP PBS | *TmTLP2* | 28.06 |
| EGFP PBS | *TmTLP2* | 28.37 |
| EGFP PBS | *TmL27a* | 22.79 |
| EGFP PBS | *TmL27a* | 22.48 |
| TAK1 PBS | *TmTLP2* | 29.13 |
| TAK1 PBS | *TmTLP2* | 29.6 |
| TAK1 PBS | *TmL27a* | 23.27 |
| TAK1 PBS | *TmL27a* | 22.9 |
| EGFP *E. coli* | *TmTLP2* | 31.68 |
| EGFP *E. coli* | *TmTLP2* | 30.28 |
| EGFP *E. coli* | *TmL27a* | 23.2 |
| EGFP *E. coli* | *TmL27a* | 23.09 |
| TAK1 *E. coli* | *TmTLP2* | 30.9 |
| TAK1 *E. coli* | *TmTLP2* | 30.33 |
| TAK1 *E. coli* | *TmL27a* | 20.66 |
| TAK1 *E. coli* | *TmL27a* | 20.51 |
| EGFP *S. aureus* | *TmTLP2* | 29.85 |
| EGFP *S. aureus* | *TmTLP2* | 30.01 |
| EGFP *S. aureus* | *TmL27a* | 20.45 |
| EGFP *S. aureus* | *TmL27a* | 20.15 |
| TAK1 *S. aureus* | *TmTLP2* | 28.07 |
| TAK1 *S. aureus* | *TmTLP2* | 28.02 |
| TAK1 *S. aureus* | *TmL27a* | 19.03 |
| TAK1 *S. aureus* | *TmL27a* | 18.52 |
| EGFP *C. albicans* | *TmTLP2* | 29.59 |
| EGFP *C. albicans* | *TmTLP2* | 29.24 |
| EGFP *C. albicans* | *TmL27a* | 21.64 |
| EGFP *C. albicans* | *TmL27a* | 21.39 |
| TAK1 *C. albicans* | *TmTLP2* | 26.12 |
| TAK1 *C. albicans* | *TmTLP2* | 26.03 |
| TAK1 *C. albicans* | *TmL27a* | 19.32 |
| TAK1 *C. albicans* | *TmL27a* | 19 |

**Supplementary Table13. Raw data of qPCR in AMP expression patterns.**

Cq data of *TmTak1* and *TmL27a* in *T. molitor* at Hemocytes(HC)

| Sample name | Gene name | Cq (∆R) | Sample name | Gene name | Cq (∆R) |
| --- | --- | --- | --- | --- | --- |
| EGFP PBS | *TmTene1* | 27.12 | EGFP PBS | *TmTene2* | 30.09 |
| EGFP PBS | *TmTene1* | 27.02 | EGFP PBS | *TmTene2* | 30.55 |
| EGFP PBS | *TmL27a* | 23.51 | EGFP PBS | *TmL27a* | 23.51 |
| EGFP PBS | *TmL27a* | 23.42 | EGFP PBS | *TmL27a* | 23.42 |
| TAK1 PBS | *TmTene1* | 26.23 | TAK1 PBS | *TmTene2* | 30.8 |
| TAK1 PBS | *TmTene1* | 26.19 | TAK1 PBS | *TmTene2* | 29.68 |
| TAK1 PBS | *TmL27a* | 23.27 | TAK1 PBS | *TmL27a* | 23.27 |
| TAK1 PBS | *TmL27a* | 23.03 | TAK1 PBS | *TmL27a* | 23.03 |
| EGFP *E. coli* | *TmTene1* | 20.91 | EGFP *E. coli* | *TmTene2* | 25.47 |
| EGFP *E. coli* | *TmTene1* | 20.42 | EGFP *E. coli* | *TmTene2* | 25.53 |
| EGFP *E. coli* | *TmL27a* | 23.66 | EGFP *E. coli* | *TmL27a* | 23.66 |
| EGFP *E. coli* | *TmL27a* | 23.55 | EGFP *E. coli* | *TmL27a* | 23.55 |
| TAK1 *E. coli* | *TmTene1* | 17.6 | TAK1 *E. coli* | *TmTene2* | 24.97 |
| TAK1 *E. coli* | *TmTene1* | 17.43 | TAK1 *E. coli* | *TmTene2* | 25.03 |
| TAK1 *E. coli* | *TmL27a* | 19.2 | TAK1 *E. coli* | *TmL27a* | 19.2 |
| TAK1 *E. coli* | *TmL27a* | 18.82 | TAK1 *E. coli* | *TmL27a* | 18.82 |
| EGFP *S. aureus* | *TmTene1* | 21.1 | EGFP *S. aureus* | *TmTene2* | 24.19 |
| EGFP *S. aureus* | *TmTene1* | 21.14 | EGFP *S. aureus* | *TmTene2* | 24.45 |
| EGFP *S. aureus* | *TmL27a* | 21.43 | EGFP *S. aureus* | *TmL27a* | 21.43 |
| EGFP *S. aureus* | *TmL27a* | 21.33 | EGFP *S. aureus* | *TmL27a* | 21.33 |
| TAK1 *S. aureus* | *TmTene1* | 21.95 | TAK1 *S. aureus* | *TmTene2* | 28.4 |
| TAK1 *S. aureus* | *TmTene1* | 22.03 | TAK1 *S. aureus* | *TmTene2* | 29.1 |
| TAK1 *S. aureus* | *TmL27a* | 21.39 | TAK1 *S. aureus* | *TmL27a* | 21.39 |
| TAK1 *S. aureus* | *TmL27a* | 21.28 | TAK1 *S. aureus* | *TmL27a* | 21.28 |
| EGFP *C. albicans* | *TmTene1* | 23.97 | EGFP *C. albicans* | *TmTene2* | 30.71 |
| EGFP *C. albicans* | *TmTene1* | 24.06 | EGFP *C. albicans* | *TmTene2* | 30.72 |
| EGFP *C. albicans* | *TmL27a* | 22.56 | EGFP *C. albicans* | *TmL27a* | 22.56 |
| EGFP *C. albicans* | *TmL27a* | 22.38 | EGFP *C. albicans* | *TmL27a* | 22.38 |
| TAK1 *C. albicans* | *TmTene1* | 23.91 | TAK1 *C. albicans* | *TmTene2* | 33.14 |
| TAK1 *C. albicans* | *TmTene1* | 24.08 | TAK1 *C. albicans* | *TmTene2* | 36.33 |
| TAK1 *C. albicans* | *TmL27a* | 20.12 | TAK1 *C. albicans* | *TmL27a* | 20.12 |
| TAK1 *C. albicans* | *TmL27a* | 20.1 | TAK1 *C. albicans* | *TmL27a* | 20.1 |

| Sample name | Gene name | Cq (∆R) | Sample name | Gene name | Cq (∆R) |
| --- | --- | --- | --- | --- | --- |
| EGFP PBS | *TmTene3* | 25.66 | EGFP PBS | *TmTene4* | 27.88 |
| EGFP PBS | *TmTene3* | 25.68 | EGFP PBS | *TmTene4* | 28.33 |
| EGFP PBS | *TmL27a* | 24.51 | EGFP PBS | *TmL27a* | 24.51 |
| EGFP PBS | *TmL27a* | 24.28 | EGFP PBS | *TmL27a* | 24.28 |
| TAK1 PBS | *TmTene3* | 25.83 | TAK1 PBS | *TmTene4* | 26.23 |
| TAK1 PBS | *TmTene3* | 25.91 | TAK1 PBS | *TmTene4* | 26.15 |
| TAK1 PBS | *TmL27a* | 23.83 | TAK1 PBS | *TmL27a* | 23.83 |
| TAK1 PBS | *TmL27a* | 23.71 | TAK1 PBS | *TmL27a* | 23.71 |
| EGFP *E. coli* | *TmTene3* | 28.38 | EGFP *E. coli* | *TmTene4* | 22.24 |
| EGFP *E. coli* | *TmTene3* | 28.16 | EGFP *E. coli* | *TmTene4* | 22.5 |
| EGFP *E. coli* | *TmL27a* | 24.4 | EGFP *E. coli* | *TmL27a* | 24.4 |
| EGFP *E. coli* | *TmL27a* | 24.31 | EGFP *E. coli* | *TmL27a* | 24.31 |
| TAK1 *E. coli* | *TmTene3* | 23.34 | TAK1 *E. coli* | *TmTene4* | 19.51 |
| TAK1 *E. coli* | *TmTene3* | 23.44 | TAK1 *E. coli* | *TmTene4* | 18.99 |
| TAK1 *E. coli* | *TmL27a* | 19.75 | TAK1 *E. coli* | *TmL27a* | 19.75 |
| TAK1 *E. coli* | *TmL27a* | 19.66 | TAK1 *E. coli* | *TmL27a* | 19.66 |
| EGFP *S. aureus* | *TmTene3* | 25.71 | EGFP *S. aureus* | *TmTene4* | 23.47 |
| EGFP *S. aureus* | *TmTene3* | 26.44 | EGFP *S. aureus* | *TmTene4* | 24.22 |
| EGFP *S. aureus* | *TmL27a* | 21.89 | EGFP *S. aureus* | *TmL27a* | 21.89 |
| EGFP *S. aureus* | *TmL27a* | 21.88 | EGFP *S. aureus* | *TmL27a* | 21.88 |
| TAK1 *S. aureus* | *TmTene3* | 26.59 | TAK1 *S. aureus* | *TmTene4* | 23.95 |
| TAK1 *S. aureus* | *TmTene3* | 25.99 | TAK1 *S. aureus* | *TmTene4* | 24.34 |
| TAK1 *S. aureus* | *TmL27a* | 21.8 | TAK1 *S. aureus* | *TmL27a* | 21.8 |
| TAK1 *S. aureus* | *TmL27a* | 21.79 | TAK1 *S. aureus* | *TmL27a* | 21.79 |
| EGFP *C. albicans* | *TmTene3* | 26.55 | EGFP *C. albicans* | *TmTene4* | 28.97 |
| EGFP *C. albicans* | *TmTene3* | 26.44 | EGFP *C. albicans* | *TmTene4* | 29.28 |
| EGFP *C. albicans* | *TmL27a* | 23.81 | EGFP *C. albicans* | *TmL27a* | 23.81 |
| EGFP *C. albicans* | *TmL27a* | 23.34 | EGFP *C. albicans* | *TmL27a* | 23.34 |
| TAK1 *C. albicans* | *TmTene3* | 24.68 | TAK1 *C. albicans* | *TmTene4* | 27.59 |
| TAK1 *C. albicans* | *TmTene3* | 24.38 | TAK1 *C. albicans* | *TmTene4* | 27.68 |
| TAK1 *C. albicans* | *TmL27a* | 20.89 | TAK1 *C. albicans* | *TmL27a* | 20.89 |
| TAK1 *C. albicans* | *TmL27a* | 20.88 | TAK1 *C. albicans* | *TmL27a* | 20.88 |

| Sample name | Gene name | Cq (∆R) | Sample name | Gene name | Cq (∆R) |
| --- | --- | --- | --- | --- | --- |
| EGFP PBS | *TmDef* | 28.98 | EGFP PBS | *TmDef-like* | 28.72 |
| EGFP PBS | *TmDef* | 29.21 | EGFP PBS | *TmDef-like* | 28.66 |
| EGFP PBS | *TmL27a* | 23.51 | EGFP PBS | *TmL27a* | 24.67 |
| EGFP PBS | *TmL27a* | 23.42 | EGFP PBS | *TmL27a* | 24.68 |
| TAK1 PBS | *TmDef* | 27.7 | TAK1 PBS | *TmDef-like* | 29.2 |
| TAK1 PBS | *TmDef* | 27.8 | TAK1 PBS | *TmDef-like* | 28.3 |
| TAK1 PBS | *TmL27a* | 23.27 | TAK1 PBS | *TmL27a* | 24.03 |
| TAK1 PBS | *TmL27a* | 23.03 | TAK1 PBS | *TmL27a* | 24.06 |
| EGFP *E. coli* | *TmDef* | 22.13 | EGFP *E. coli* | *TmDef-like* | 23.61 |
| EGFP *E. coli* | *TmDef* | 22.17 | EGFP *E. coli* | *TmDef-like* | 22.93 |
| EGFP *E. coli* | *TmL27a* | 23.66 | EGFP *E. coli* | *TmL27a* | 24.76 |
| EGFP *E. coli* | *TmL27a* | 23.55 | EGFP *E. coli* | *TmL27a* | 24.65 |
| TAK1 *E. coli* | *TmDef* | 19.03 | TAK1 *E. coli* | *TmDef-like* | 20.22 |
| TAK1 *E. coli* | *TmDef* | 19.11 | TAK1 *E. coli* | *TmDef-like* | 19.93 |
| TAK1 *E. coli* | *TmL27a* | 19.2 | TAK1 *E. coli* | *TmL27a* | 20 |
| TAK1 *E. coli* | *TmL27a* | 18.82 | TAK1 *E. coli* | *TmL27a* | 19.82 |
| EGFP *S. aureus* | *TmDef* | 22.88 | EGFP *S. aureus* | *TmDef-like* | 23.57 |
| EGFP *S. aureus* | *TmDef* | 22.84 | EGFP *S. aureus* | *TmDef-like* | 23.26 |
| EGFP *S. aureus* | *TmL27a* | 21.43 | EGFP *S. aureus* | *TmL27a* | 22.55 |
| EGFP *S. aureus* | *TmL27a* | 21.33 | EGFP *S. aureus* | *TmL27a* | 22.1 |
| TAK1 *S. aureus* | *TmDef* | 23.76 | TAK1 *S. aureus* | *TmDef-like* | 23.9 |
| TAK1 *S. aureus* | *TmDef* | 23.61 | TAK1 *S. aureus* | *TmDef-like* | 23.9 |
| TAK1 *S. aureus* | *TmL27a* | 21.39 | TAK1 *S. aureus* | *TmL27a* | 22.32 |
| TAK1 *S. aureus* | *TmL27a* | 21.28 | TAK1 *S. aureus* | *TmL27a* | 22.04 |
| EGFP *C. albicans* | *TmDef* | 24.88 | EGFP *C. albicans* | *TmDef-like* | 27.04 |
| EGFP *C. albicans* | *TmDef* | 24.77 | EGFP *C. albicans* | *TmDef-like* | 27.2 |
| EGFP *C. albicans* | *TmL27a* | 22.56 | EGFP *C. albicans* | *TmL27a* | 23.8 |
| EGFP *C. albicans* | *TmL27a* | 22.38 | EGFP *C. albicans* | *TmL27a* | 23.68 |
| TAK1 *C. albicans* | *TmDef* | 24.76 | TAK1 *C. albicans* | *TmDef-like* | 25.59 |
| TAK1 *C. albicans* | *TmDef* | 24.29 | TAK1 *C. albicans* | *TmDef-like* | 25.88 |
| TAK1 *C. albicans* | *TmL27a* | 20.12 | TAK1 *C. albicans* | *TmL27a* | 21.01 |
| TAK1 *C. albicans* | *TmL27a* | 20.1 | TAK1 *C. albicans* | *TmL27a* | 20.94 |

| Sample name | Gene name | Cq (∆R) | Sample name | Gene name | Cq (∆R) |
| --- | --- | --- | --- | --- | --- |
| EGFP PBS | *TmColeA* | 29.68 | EGFP PBS | *TmColeB* | 34.93 |
| EGFP PBS | *TmColeA* | 29.72 | EGFP PBS | *TmColeB* | 35.81 |
| EGFP PBS | *TmL27a* | 24.67 | EGFP PBS | *TmL27a* | 26.34 |
| EGFP PBS | *TmL27a* | 24.68 | EGFP PBS | *TmL27a* | 26.46 |
| TAK1 PBS | *TmColeA* | 28.7 | TAK1 PBS | *TmColeB* | 34.23 |
| TAK1 PBS | *TmColeA* | 28.87 | TAK1 PBS | *TmColeB* | 34.82 |
| TAK1 PBS | *TmL27a* | 24.03 | TAK1 PBS | *TmL27a* | 26.09 |
| TAK1 PBS | *TmL27a* | 24.06 | TAK1 PBS | *TmL27a* | 26.28 |
| EGFP *E. coli* | *TmColeA* | 24.82 | EGFP *E. coli* | *TmColeB* | 31.34 |
| EGFP *E. coli* | *TmColeA* | 24.89 | EGFP *E. coli* | *TmColeB* | 31.35 |
| EGFP *E. coli* | *TmL27a* | 24.76 | EGFP *E. coli* | *TmL27a* | 26.48 |
| EGFP *E. coli* | *TmL27a* | 24.65 | EGFP *E. coli* | *TmL27a* | 26.54 |
| TAK1 *E. coli* | *TmColeA* | 21.92 | TAK1 *E. coli* | *TmColeB* | 27.07 |
| TAK1 *E. coli* | *TmColeA* | 21.86 | TAK1 *E. coli* | *TmColeB* | 27.27 |
| TAK1 *E. coli* | *TmL27a* | 20 | TAK1 *E. coli* | *TmL27a* | 20.93 |
| TAK1 *E. coli* | *TmL27a* | 19.82 | TAK1 *E. coli* | *TmL27a* | 21 |
| EGFP *S. aureus* | *TmColeA* | 24.75 | EGFP *S. aureus* | *TmColeB* | 30.05 |
| EGFP *S. aureus* | *TmColeA* | 24.73 | EGFP *S. aureus* | *TmColeB* | 30.02 |
| EGFP *S. aureus* | *TmL27a* | 22.55 | EGFP *S. aureus* | *TmL27a* | 24 |
| EGFP *S. aureus* | *TmL27a* | 22.1 | EGFP *S. aureus* | *TmL27a* | 24 |
| TAK1 *S. aureus* | *TmColeA* | 25.75 | TAK1 *S. aureus* | *TmColeB* | 31.78 |
| TAK1 *S. aureus* | *TmColeA* | 25.57 | TAK1 *S. aureus* | *TmColeB* | 32.33 |
| TAK1 *S. aureus* | *TmL27a* | 22.32 | TAK1 *S. aureus* | *TmL27a* | 24.07 |
| TAK1 *S. aureus* | *TmL27a* | 22.04 | TAK1 *S. aureus* | *TmL27a* | 24.16 |
| EGFP *C. albicans* | *TmColeA* | 30.16 | EGFP *C. albicans* | *TmColeB* | 34.08 |
| EGFP *C. albicans* | *TmColeA* | 29.62 | EGFP *C. albicans* | *TmColeB* | 34.21 |
| EGFP *C. albicans* | *TmL27a* | 23.8 | EGFP *C. albicans* | *TmL27a* | 26.03 |
| EGFP *C. albicans* | *TmL27a* | 23.68 | EGFP *C. albicans* | *TmL27a* | 26.03 |
| TAK1 *C. albicans* | *TmColeA* | 28.54 | TAK1 *C. albicans* | *TmColeB* | 33.24 |
| TAK1 *C. albicans* | *TmColeA* | 27.72 | TAK1 *C. albicans* | *TmColeB* | 36.03 |
| TAK1 *C. albicans* | *TmL27a* | 21.01 | TAK1 *C. albicans* | *TmL27a* | 22.32 |
| TAK1 *C. albicans* | *TmL27a* | 20.94 | TAK1 *C. albicans* | *TmL27a* | 22.3 |

| Sample name | Gene name | Cq (∆R) | Sample name | Gene name | Cq (∆R) |
| --- | --- | --- | --- | --- | --- |
| EGFP PBS | *TmColeC* | 30.2 | EGFP PBS | *TmCec2* | 20.67 |
| EGFP PBS | *TmColeC* | 30.36 | EGFP PBS | *TmCec2* | 20.66 |
| EGFP PBS | *TmL27a* | 24.67 | EGFP PBS | *TmL27a* | 24.67 |
| EGFP PBS | *TmL27a* | 24.68 | EGFP PBS | *TmL27a* | 24.68 |
| TAK1 PBS | *TmColeC* | 29.21 | TAK1 PBS | *TmCec2* | 19.87 |
| TAK1 PBS | *TmColeC* | 30.32 | TAK1 PBS | *TmCec2* | 20.71 |
| TAK1 PBS | *TmL27a* | 24.03 | TAK1 PBS | *TmL27a* | 24.03 |
| TAK1 PBS | *TmL27a* | 24.06 | TAK1 PBS | *TmL27a* | 24.06 |
| EGFP *E. coli* | *TmColeC* | 26.65 | EGFP *E. coli* | *TmCec2* | 22.37 |
| EGFP *E. coli* | *TmColeC* | 27.06 | EGFP *E. coli* | *TmCec2* | 22.9 |
| EGFP *E. coli* | *TmL27a* | 24.76 | EGFP *E. coli* | *TmL27a* | 24.76 |
| EGFP *E. coli* | *TmL27a* | 24.65 | EGFP *E. coli* | *TmL27a* | 24.65 |
| TAK1 *E. coli* | *TmColeC* | 23.55 | TAK1 *E. coli* | *TmCec2* | 16.17 |
| TAK1 *E. coli* | *TmColeC* | 23.15 | TAK1 *E. coli* | *TmCec2* | 16.01 |
| TAK1 *E. coli* | *TmL27a* | 20 | TAK1 *E. coli* | *TmL27a* | 20 |
| TAK1 *E. coli* | *TmL27a* | 19.82 | TAK1 *E. coli* | *TmL27a* | 19.82 |
| EGFP *S. aureus* | *TmColeC* | 27.97 | EGFP *S. aureus* | *TmCec2* | 18.58 |
| EGFP *S. aureus* | *TmColeC* | 27.81 | EGFP *S. aureus* | *TmCec2* | 18.54 |
| EGFP *S. aureus* | *TmL27a* | 22.55 | EGFP *S. aureus* | *TmL27a* | 22.55 |
| EGFP *S. aureus* | *TmL27a* | 22.1 | EGFP *S. aureus* | *TmL27a* | 22.1 |
| TAK1 *S. aureus* | *TmColeC* | 27.53 | TAK1 *S. aureus* | *TmCec2* | 19.79 |
| TAK1 *S. aureus* | *TmColeC* | 27.76 | TAK1 *S. aureus* | *TmCec2* | 19.15 |
| TAK1 *S. aureus* | *TmL27a* | 22.32 | TAK1 *S. aureus* | *TmL27a* | 22.32 |
| TAK1 *S. aureus* | *TmL27a* | 22.04 | TAK1 *S. aureus* | *TmL27a* | 22.04 |
| EGFP *C. albicans* | *TmColeC* | 31.65 | EGFP *C. albicans* | *TmCec2* | 19.75 |
| EGFP *C. albicans* | *TmColeC* | 31.42 | EGFP *C. albicans* | *TmCec2* | 19.49 |
| EGFP *C. albicans* | *TmL27a* | 23.8 | EGFP *C. albicans* | *TmL27a* | 23.8 |
| EGFP *C. albicans* | *TmL27a* | 23.68 | EGFP *C. albicans* | *TmL27a* | 23.68 |
| TAK1 *C. albicans* | *TmColeC* | 29.1 | TAK1 *C. albicans* | *TmCec2* | 16.87 |
| TAK1 *C. albicans* | *TmColeC* | 29.86 | TAK1 *C. albicans* | *TmCec2* | 16.78 |
| TAK1 *C. albicans* | *TmL27a* | 21.01 | TAK1 *C. albicans* | *TmL27a* | 21.01 |
| TAK1 *C. albicans* | *TmL27a* | 20.94 | TAK1 *C. albicans* | *TmL27a* | 20.94 |

| Sample name | Gene name | Cq (∆R) | Sample name | Gene name | Cq (∆R) |
| --- | --- | --- | --- | --- | --- |
| EGFP PBS | *TmAtta1a* | 28.1 | EGFP PBS | *TmAtta1b* | 29.35 |
| EGFP PBS | *TmAtta1a* | 28.02 | EGFP PBS | *TmAtta1b* | 30.38 |
| EGFP PBS | *TmL27a* | 23.51 | EGFP PBS | *TmL27a* | 24.95 |
| EGFP PBS | *TmL27a* | 23.42 | EGFP PBS | *TmL27a* | 25.25 |
| TAK1 PBS | *TmAtta1a* | 26.93 | TAK1 PBS | *TmAtta1b* | 29 |
| TAK1 PBS | *TmAtta1a* | 26.83 | TAK1 PBS | *TmAtta1b* | 28.44 |
| TAK1 PBS | *TmL27a* | 23.27 | TAK1 PBS | *TmL27a* | 24.7 |
| TAK1 PBS | *TmL27a* | 23.03 | TAK1 PBS | *TmL27a* | 24.66 |
| EGFP *E. coli* | *TmAtta1a* | 24.61 | EGFP *E. coli* | *TmAtta1b* | 24.33 |
| EGFP *E. coli* | *TmAtta1a* | 24.38 | EGFP *E. coli* | *TmAtta1b* | 24.4 |
| EGFP *E. coli* | *TmL27a* | 23.66 | EGFP *E. coli* | *TmL27a* | 25.35 |
| EGFP *E. coli* | *TmL27a* | 23.55 | EGFP *E. coli* | *TmL27a* | 25.32 |
| TAK1 *E. coli* | *TmAtta1a* | 21.32 | TAK1 *E. coli* | *TmAtta1b* | 21.62 |
| TAK1 *E. coli* | *TmAtta1a* | 21.41 | TAK1 *E. coli* | *TmAtta1b* | 21.64 |
| TAK1 *E. coli* | *TmL27a* | 19.2 | TAK1 *E. coli* | *TmL27a* | 20.46 |
| TAK1 *E. coli* | *TmL27a* | 18.82 | TAK1 *E. coli* | *TmL27a* | 20.34 |
| EGFP *S. aureus* | *TmAtta1a* | 26.12 | EGFP *S. aureus* | *TmAtta1b* | 25.4 |
| EGFP *S. aureus* | *TmAtta1a* | 26.45 | EGFP *S. aureus* | *TmAtta1b* | 25.22 |
| EGFP *S. aureus* | *TmL27a* | 21.43 | EGFP *S. aureus* | *TmL27a* | 22.56 |
| EGFP *S. aureus* | *TmL27a* | 21.33 | EGFP *S. aureus* | *TmL27a* | 22.46 |
| TAK1 *S. aureus* | *TmAtta1a* | 25.97 | TAK1 *S. aureus* | *TmAtta1b* | 26 |
| TAK1 *S. aureus* | *TmAtta1a* | 26.28 | TAK1 *S. aureus* | *TmAtta1b* | 26.08 |
| TAK1 *S. aureus* | *TmL27a* | 21.39 | TAK1 *S. aureus* | *TmL27a* | 22.62 |
| TAK1 *S. aureus* | *TmL27a* | 21.28 | TAK1 *S. aureus* | *TmL27a* | 22.9 |
| EGFP *C. albicans* | *TmAtta1a* | 29.06 | EGFP *C. albicans* | *TmAtta1b* | 29.32 |
| EGFP *C. albicans* | *TmAtta1a* | 29.56 | EGFP *C. albicans* | *TmAtta1b* | 29.04 |
| EGFP *C. albicans* | *TmL27a* | 22.56 | EGFP *C. albicans* | *TmL27a* | 24.41 |
| EGFP *C. albicans* | *TmL27a* | 22.38 | EGFP *C. albicans* | *TmL27a* | 24.18 |
| TAK1 *C. albicans* | *TmAtta1a* | 27.16 | TAK1 *C. albicans* | *TmAtta1b* | 27.65 |
| TAK1 *C. albicans* | *TmAtta1a* | 27.17 | TAK1 *C. albicans* | *TmAtta1b* | 27.71 |
| TAK1 *C. albicans* | *TmL27a* | 20.12 | TAK1 *C. albicans* | *TmL27a* | 21.33 |
| TAK1 *C. albicans* | *TmL27a* | 20.1 | TAK1 *C. albicans* | *TmL27a* | 21.41 |

| Sample name | Gene name | Cq (∆R) | Sample name | Gene name | Cq (∆R) |
| --- | --- | --- | --- | --- | --- |
| EGFP PBS | *TmAtta2* | 29.14 | EGFP PBS | *TmTLP1* | 29.44 |
| EGFP PBS | *TmAtta2* | 29.38 | EGFP PBS | *TmTLP1* | 29.15 |
| EGFP PBS | *TmL27a* | 25.25 | EGFP PBS | *TmL27a* | 18.56 |
| EGFP PBS | *TmL27a* | 24.95 | EGFP PBS | *TmL27a* | 18.39 |
| TAK1 PBS | *TmAtta2* | 28.22 | TAK1 PBS | *TmTLP1* | 29.86 |
| TAK1 PBS | *TmAtta2* | 28.14 | TAK1 PBS | *TmTLP1* | 30.55 |
| TAK1 PBS | *TmL27a* | 24.7 | TAK1 PBS | *TmL27a* | 24.7 |
| TAK1 PBS | *TmL27a* | 24.66 | TAK1 PBS | *TmL27a* | 24.66 |
| EGFP *E. coli* | *TmAtta2* | 22.09 | EGFP *E. coli* | *TmTLP1* | 32.29 |
| EGFP *E. coli* | *TmAtta2* | 22.1 | EGFP *E. coli* | *TmTLP1* | 32.29 |
| EGFP *E. coli* | *TmL27a* | 25.35 | EGFP *E. coli* | *TmL27a* | 25.35 |
| EGFP *E. coli* | *TmL27a* | 25.32 | EGFP *E. coli* | *TmL27a* | 25.32 |
| TAK1 *E. coli* | *TmAtta2* | 18.47 | TAK1 *E. coli* | *TmTLP1* | 26.37 |
| TAK1 *E. coli* | *TmAtta2* | 18.47 | TAK1 *E. coli* | *TmTLP1* | 26.49 |
| TAK1 *E. coli* | *TmL27a* | 20.46 | TAK1 *E. coli* | *TmL27a* | 20.46 |
| TAK1 *E. coli* | *TmL27a* | 20.34 | TAK1 *E. coli* | *TmL27a* | 20.34 |
| EGFP *S. aureus* | *TmAtta2* | 22.34 | EGFP *S. aureus* | *TmTLP1* | 32.73 |
| EGFP *S. aureus* | *TmAtta2* | 22.31 | EGFP *S. aureus* | *TmTLP1* | 33.49 |
| EGFP *S. aureus* | *TmL27a* | 22.46 | EGFP *S. aureus* | *TmL27a* | 22.46 |
| EGFP *S. aureus* | *TmL27a* | 22.56 | EGFP *S. aureus* | *TmL27a* | 22.56 |
| TAK1 *S. aureus* | *TmAtta2* | 23.1 | TAK1 *S. aureus* | *TmTLP1* | 32.77 |
| TAK1 *S. aureus* | *TmAtta2* | 23.19 | TAK1 *S. aureus* | *TmTLP1* | 31.87 |
| TAK1 *S. aureus* | *TmL27a* | 22.9 | TAK1 *S. aureus* | *TmL27a* | 22.9 |
| TAK1 *S. aureus* | *TmL27a* | 22.62 | TAK1 *S. aureus* | *TmL27a* | 22.62 |
| EGFP *C. albicans* | *TmAtta2* | 26.81 | EGFP *C. albicans* | *TmTLP1* | 28.92 |
| EGFP *C. albicans* | *TmAtta2* | 26.76 | EGFP *C. albicans* | *TmTLP1* | 28.5 |
| EGFP *C. albicans* | *TmL27a* | 24.41 | EGFP *C. albicans* | *TmL27a* | 17.24 |
| EGFP *C. albicans* | *TmL27a* | 24.18 | EGFP *C. albicans* | *TmL27a* | 17.04 |
| TAK1 *C. albicans* | *TmAtta2* | 27.06 | TAK1 *C. albicans* | *TmTLP1* | 30.75 |
| TAK1 *C. albicans* | *TmAtta2* | 26.76 | TAK1 *C. albicans* | *TmTLP1* | 31.31 |
| TAK1 *C. albicans* | *TmL27a* | 21.33 | TAK1 *C. albicans* | *TmL27a* | 21.33 |
| TAK1 *C. albicans* | *TmL27a* | 21.41 | TAK1 *C. albicans* | *TmL27a* | 21.41 |

| Sample name | Gene name | Cq (∆R) |
| --- | --- | --- |
| EGFP PBS | *TmTLP2* | 30.75 |
| EGFP PBS | *TmTLP2* | 30.67 |
| EGFP PBS | *TmL27a* | 25.25 |
| EGFP PBS | *TmL27a* | 24.95 |
| TAK1 PBS | *TmTLP2* | 31.69 |
| TAK1 PBS | *TmTLP2* | 30.34 |
| TAK1 PBS | *TmL27a* | 24.7 |
| TAK1 PBS | *TmL27a* | 24.66 |
| EGFP *E. coli* | *TmTLP2* | 34.67 |
| EGFP *E. coli* | *TmTLP2* | 33.54 |
| EGFP *E. coli* | *TmL27a* | 25.35 |
| EGFP *E. coli* | *TmL27a* | 25.32 |
| TAK1 *E. coli* | *TmTLP2* | 25.36 |
| TAK1 *E. coli* | *TmTLP2* | 25.34 |
| TAK1 *E. coli* | *TmL27a* | 20.46 |
| TAK1 *E. coli* | *TmL27a* | 20.34 |
| EGFP *S. aureus* | *TmTLP2* | 32.07 |
| EGFP *S. aureus* | *TmTLP2* | 31.44 |
| EGFP *S. aureus* | *TmL27a* | 22.46 |
| EGFP *S. aureus* | *TmL27a* | 22.56 |
| TAK1 *S. aureus* | *TmTLP2* | 31.15 |
| TAK1 *S. aureus* | *TmTLP2* | 31.23 |
| TAK1 *S. aureus* | *TmL27a* | 22.9 |
| TAK1 *S. aureus* | *TmL27a* | 22.62 |
| EGFP *C. albicans* | *TmTLP2* | 33.03 |
| EGFP *C. albicans* | *TmTLP2* | 32.5 |
| EGFP *C. albicans* | *TmL27a* | 24.41 |
| EGFP *C. albicans* | *TmL27a* | 24.18 |
| TAK1 *C. albicans* | *TmTLP2* | 28.7 |
| TAK1 *C. albicans* | *TmTLP2* | 28.6 |
| TAK1 *C. albicans* | *TmL27a* | 21.33 |
| TAK1 *C. albicans* | *TmL27a* | 21.41 |

**Supplementary Table14. Raw data of qPCR in NF-Κb genes**.

Cq data of *TmTak1* and *TmL27a* in *T. molitor* at Gut(GT).

| Sample name | Gene name | Cq (∆R) | Sample name | Gene name | Cq (∆R) |
| --- | --- | --- | --- | --- | --- |
| EGFP PBS | *TmRelish* | 29.68 | EGFP PBS | *TmKayak* | 24.7 |
| EGFP PBS | *TmRelish* | 29.58 | EGFP PBS | *TmKayak* | 24.58 |
| EGFP PBS | *TmL27a* | 21.43 | EGFP PBS | *TmL27a* | 21.43 |
| EGFP PBS | *TmL27a* | 20.26 | EGFP PBS | *TmL27a* | 20.26 |
| TAK1 PBS | *TmRelish* | 29.03 | TAK1 PBS | *TmKayak* | 24.54 |
| TAK1 PBS | *TmRelish* | 28.95 | TAK1 PBS | *TmKayak* | 24.41 |
| TAK1 PBS | *TmL27a* | 20.44 | TAK1 PBS | *TmL27a* | 20.44 |
| TAK1 PBS | *TmL27a* | 20.44 | TAK1 PBS | *TmL27a* | 20.44 |
| EGFP *E. coli* | *TmRelish* | 28.51 | EGFP *E. coli* | *TmKayak* | 24.06 |
| EGFP *E. coli* | *TmRelish* | 28.39 | EGFP *E. coli* | *TmKayak* | 23.98 |
| EGFP *E. coli* | *TmL27a* | 20.7 | EGFP *E. coli* | *TmL27a* | 20.7 |
| EGFP *E. coli* | *TmL27a* | 20.42 | EGFP *E. coli* | *TmL27a* | 20.42 |
| TAK1 *E. coli* | *TmRelish* | 29.1 | TAK1 *E. coli* | *TmKayak* | 24.57 |
| TAK1 *E. coli* | *TmRelish* | 28.95 | TAK1 *E. coli* | *TmKayak* | 24.53 |
| TAK1 *E. coli* | *TmL27a* | 21.01 | TAK1 *E. coli* | *TmL27a* | 21.01 |
| TAK1 *E. coli* | *TmL27a* | 20.48 | TAK1 *E. coli* | *TmL27a* | 20.48 |
| EGFP *S. aureus* | *TmRelish* | 29.98 | EGFP *S. aureus* | *TmKayak* | 25.34 |
| EGFP *S. aureus* | *TmRelish* | 29.55 | EGFP *S. aureus* | *TmKayak* | 25.28 |
| EGFP *S. aureus* | *TmL27a* | 21.19 | EGFP *S. aureus* | *TmL27a* | 21.19 |
| EGFP *S. aureus* | *TmL27a* | 20.89 | EGFP *S. aureus* | *TmL27a* | 20.89 |
| TAK1 *S. aureus* | *TmRelish* | 28.07 | TAK1 *S. aureus* | *TmKayak* | 23.3 |
| TAK1 *S. aureus* | *TmRelish* | 27.91 | TAK1 *S. aureus* | *TmKayak* | 23.28 |
| TAK1 *S. aureus* | *TmL27a* | 19.21 | TAK1 *S. aureus* | *TmL27a* | 19.21 |
| TAK1 *S. aureus* | *TmL27a* | 18.88 | TAK1 *S. aureus* | *TmL27a* | 18.88 |
| EGFP *C. albicans* | *TmRelish* | 27.29 | EGFP *C. albicans* | *TmKayak* | 23.13 |
| EGFP *C. albicans* | *TmRelish* | 27.3 | EGFP *C. albicans* | *TmKayak* | 23.21 |
| EGFP *C. albicans* | *TmL27a* | 18.86 | EGFP *C. albicans* | *TmL27a* | 18.86 |
| EGFP *C. albicans* | *TmL27a* | 19.08 | EGFP *C. albicans* | *TmL27a* | 19.08 |
| TAK1 *C. albicans* | *TmRelish* | 28.85 | TAK1 *C. albicans* | *TmKayak* | 24.63 |
| TAK1 *C. albicans* | *TmRelish* | 28.82 | TAK1 *C. albicans* | *TmKayak* | 24.61 |
| TAK1 *C. albicans* | *TmL27a* | 20.63 | TAK1 *C. albicans* | *TmL27a* | 20.63 |
| TAK1 *C. albicans* | *TmL27a* | 20.56 | TAK1 *C. albicans* | *TmL27a* | 20.56 |

| Sample name | Gene name | Cq (∆R) | Sample name | Gene name | Cq (∆R) |
| --- | --- | --- | --- | --- | --- |
| EGFP PBS | *TmDorX1* | 27.33 | EGFP PBS | *TmDorX2* | 31.45 |
| EGFP PBS | *TmDorX1* | 27.29 | EGFP PBS | *TmDorX2* | 31.05 |
| EGFP PBS | *TmL27a* | 21.43 | EGFP PBS | *TmL27a* | 21.43 |
| EGFP PBS | *TmL27a* | 20.26 | EGFP PBS | *TmL27a* | 20.26 |
| TAK1 PBS | *TmDorX1* | 24.43 | TAK1 PBS | *TmDorX2* | 28.6 |
| TAK1 PBS | *TmDorX1* | 24.19 | TAK1 PBS | *TmDorX2* | 29.58 |
| TAK1 PBS | *TmL27a* | 20.44 | TAK1 PBS | *TmL27a* | 20.44 |
| TAK1 PBS | *TmL27a* | 20.44 | TAK1 PBS | *TmL27a* | 20.44 |
| EGFP *E. coli* | *TmDorX1* | 27.39 | EGFP *E. coli* | *TmDorX2* | 26.8 |
| EGFP *E. coli* | *TmDorX1* | 27.8 | EGFP *E. coli* | *TmDorX2* | 26.93 |
| EGFP *E. coli* | *TmL27a* | 20.7 | EGFP *E. coli* | *TmL27a* | 20.7 |
| EGFP *E. coli* | *TmL27a* | 20.42 | EGFP *E. coli* | *TmL27a* | 20.42 |
| TAK1 *E. coli* | *TmDorX1* | 26.2 | TAK1 *E. coli* | *TmDorX2* | 27.38 |
| TAK1 *E. coli* | *TmDorX1* | 26.21 | TAK1 *E. coli* | *TmDorX2* | 28.09 |
| TAK1 *E. coli* | *TmL27a* | 21.01 | TAK1 *E. coli* | *TmL27a* | 21.01 |
| TAK1 *E. coli* | *TmL27a* | 20.48 | TAK1 *E. coli* | *TmL27a* | 20.48 |
| EGFP *S. aureus* | *TmDorX1* | 28.43 | EGFP *S. aureus* | *TmDorX2* | 30.54 |
| EGFP *S. aureus* | *TmDorX1* | 28.36 | EGFP *S. aureus* | *TmDorX2* | 30.48 |
| EGFP *S. aureus* | *TmL27a* | 21.19 | EGFP *S. aureus* | *TmL27a* | 21.19 |
| EGFP *S. aureus* | *TmL27a* | 20.89 | EGFP *S. aureus* | *TmL27a* | 20.89 |
| TAK1 *S. aureus* | *TmDorX1* | 24.01 | TAK1 *S. aureus* | *TmDorX2* | 28.46 |
| TAK1 *S. aureus* | *TmDorX1* | 24.01 | TAK1 *S. aureus* | *TmDorX2* | 28.65 |
| TAK1 *S. aureus* | *TmL27a* | 19.21 | TAK1 *S. aureus* | *TmL27a* | 19.21 |
| TAK1 *S. aureus* | *TmL27a* | 18.88 | TAK1 *S. aureus* | *TmL27a* | 18.88 |
| EGFP *C. albicans* | *TmDorX1* | 25.07 | EGFP *C. albicans* | *TmDorX2* | 26.31 |
| EGFP *C. albicans* | *TmDorX1* | 25.19 | EGFP *C. albicans* | *TmDorX2* | 27 |
| EGFP *C. albicans* | *TmL27a* | 18.86 | EGFP *C. albicans* | *TmL27a* | 18.86 |
| EGFP *C. albicans* | *TmL27a* | 19.08 | EGFP *C. albicans* | *TmL27a* | 19.08 |
| TAK1 *C. albicans* | *TmDorX1* | 28.54 | TAK1 *C. albicans* | *TmDorX2* | 30.81 |
| TAK1 *C. albicans* | *TmDorX1* | 28.23 | TAK1 *C. albicans* | *TmDorX2* | 30.42 |
| TAK1 *C. albicans* | *TmL27a* | 20.63 | TAK1 *C. albicans* | *TmL27a* | 20.63 |
| TAK1 *C. albicans* | *TmL27a* | 20.56 | TAK1 *C. albicans* | *TmL27a* | 20.56 |

**Supplementary Table15. Raw data of qPCR in NF-Κb genes**.

Cq data of *TmTak1* and *TmL27a* in *T. molitor* at integument(IT).

| Sample name | Gene name | Cq (∆R) | Sample name | Gene name | Cq (∆R) |
| --- | --- | --- | --- | --- | --- |
| EGFP PBS | *TmRelish* | 30.68 | EGFP PBS | *TmKayak* | 20.98 |
| EGFP PBS | *TmRelish* | 31.84 | EGFP PBS | *TmKayak* | 20.84 |
| EGFP PBS | *TmL27a* | 19.67 | EGFP PBS | *TmL27a* | 19.67 |
| EGFP PBS | *TmL27a* | 19.9 | EGFP PBS | *TmL27a* | 19.9 |
| TAK1 PBS | *TmRelish* | 32.07 | TAK1 PBS | *TmKayak* | 22.09 |
| TAK1 PBS | *TmRelish* | 32.07 | TAK1 PBS | *TmKayak* | 21.44 |
| TAK1 PBS | *TmL27a* | 19.72 | TAK1 PBS | *TmL27a* | 19.72 |
| TAK1 PBS | *TmL27a* | 19.74 | TAK1 PBS | *TmL27a* | 19.74 |
| EGFP *E. coli* | *TmRelish* | 29.38 | EGFP *E. coli* | *TmKayak* | 21.03 |
| EGFP *E. coli* | *TmRelish* | 30.32 | EGFP *E. coli* | *TmKayak* | 20.99 |
| EGFP *E. coli* | *TmL27a* | 19.19 | EGFP *E. coli* | *TmL27a* | 19.19 |
| EGFP *E. coli* | *TmL27a* | 19.28 | EGFP *E. coli* | *TmL27a* | 19.28 |
| TAK1 *E. coli* | *TmRelish* | 29.23 | TAK1 *E. coli* | *TmKayak* | 20.92 |
| TAK1 *E. coli* | *TmRelish* | 30.18 | TAK1 *E. coli* | *TmKayak* | 20.65 |
| TAK1 *E. coli* | *TmL27a* | 19.08 | TAK1 *E. coli* | *TmL27a* | 19.08 |
| TAK1 *E. coli* | *TmL27a* | 19.08 | TAK1 *E. coli* | *TmL27a* | 19.08 |
| EGFP *S. aureus* | *TmRelish* | 31.36 | EGFP *S. aureus* | *TmKayak* | 21.34 |
| EGFP *S. aureus* | *TmRelish* | 32.37 | EGFP *S. aureus* | *TmKayak* | 21.42 |
| EGFP *S. aureus* | *TmL27a* | 20.28 | EGFP *S. aureus* | *TmL27a* | 20.28 |
| EGFP *S. aureus* | *TmL27a* | 20.23 | EGFP *S. aureus* | *TmL27a* | 20.23 |
| TAK1 *S. aureus* | *TmRelish* | 32.03 | TAK1 *S. aureus* | *TmKayak* | 21.58 |
| TAK1 *S. aureus* | *TmRelish* | 32.47 | TAK1 *S. aureus* | *TmKayak* | 21.52 |
| TAK1 *S. aureus* | *TmL27a* | 20.07 | TAK1 *S. aureus* | *TmL27a* | 20.07 |
| TAK1 *S. aureus* | *TmL27a* | 20.08 | TAK1 *S. aureus* | *TmL27a* | 20.08 |
| EGFP *C. albicans* | *TmRelish* | 31.15 | EGFP *C. albicans* | *TmKayak* | 21.74 |
| EGFP *C. albicans* | *TmRelish* | 31.75 | EGFP *C. albicans* | *TmKayak* | 21.44 |
| EGFP *C. albicans* | *TmL27a* | 19.74 | EGFP *C. albicans* | *TmL27a* | 19.74 |
| EGFP *C. albicans* | *TmL27a* | 19.98 | EGFP *C. albicans* | *TmL27a* | 19.98 |
| TAK1 *C. albicans* | *TmRelish* | 31.78 | TAK1 *C. albicans* | *TmKayak* | 21.38 |
| TAK1 *C. albicans* | *TmRelish* | 31.78 | TAK1 *C. albicans* | *TmKayak* | 21.7 |
| TAK1 *C. albicans* | *TmL27a* | 19.56 | TAK1 *C. albicans* | *TmL27a* | 19.56 |
| TAK1 *C. albicans* | *TmL27a* | 19.84 | TAK1 *C. albicans* | *TmL27a* | 19.84 |

| Sample name | Gene name | Cq (∆R) | Sample name | Gene name | Cq (∆R) |
| --- | --- | --- | --- | --- | --- |
| EGFP PBS | *TmDorX1* | 25.09 | EGFP PBS | *TmDorX2* | 26.25 |
| EGFP PBS | *TmDorX1* | 25.48 | EGFP PBS | *TmDorX2* | 26.76 |
| EGFP PBS | *TmL27a* | 19.67 | EGFP PBS | *TmL27a* | 19.67 |
| EGFP PBS | *TmL27a* | 19.9 | EGFP PBS | *TmL27a* | 19.9 |
| TAK1 PBS | *TmDorX1* | 26.84 | TAK1 PBS | *TmDorX2* | 27.36 |
| TAK1 PBS | *TmDorX1* | 26.9 | TAK1 PBS | *TmDorX2* | 27.14 |
| TAK1 PBS | *TmL27a* | 19.72 | TAK1 PBS | *TmL27a* | 19.72 |
| TAK1 PBS | *TmL27a* | 19.74 | TAK1 PBS | *TmL27a* | 19.74 |
| EGFP *E. coli* | *TmDorX1* | 25.1 | EGFP *E. coli* | *TmDorX2* | 24.01 |
| EGFP *E. coli* | *TmDorX1* | 24.75 | EGFP *E. coli* | *TmDorX2* | 24.12 |
| EGFP *E. coli* | *TmL27a* | 19.19 | EGFP *E. coli* | *TmL27a* | 19.19 |
| EGFP *E. coli* | *TmL27a* | 19.28 | EGFP *E. coli* | *TmL27a* | 19.28 |
| TAK1 *E. coli* | *TmDorX1* | 26.12 | TAK1 *E. coli* | *TmDorX2* | 25.43 |
| TAK1 *E. coli* | *TmDorX1* | 26.25 | TAK1 *E. coli* | *TmDorX2* | 25.89 |
| TAK1 *E. coli* | *TmL27a* | 19.08 | TAK1 *E. coli* | *TmL27a* | 19.08 |
| TAK1 *E. coli* | *TmL27a* | 19.08 | TAK1 *E. coli* | *TmL27a* | 19.08 |
| EGFP *S. aureus* | *TmDorX1* | 25.55 | EGFP *S. aureus* | *TmDorX2* | 26.69 |
| EGFP *S. aureus* | *TmDorX1* | 25.62 | EGFP *S. aureus* | *TmDorX2* | 26.64 |
| EGFP *S. aureus* | *TmL27a* | 20.28 | EGFP *S. aureus* | *TmL27a* | 20.28 |
| EGFP *S. aureus* | *TmL27a* | 20.23 | EGFP *S. aureus* | *TmL27a* | 20.23 |
| TAK1 *S. aureus* | *TmDorX1* | 28.21 | TAK1 *S. aureus* | *TmDorX2* | 28.13 |
| TAK1 *S. aureus* | *TmDorX1* | 28.44 | TAK1 *S. aureus* | *TmDorX2* | 27.89 |
| TAK1 *S. aureus* | *TmL27a* | 20.07 | TAK1 *S. aureus* | *TmL27a* | 20.07 |
| TAK1 *S. aureus* | *TmL27a* | 20.08 | TAK1 *S. aureus* | *TmL27a* | 20.08 |
| EGFP *C. albicans* | *TmDorX1* | 26.26 | EGFP *C. albicans* | *TmDorX2* | 26.87 |
| EGFP *C. albicans* | *TmDorX1* | 26.54 | EGFP *C. albicans* | *TmDorX2* | 27.15 |
| EGFP *C. albicans* | *TmL27a* | 19.74 | EGFP *C. albicans* | *TmL27a* | 19.74 |
| EGFP *C. albicans* | *TmL27a* | 19.98 | EGFP *C. albicans* | *TmL27a* | 19.98 |
| TAK1 *C. albicans* | *TmDorX1* | 28.14 | TAK1 *C. albicans* | *TmDorX2* | 28.25 |
| TAK1 *C. albicans* | *TmDorX1* | 27.68 | TAK1 *C. albicans* | *TmDorX2* | 28.08 |
| TAK1 *C. albicans* | *TmL27a* | 19.56 | TAK1 *C. albicans* | *TmL27a* | 19.56 |
| TAK1 *C. albicans* | *TmL27a* | 19.84 | TAK1 *C. albicans* | *TmL27a* | 19.84 |

**Supplementary Table16. Raw data of qPCR in NF-Κb genes**.

Cq data of *TmTak1* and *TmL27a* in *T. molitor* at Malpighian tubules(MT).

| Sample name | Gene name | Cq (∆R) | Sample name | Gene name | Cq (∆R) |
| --- | --- | --- | --- | --- | --- |
| EGFP PBS | *TmRelish* | 30.46 | EGFP PBS | *TmKayak* | 26.35 |
| EGFP PBS | *TmRelish* | 30.48 | EGFP PBS | *TmKayak* | 26.51 |
| EGFP PBS | *TmL27a* | 20.88 | EGFP PBS | *TmL27a* | 21.93 |
| EGFP PBS | *TmL27a* | 21.01 | EGFP PBS | *TmL27a* | 21.61 |
| TAK1 PBS | *TmRelish* | 30.97 | TAK1 PBS | *TmKayak* | 27.79 |
| TAK1 PBS | *TmRelish* | 31.38 | TAK1 PBS | *TmKayak* | 27.6 |
| TAK1 PBS | *TmL27a* | 20.9 | TAK1 PBS | *TmL27a* | 22.84 |
| TAK1 PBS | *TmL27a* | 21.31 | TAK1 PBS | *TmL27a* | 22.72 |
| EGFP *E. coli* | *TmRelish* | 29.99 | EGFP *E. coli* | *TmKayak* | 25.65 |
| EGFP *E. coli* | *TmRelish* | 29.99 | EGFP *E. coli* | *TmKayak* | 25.53 |
| EGFP *E. coli* | *TmL27a* | 23.13 | EGFP *E. coli* | *TmL27a* | 23.13 |
| EGFP *E. coli* | *TmL27a* | 22.8 | EGFP *E. coli* | *TmL27a* | 22.8 |
| TAK1 *E. coli* | *TmRelish* | 28.81 | TAK1 *E. coli* | *TmKayak* | 23.89 |
| TAK1 *E. coli* | *TmRelish* | 28.77 | TAK1 *E. coli* | *TmKayak* | 23.69 |
| TAK1 *E. coli* | *TmL27a* | 19.87 | TAK1 *E. coli* | *TmL27a* | 19.87 |
| TAK1 *E. coli* | *TmL27a* | 19.72 | TAK1 *E. coli* | *TmL27a* | 19.72 |
| EGFP *S. aureus* | *TmRelish* | 29.22 | EGFP *S. aureus* | *TmKayak* | 23.32 |
| EGFP *S. aureus* | *TmRelish* | 29.32 | EGFP *S. aureus* | *TmKayak* | 23.31 |
| EGFP *S. aureus* | *TmL27a* | 20.17 | EGFP *S. aureus* | *TmL27a* | 20.17 |
| EGFP *S. aureus* | *TmL27a* | 19.98 | EGFP *S. aureus* | *TmL27a* | 19.98 |
| TAK1 *S. aureus* | *TmRelish* | 27.98 | TAK1 *S. aureus* | *TmKayak* | 22.14 |
| TAK1 *S. aureus* | *TmRelish* | 28.37 | TAK1 *S. aureus* | *TmKayak* | 22.05 |
| TAK1 *S. aureus* | *TmL27a* | 18.43 | TAK1 *S. aureus* | *TmL27a* | 18.43 |
| TAK1 *S. aureus* | *TmL27a* | 18.23 | TAK1 *S. aureus* | *TmL27a* | 18.23 |
| EGFP *C. albicans* | *TmRelish* | 30.6 | EGFP *C. albicans* | *TmKayak* | 26.06 |
| EGFP *C. albicans* | *TmRelish* | 30.56 | EGFP *C. albicans* | *TmKayak* | 25.54 |
| EGFP *C. albicans* | *TmL27a* | 21.98 | EGFP *C. albicans* | *TmL27a* | 21.98 |
| EGFP *C. albicans* | *TmL27a* | 21.77 | EGFP *C. albicans* | *TmL27a* | 21.77 |
| TAK1 *C. albicans* | *TmRelish* | 28.24 | TAK1 *C. albicans* | *TmKayak* | 23.36 |
| TAK1 *C. albicans* | *TmRelish* | 28.18 | TAK1 *C. albicans* | *TmKayak* | 23.01 |
| TAK1 *C. albicans* | *TmL27a* | 18.59 | TAK1 *C. albicans* | *TmL27a* | 18.59 |
| TAK1 *C. albicans* | *TmL27a* | 18.51 | TAK1 *C. albicans* | *TmL27a* | 18.51 |

| Sample name | Gene name | Cq (∆R) | Sample name | Gene name | Cq (∆R) |
| --- | --- | --- | --- | --- | --- |
| EGFP PBS | *TmDorX1* | 28.45 | EGFP PBS | *TmDorX2* | 29.26 |
| EGFP PBS | *TmDorX1* | 28.8 | EGFP PBS | *TmDorX2* | 29.19 |
| EGFP PBS | *TmL27a* | 21.93 | EGFP PBS | *TmL27a* | 21.93 |
| EGFP PBS | *TmL27a* | 21.61 | EGFP PBS | *TmL27a* | 21.61 |
| TAK1 PBS | *TmDorX1* | 28.2 | TAK1 PBS | *TmDorX2* | 30.3 |
| TAK1 PBS | *TmDorX1* | 28.3 | TAK1 PBS | *TmDorX2* | 29.07 |
| TAK1 PBS | *TmL27a* | 22.84 | TAK1 PBS | *TmL27a* | 22.84 |
| TAK1 PBS | *TmL27a* | 22.72 | TAK1 PBS | *TmL27a* | 22.72 |
| EGFP *E. coli* | *TmDorX1* | 28.32 | EGFP *E. coli* | *TmDorX2* | 27.39 |
| EGFP *E. coli* | *TmDorX1* | 28.3 | EGFP *E. coli* | *TmDorX2* | 27.03 |
| EGFP *E. coli* | *TmL27a* | 23.13 | EGFP *E. coli* | *TmL27a* | 23.13 |
| EGFP *E. coli* | *TmL27a* | 22.8 | EGFP *E. coli* | *TmL27a* | 22.8 |
| TAK1 *E. coli* | *TmDorX1* | 25.89 | TAK1 *E. coli* | *TmDorX2* | 24.21 |
| TAK1 *E. coli* | *TmDorX1* | 25.61 | TAK1 *E. coli* | *TmDorX2* | 24.35 |
| TAK1 *E. coli* | *TmL27a* | 19.87 | TAK1 *E. coli* | *TmL27a* | 19.87 |
| TAK1 *E. coli* | *TmL27a* | 19.72 | TAK1 *E. coli* | *TmL27a* | 19.72 |
| EGFP *S. aureus* | *TmDorX1* | 26.66 | EGFP *S. aureus* | *TmDorX2* | 24.93 |
| EGFP *S. aureus* | *TmDorX1* | 26.51 | EGFP *S. aureus* | *TmDorX2* | 25.06 |
| EGFP *S. aureus* | *TmL27a* | 20.17 | EGFP *S. aureus* | *TmL27a* | 20.17 |
| EGFP *S. aureus* | *TmL27a* | 19.98 | EGFP *S. aureus* | *TmL27a* | 19.98 |
| TAK1 *S. aureus* | *TmDorX1* | 25.43 | TAK1 *S. aureus* | *TmDorX2* | 23.46 |
| TAK1 *S. aureus* | *TmDorX1* | 25.22 | TAK1 *S. aureus* | *TmDorX2* | 23.51 |
| TAK1 *S. aureus* | *TmL27a* | 18.43 | TAK1 *S. aureus* | *TmL27a* | 18.43 |
| TAK1 *S. aureus* | *TmL27a* | 18.23 | TAK1 *S. aureus* | *TmL27a* | 18.23 |
| EGFP *C. albicans* | *TmDorX1* | 27.28 | EGFP *C. albicans* | *TmDorX2* | 26.32 |
| EGFP *C. albicans* | *TmDorX1* | 27.46 | EGFP *C. albicans* | *TmDorX2* | 25.97 |
| EGFP *C. albicans* | *TmL27a* | 21.98 | EGFP *C. albicans* | *TmL27a* | 21.98 |
| EGFP *C. albicans* | *TmL27a* | 21.77 | EGFP *C. albicans* | *TmL27a* | 21.77 |
| TAK1 *C. albicans* | *TmDorX1* | 25.68 | TAK1 *C. albicans* | *TmDorX2* | 24.52 |
| TAK1 *C. albicans* | *TmDorX1* | 25.49 | TAK1 *C. albicans* | *TmDorX2* | 24.52 |
| TAK1 *C. albicans* | *TmL27a* | 18.59 | TAK1 *C. albicans* | *TmL27a* | 18.59 |
| TAK1 *C. albicans* | *TmL27a* | 18.51 | TAK1 *C. albicans* | *TmL27a* | 18.51 |

**Supplementary Table17. Raw data of qPCR in NF-Κb genes**.

Cq data of *TmTak1* and *TmL27a* in *T. molitor* at Hemocytes(HC).

| Sample name | Gene name | Cq (∆R) | Sample name | Gene name | Cq (∆R) |
| --- | --- | --- | --- | --- | --- |
| EGFP PBS | *TmRelish* | 35.09 | EGFP PBS | *TmKayak* | 31.37 |
| EGFP PBS | *TmRelish* | 35.09 | EGFP PBS | *TmKayak* | 30.76 |
| EGFP PBS | *TmL27a* | 25.13 | EGFP PBS | *TmL27a* | 25.13 |
| EGFP PBS | *TmL27a* | 25.03 | EGFP PBS | *TmL27a* | 25.03 |
| TAK1 PBS | *TmRelish* | 34.6 | TAK1 PBS | *TmKayak* | 30.56 |
| TAK1 PBS | *TmRelish* | 34.08 | TAK1 PBS | *TmKayak* | 30.73 |
| TAK1 PBS | *TmL27a* | 24.8 | TAK1 PBS | *TmL27a* | 24.8 |
| TAK1 PBS | *TmL27a* | 24.92 | TAK1 PBS | *TmL27a* | 24.92 |
| EGFP *E. coli* | *TmRelish* | 34.26 | EGFP *E. coli* | *TmKayak* | 30.99 |
| EGFP *E. coli* | *TmRelish* | 34.92 | EGFP *E. coli* | *TmKayak* | 31.78 |
| EGFP *E. coli* | *TmL27a* | 24.9 | EGFP *E. coli* | *TmL27a* | 24.9 |
| EGFP *E. coli* | *TmL27a* | 25.09 | EGFP *E. coli* | *TmL27a* | 25.09 |
| TAK1 *E. coli* | *TmRelish* | 30.65 | TAK1 *E. coli* | *TmKayak* | 26.48 |
| TAK1 *E. coli* | *TmRelish* | 30.04 | TAK1 *E. coli* | *TmKayak* | 26.32 |
| TAK1 *E. coli* | *TmL27a* | 20.28 | TAK1 *E. coli* | *TmL27a* | 20.28 |
| TAK1 *E. coli* | *TmL27a* | 20.04 | TAK1 *E. coli* | *TmL27a* | 20.04 |
| EGFP *S. aureus* | *TmRelish* | 33.01 | EGFP *S. aureus* | *TmKayak* | 28.54 |
| EGFP *S. aureus* | *TmRelish* | 33.38 | EGFP *S. aureus* | *TmKayak* | 28.79 |
| EGFP *S. aureus* | *TmL27a* | 22.69 | EGFP *S. aureus* | *TmL27a* | 22.69 |
| EGFP *S. aureus* | *TmL27a* | 22.88 | EGFP *S. aureus* | *TmL27a* | 22.88 |
| TAK1 *S. aureus* | *TmRelish* | 33.04 | TAK1 *S. aureus* | *TmKayak* | 29.22 |
| TAK1 *S. aureus* | *TmRelish* | 32.87 | TAK1 *S. aureus* | *TmKayak* | 29.24 |
| TAK1 *S. aureus* | *TmL27a* | 22.83 | TAK1 *S. aureus* | *TmL27a* | 22.83 |
| TAK1 *S. aureus* | *TmL27a* | 22.71 | TAK1 *S. aureus* | *TmL27a* | 22.71 |
| EGFP *C. albicans* | *TmRelish* | 33.28 | EGFP *C. albicans* | *TmKayak* | 29.91 |
| EGFP *C. albicans* | *TmRelish* | 34.36 | EGFP *C. albicans* | *TmKayak* | 30.4 |
| EGFP *C. albicans* | *TmL27a* | 23.93 | EGFP *C. albicans* | *TmL27a* | 23.93 |
| EGFP *C. albicans* | *TmL27a* | 23.81 | EGFP *C. albicans* | *TmL27a* | 23.81 |
| TAK1 *C. albicans* | *TmRelish* | 32.13 | TAK1 *C. albicans* | *TmKayak* | 26.75 |
| TAK1 *C. albicans* | *TmRelish* | 33.14 | TAK1 *C. albicans* | *TmKayak* | 27.23 |
| TAK1 *C. albicans* | *TmL27a* | 21.33 | TAK1 *C. albicans* | *TmL27a* | 21.33 |
| TAK1 *C. albicans* | *TmL27a* | 21.56 | TAK1 *C. albicans* | *TmL27a* | 21.56 |

| Sample name | Gene name | Cq (∆R) | Sample name | Gene name | Cq (∆R) |
| --- | --- | --- | --- | --- | --- |
| EGFP PBS | *TmDorX1* | 30.32 | EGFP PBS | *TmDorX2* | 34.04 |
| EGFP PBS | *TmDorX1* | 30.17 | EGFP PBS | *TmDorX2* | 33.01 |
| EGFP PBS | *TmL27a* | 25.13 | EGFP PBS | *TmL27a* | 25.13 |
| EGFP PBS | *TmL27a* | 25.03 | EGFP PBS | *TmL27a* | 25.03 |
| TAK1 PBS | *TmDorX1* | 29.62 | TAK1 PBS | *TmDorX2* | 33.02 |
| TAK1 PBS | *TmDorX1* | 29.63 | TAK1 PBS | *TmDorX2* | 33.66 |
| TAK1 PBS | *TmL27a* | 24.8 | TAK1 PBS | *TmL27a* | 24.8 |
| TAK1 PBS | *TmL27a* | 24.92 | TAK1 PBS | *TmL27a* | 24.92 |
| EGFP *E. coli* | *TmDorX1* | 31.05 | EGFP *E. coli* | *TmDorX2* | 33.06 |
| EGFP *E. coli* | *TmDorX1* | 31.67 | EGFP *E. coli* | *TmDorX2* | 32.29 |
| EGFP *E. coli* | *TmL27a* | 24.9 | EGFP *E. coli* | *TmL27a* | 24.9 |
| EGFP *E. coli* | *TmL27a* | 25.09 | EGFP *E. coli* | *TmL27a* | 25.09 |
| TAK1 *E. coli* | *TmDorX1* | 24.63 | TAK1 *E. coli* | *TmDorX2* | 27.24 |
| TAK1 *E. coli* | *TmDorX1* | 24.45 | TAK1 *E. coli* | *TmDorX2* | 27.04 |
| TAK1 *E. coli* | *TmL27a* | 20.28 | TAK1 *E. coli* | *TmL27a* | 20.28 |
| TAK1 *E. coli* | *TmL27a* | 20.04 | TAK1 *E. coli* | *TmL27a* | 20.04 |
| EGFP *S. aureus* | *TmDorX1* | 29.88 | EGFP *S. aureus* | *TmDorX2* | 30.31 |
| EGFP *S. aureus* | *TmDorX1* | 29.28 | EGFP *S. aureus* | *TmDorX2* | 31.23 |
| EGFP *S. aureus* | *TmL27a* | 22.69 | EGFP *S. aureus* | *TmL27a* | 22.69 |
| EGFP *S. aureus* | *TmL27a* | 22.88 | EGFP *S. aureus* | *TmL27a* | 22.88 |
| TAK1 *S. aureus* | *TmDorX1* | 29.4 | TAK1 *S. aureus* | *TmDorX2* | 31.04 |
| TAK1 *S. aureus* | *TmDorX1* | 29.65 | TAK1 *S. aureus* | *TmDorX2* | 32.15 |
| TAK1 *S. aureus* | *TmL27a* | 22.83 | TAK1 *S. aureus* | *TmL27a* | 22.83 |
| TAK1 *S. aureus* | *TmL27a* | 22.71 | TAK1 *S. aureus* | *TmL27a* | 22.71 |
| EGFP *C. albicans* | *TmDorX1* | 31.11 | EGFP *C. albicans* | *TmDorX2* | 32.21 |
| EGFP *C. albicans* | *TmDorX1* | 30.38 | EGFP *C. albicans* | *TmDorX2* | 32.07 |
| EGFP *C. albicans* | *TmL27a* | 23.93 | EGFP *C. albicans* | *TmL27a* | 23.93 |
| EGFP *C. albicans* | *TmL27a* | 23.81 | EGFP *C. albicans* | *TmL27a* | 23.81 |
| TAK1 *C. albicans* | *TmDorX1* | 28.33 | TAK1 *C. albicans* | *TmDorX2* | 28.81 |
| TAK1 *C. albicans* | *TmDorX1* | 28.86 | TAK1 *C. albicans* | *TmDorX2* | 29.28 |
| TAK1 *C. albicans* | *TmL27a* | 21.33 | TAK1 *C. albicans* | *TmL27a* | 21.33 |
| TAK1 *C. albicans* | *TmL27a* | 21.56 | TAK1 *C. albicans* | *TmL27a* | 21.56 |
